# Supplementary material for: Deubiquitinating enzyme mutagenesis screens identify a USP43-dependent HIF-1 transcriptional response
Source: EMBO J. 2024 Jul 15;43(17):8. doi: 10.1038/s44318-024-00166-6 (PMC11377827; doi:10.1038/s44318-024-00166-6)
Supplement: Supplementary file 1 — Appendix [file 44318_2024_166_MOESM1_ESM.pdf]

Appendix: Deubiquitinating enzyme mutagenesis screens identify a USP43-dependent HIF-1 transcriptional response

Table of Contents

	Page
**Appendix Figure S1: USP43 depletion delays activation of a HIF response.**	**2**
**Appendix Figure S2: USP43 is specific for the HIF-1 complex.**	**4**
**Appendix Figure S3: Reconstituting USP43 deficiency restores HIF signalling.**	**5**
**Appendix Figure S4: USP43 depletion does not alter H2B^{K120Ub} levels.**	**6**
**Appendix Figure S5: Gating strategy.**	**7**

Appendix Figure S1. USP43 depletion delays activation of a HIF response.

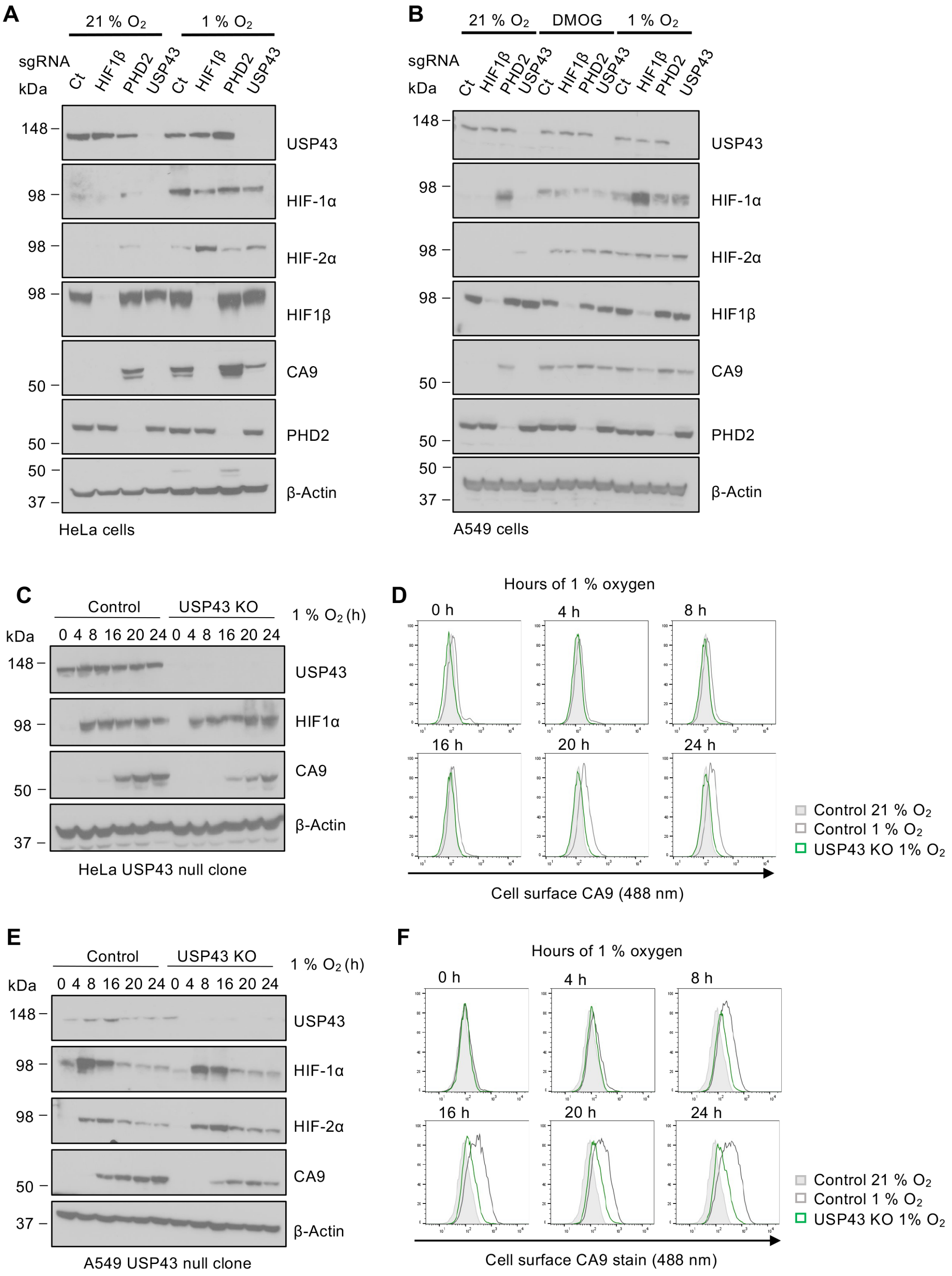

A) Control (Ct) or mixed KO populations of HIF1β, PHD2, or USP43 KO HeLa cells were incubated in 21 % or 1 % oxygen and immunoblotted for components of the HIF pathway and the HIF-1 target, CA9. Representative of three biological replicates. **B)** Control or mixed KO populations of HIF1β, PHD2, or USP43 KO A549 cells were incubated in 21 %, 1 % oxygen, or treated with 1 mM DMOG, and immunoblotted for components of the HIF pathway and the HIF-1 target, CA9. Representative of three biological replicates. **C)** HeLa control cells or a HeLa USP43 null clone were incubated in 0-24 h of 1 % oxygen and analysed by immunoblotting. Representative of three biological replicates. **D)** Cell surface

CA9 levels in control or mixed KO populations of USP43 HeLa cells incubated in 0-24 h of 1 % oxygen. Plots are representatives of three biological replicates. **E, F)** As for **(C, D)** but using A549 cells. Representative of three biological replicates.

Appendix Figure S2. USP43 is specific for the HIF-1 complex.

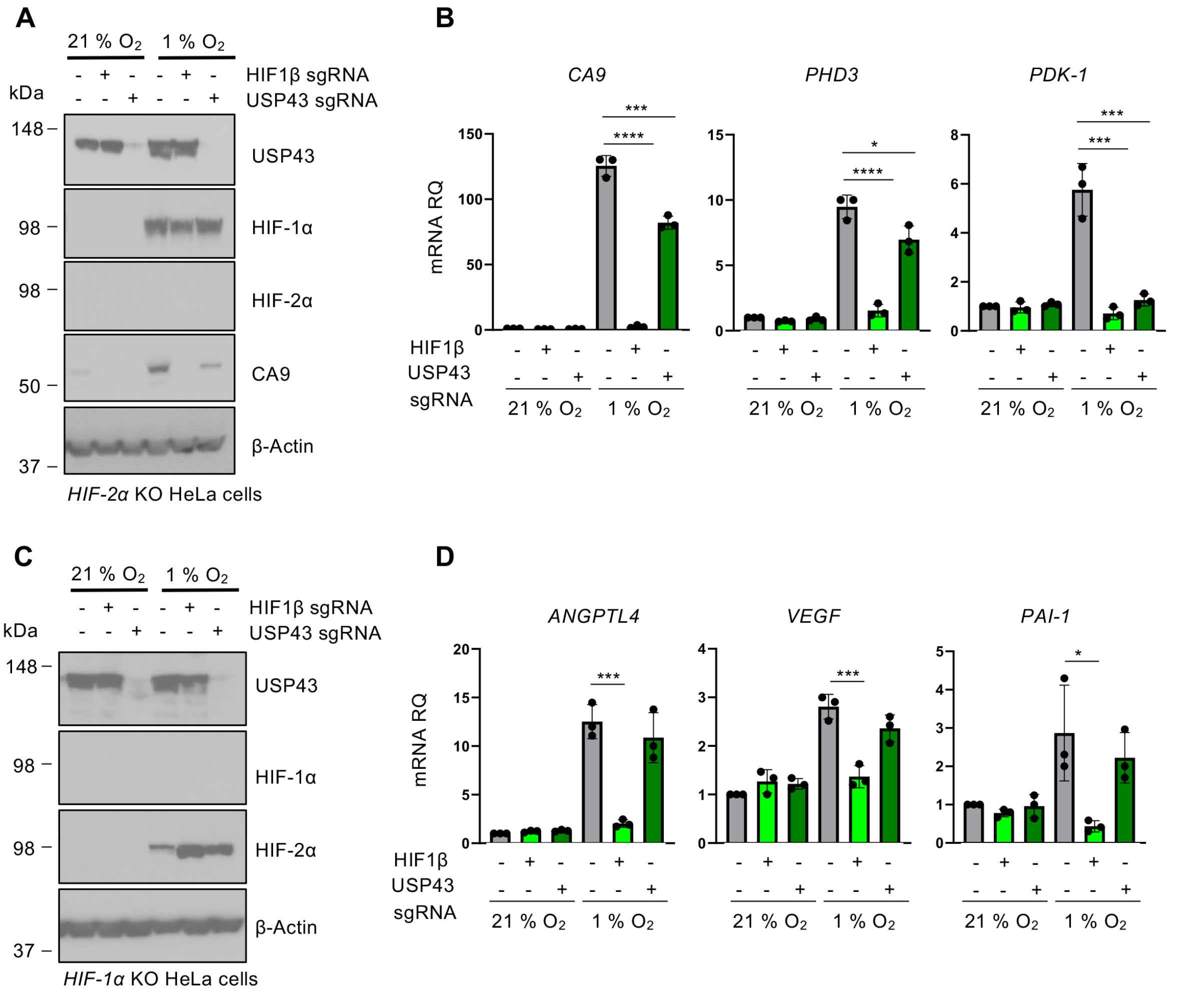

A, B) Clonal HIF-2α null HeLa cells were transduced with sgRNA targeting HIF1β or USP43. Cells were incubated in 21 % or 1 % oxygen for 16 h and analysed by immunoblot **(A)** or qPCR for selected HIF target genes **(B)**. n=3 biologically independent samples, mean ± sd. *CA9*: control vs. HIF1B KO ****P<0.0001, control vs. USP43 ***P=0.0001. *PHD3*: HIF1B KO ****P<0.0001, control vs. USP43 *P=0.0215. *PDK1*: control vs. HIF1B KO ***P=0.0001, control vs. USP43 ***P=0.0003. One-way ANOVA. **C, D)** Clonal HIF-1α null HeLa cells were transduced with sgRNA targeting HIF1β or USP43. Cells were incubated in 21 % or 1 % oxygen for 16 h and analysed by immunoblot **(C)** or qPCR for selected HIF target genes **(D)**. n=3 biologically independent samples, mean ± sd. *ANGPTL4*: control vs. HIF1B KO ***P=0.0007. *VEGF*: control vs. HIF1B KO ***P=0.0008. *PAI-1*: control vs. HIF1B KO *P=0.0192. One-way ANOVA.

Appendix Figure S3. Reconstituting USP43 deficiency restores HIF signalling.

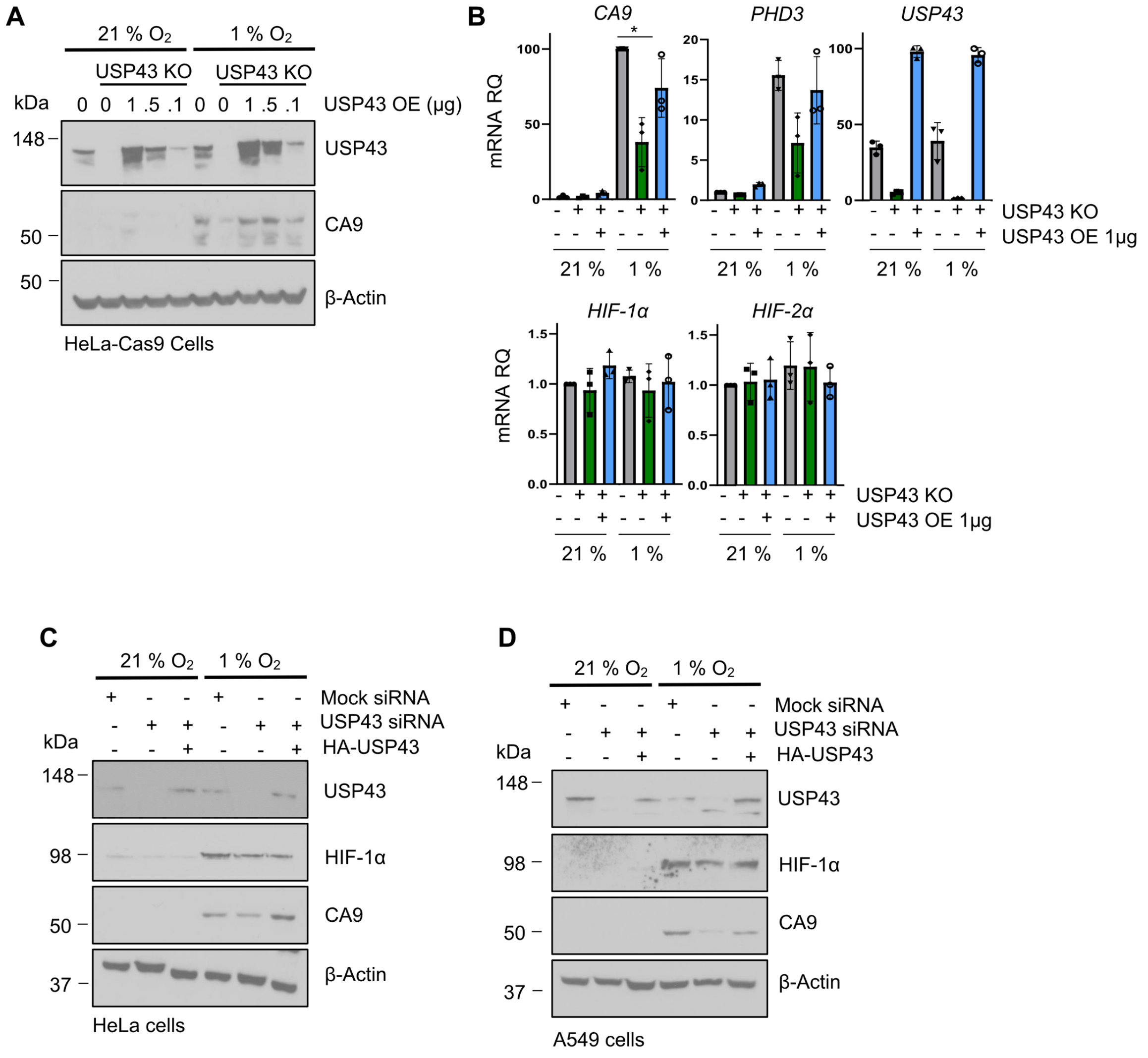

A, B) Control or USP43 clonal KO cells were reconstituted with 0, 1, 0.5, or 0.1 µg by transient transfection of USP43, incubated in 21 % or 1 % oxygen for 16 h, and analysed by immunoblot **(A)** or qPCR **(B)**. Representative of three biological replicates. n=3 biologically independent samples, mean ± sd. Control vs USP43 KO *P=0.049, one-way ANOVA. **C, D)** USP43 was depleted in HeLa cells using siRNA and compared to a mock siRNA control. siRNA transfected HeLa **(C)** or A549 **(D)** cells were then transfected with HA-USP43 (1 µg) to reconstitute USP43, and incubated in 21 % or 1 % oxygen for 16 h. Immunoblot representative of three biological replicates.

Appendix Figure S4. USP43 depletion does not alter H2B^{K120Ub} levels.

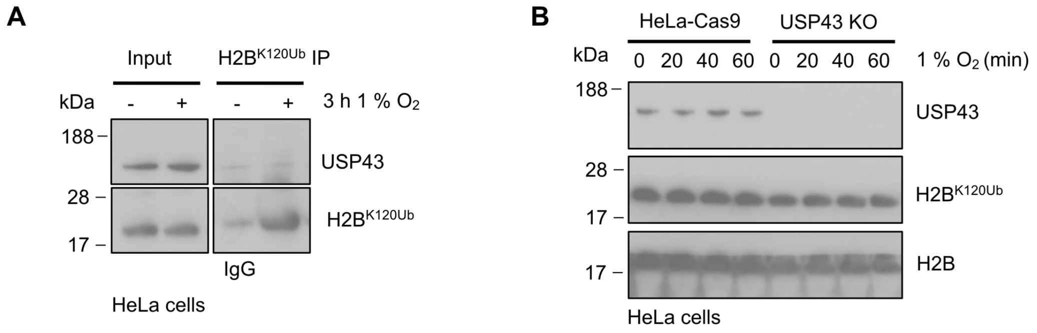

A) Endogenous H2B^{K120Ub} was immunoprecipitated in HeLa cells grown in 21 % or 1 % oxygen for 3 h. Samples were immunoblotted for USP43. Representative of 3 biological replicates). **B)** Immunoblot of H2B^{K120Ub} levels in control or USP43 null HeLa cells incubated in 1 % oxygen for 0-60 min. Representative of 3 biological replicates.

Appendix Figure S5

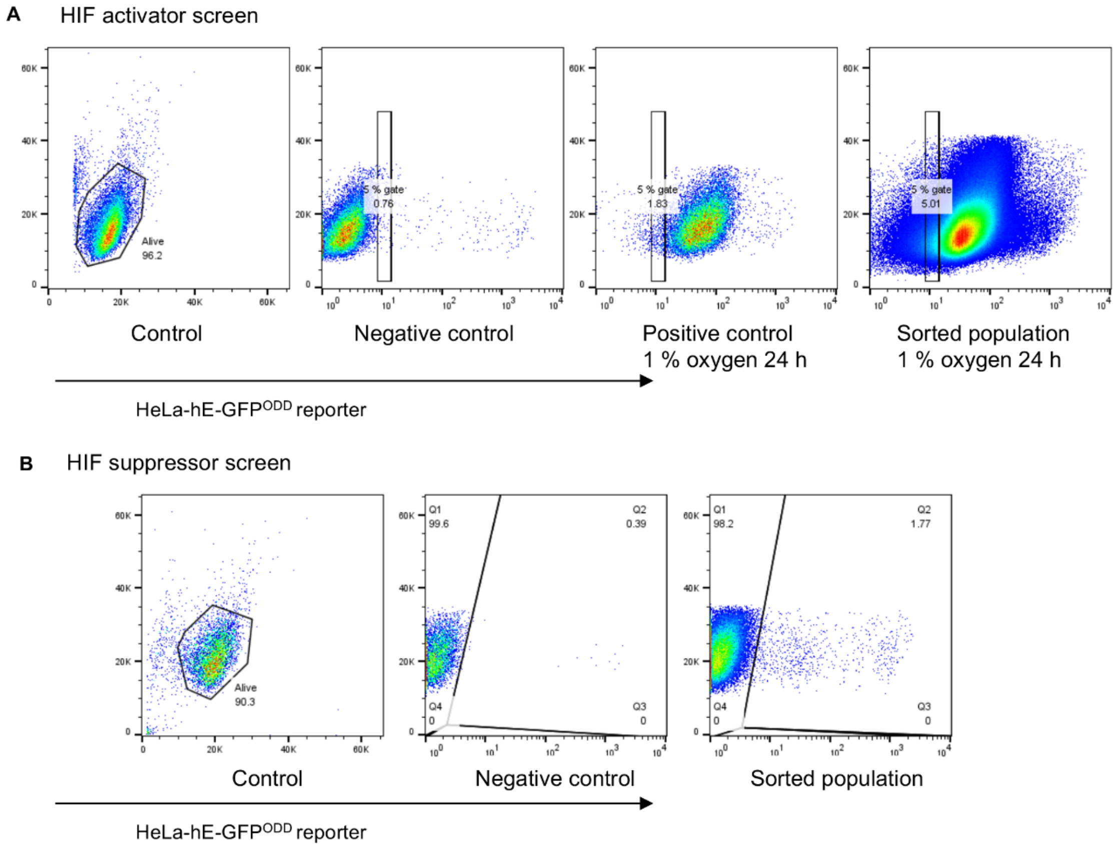

A, B) Representative gating strategy for the HeLa-HRE-GFP^{ODD} reporter screens. Gates for sorting the low GFP population in 1% oxygen **(A)** and GFP high population in 21% oxygen **(B)**.
