# Supplementary material for: Deubiquitinating enzyme mutagenesis screens identify a USP43-dependent HIF-1 transcriptional response
Source: EMBO J. 2024 Jul 15;43(17):8. doi: 10.1038/s44318-024-00166-6 (PMC11377827; doi:10.1038/s44318-024-00166-6)
Supplement: Supplementary file 5 — Source data Fig. 1 [file 44318_2024_166_MOESM5_ESM.zip › Figure 1/F1 B GFP high sort/Sort Report final 1150720.pdf]

Sort Report

System

Sort Start:	7/15/2020 12:51:03 PM	Server:	Utopex
Application:	BD FACS™ Sortware	Build:	1.2.0.106
Version:	1.2.0.142	Cytometer Model:	BD Influx System
ValComp:	7.5.1.3.16	Cytometer Serial #:	X646500J3001

Details

Data Source:	Cytometer	Sort Mode:	1.0 Drop Pure
Nozzle Diameter (µm):	0.00	Drop Envelope:	1.0 Drop
Sheath Pressure (PSI):	0.00	Sort Objective:	Purify
Sort Device:	2 Tube Holder - 2 Way Sort	Phase Mask:	16/16
Piezo Amplitude:	14.62	Extra Coincidence Bits:	4
Drop Delay:	42.6855	Drop Frequency (kHz):	42.76

Sort Details

Name	Population	Event Limit	Event Count	Sort Count	Sort Rate	Aborts	Abort Rate	Efficiency	Time (sec)
Left	GFP+	Unlimited	77,426,506	1,329,375	139	482,104	50	73.4%	9,501
Right	-	-	-	-	-	-	-	-	-
