# Supplementary material for: Deubiquitinating enzyme mutagenesis screens identify a USP43-dependent HIF-1 transcriptional response
Source: EMBO J. 2024 Jul 15;43(17):8. doi: 10.1038/s44318-024-00166-6 (PMC11377827; doi:10.1038/s44318-024-00166-6)
Supplement: Supplementary file 5 — Source data Fig. 1 [file 44318_2024_166_MOESM5_ESM.zip › Figure 1/F1 B GFP high sort/Sort Layout Updated HeLa DUBs + ctrl 150720.pdf]

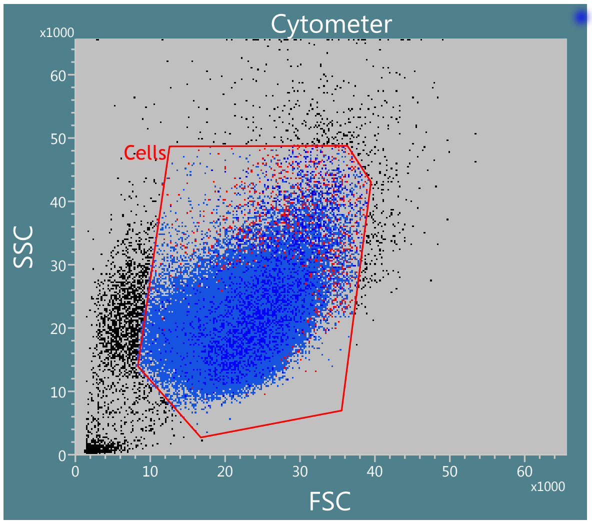

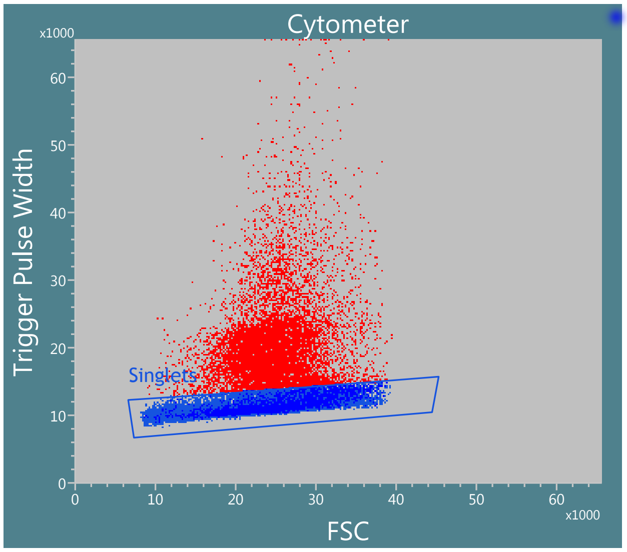

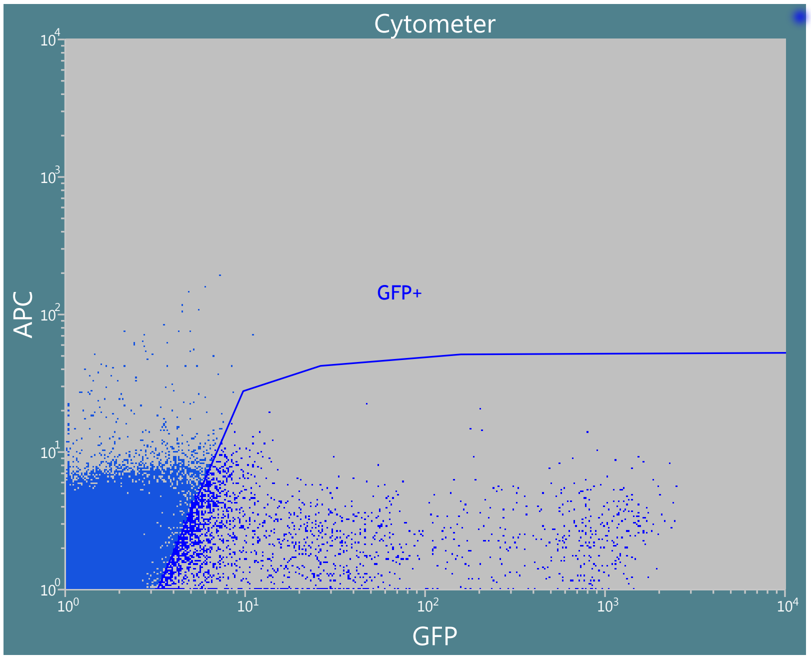

Statistics: Cytometer

Populations	Events	% Total	% Parent	GFP Mean	APC Mean
All Events	100,000	100.00%	####	6	3
Cells	96,958	96.96%	96.96%	6	2
Singlets	87,267	87.27%	90.00%	5	2
GFP+	2,504	2.50%	2.87%	130	3

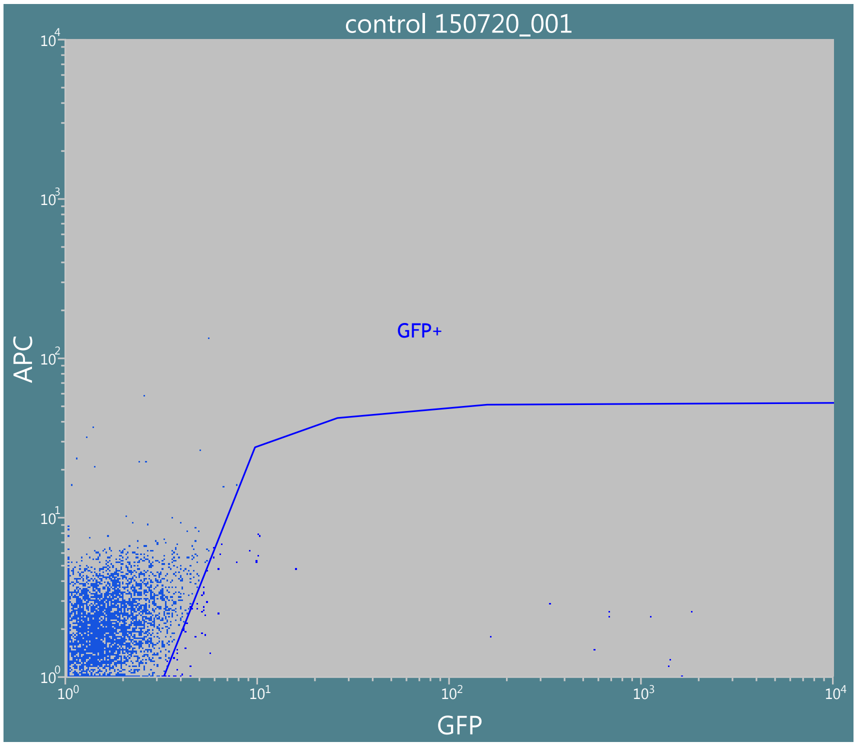

Statistics: control 150720_001

Populations	Events	% Total	% Parent	GFP Mean	APC Mean
All Events	6,183	100.00%	####	3	3
Cells	5,818	94.10%	94.10%	3	2
Singlets	5,503	89.00%	94.59%	3	2
GFP+	71	1.15%	1.29%	141	3

Statistics: control 150720_001

Populations	Events	% Total	% Parent	GFP Mean	APC Mean
All Events	6,183	100.00%	####	3	3
Cells	5,818	94.10%	94.10%	3	2
Singlets	5,503	89.00%	94.59%	3	2
GFP+	71	1.15%	1.29%	141	3
