# Supplementary figures and images for: Deubiquitinating enzyme mutagenesis screens identify a USP43-dependent HIF-1 transcriptional response
Source: EMBO J. 2024 Jul 15;43(17):8. doi: 10.1038/s44318-024-00166-6 (PMC11377827; doi:10.1038/s44318-024-00166-6)

## Slide 1
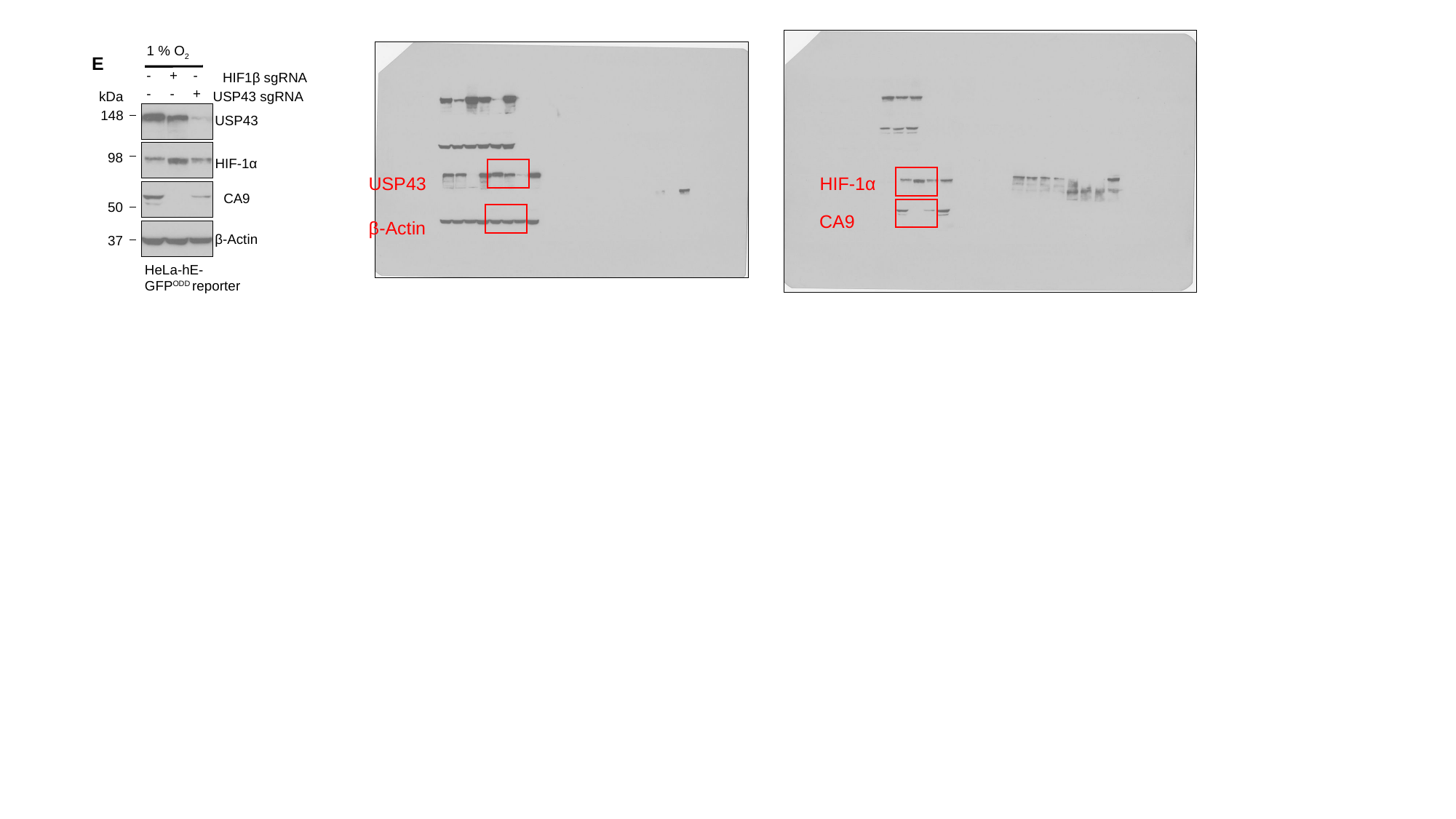

1 % O2
E
-
+
-
HIF1β sgRNA
-
-
+
kDa
USP43 sgRNA
148
USP43
98
HIF-1α
HIF-1α
USP43
CA9
50
CA9
β-Actin
β-Actin
37
HeLa-hE-GFPODD reporter

Supplement: Supplementary file 5 — Source data Fig. 1 [file 44318_2024_166_MOESM5_ESM.zip › Figure 1/F1 E WB.pptx]

## Slide 1
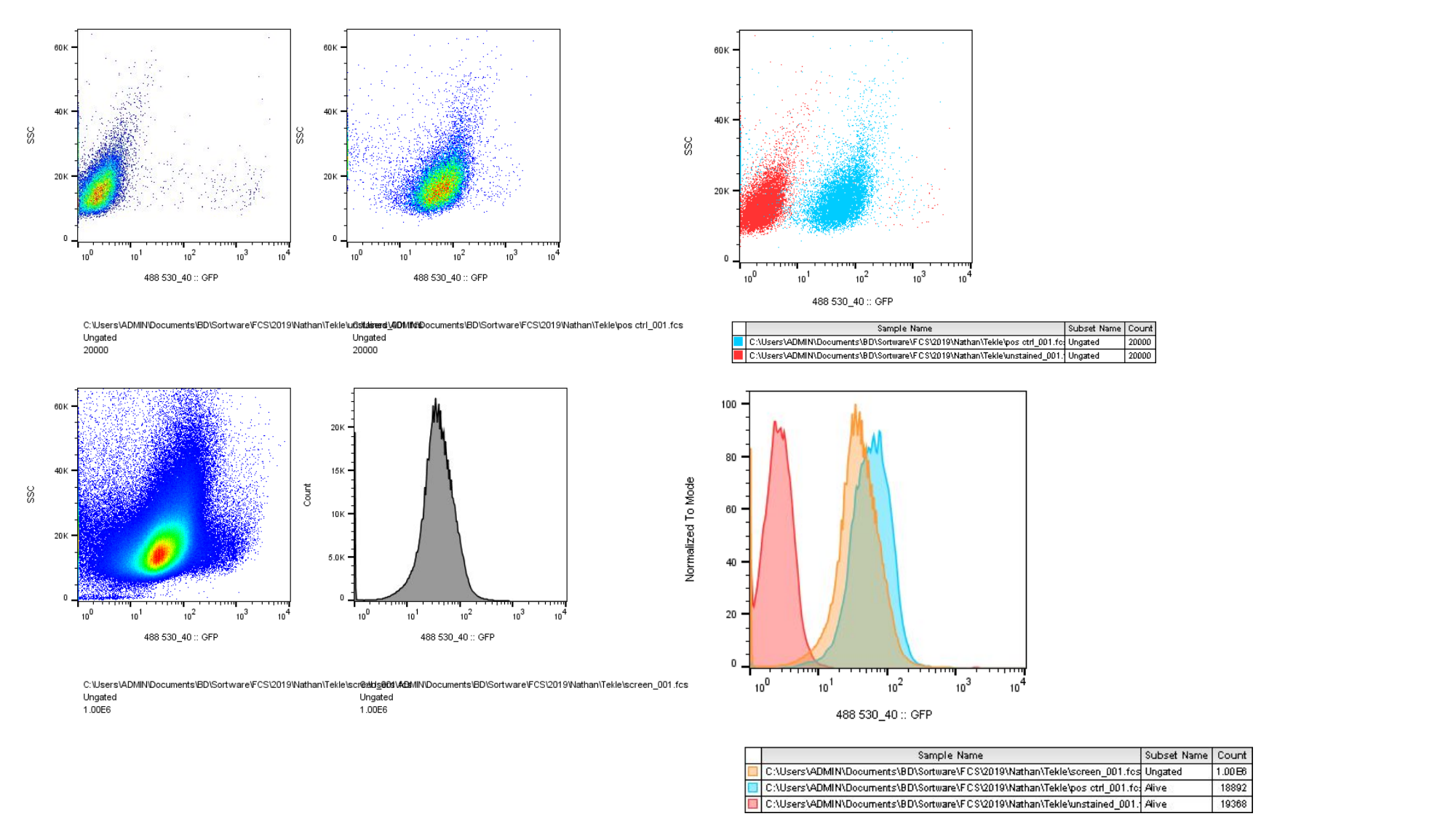

Supplement: Supplementary file 5 — Source data Fig. 1 [file 44318_2024_166_MOESM5_ESM.zip › Figure 1/F1 C GFP low sort/2019 07 25 DUBs sort 1.pptx]

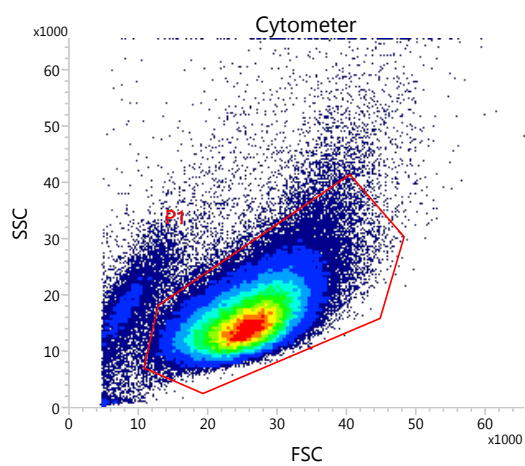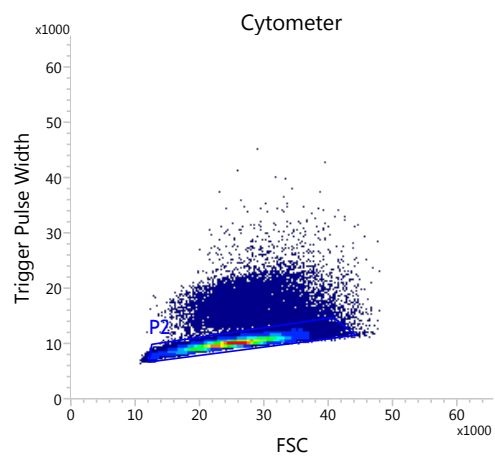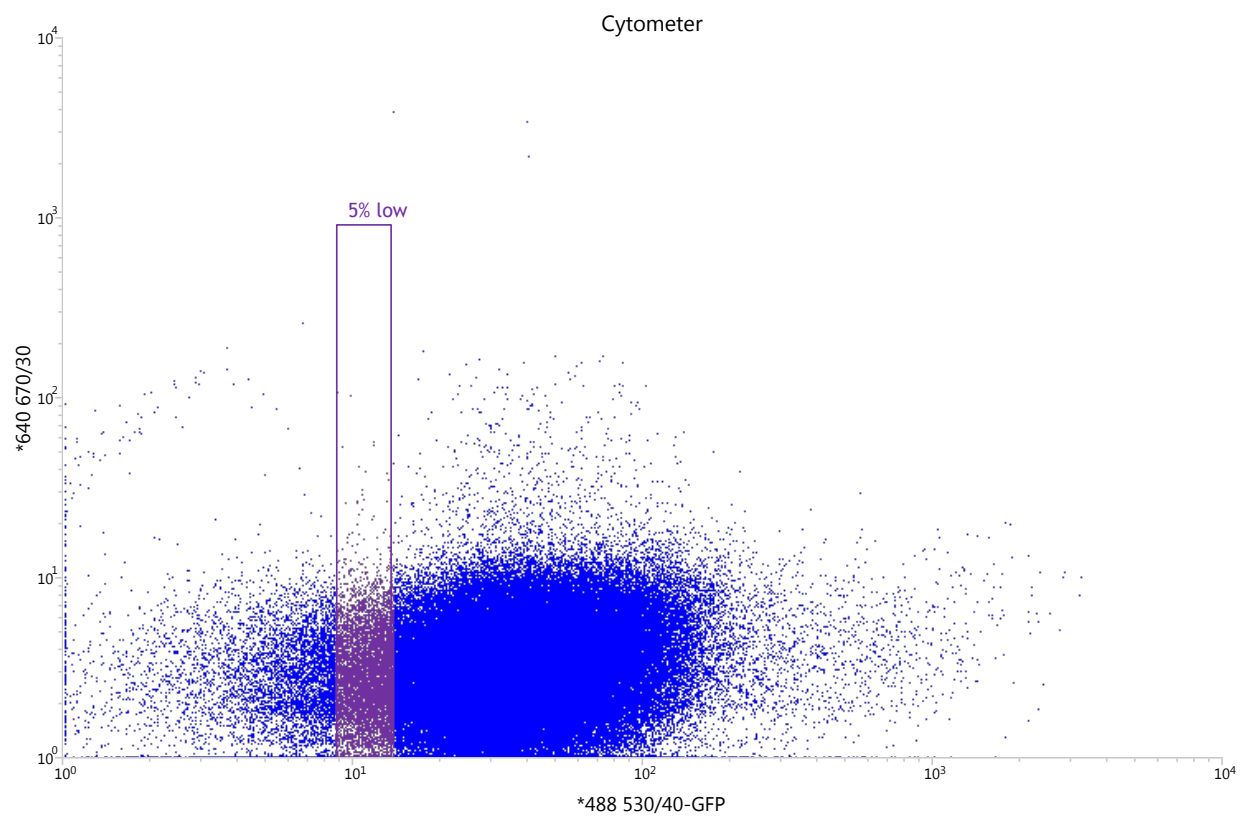

| Statistics: Cytometer |         |         |          |          |          |  |
|-----------------------|---------|---------|----------|----------|----------|--|
| Populations           | Events  | % Total | % Parent | FSC Mean | SSC Mean |  |
| All Events            | 200,000 | 100.00% | ####     | 25,173   | 16,402   |  |
| P1                    | 188,639 | 94.32%  | 94.32%   | 25,761   | 15,940   |  |
| P2                    | 175,792 | 87.90%  | 93.19%   | 25,550   | 15,691   |  |
| 5% low                | 8,603   | 4.30%   | 4.89%    | 22,539   | 13,312   |  |

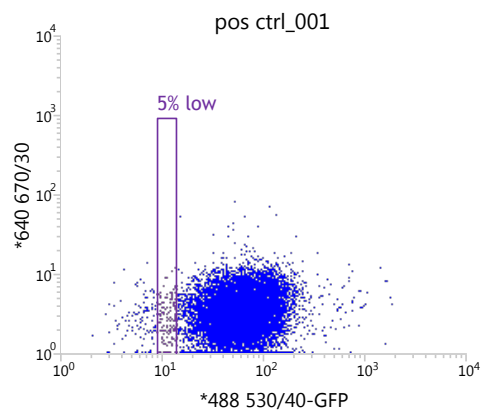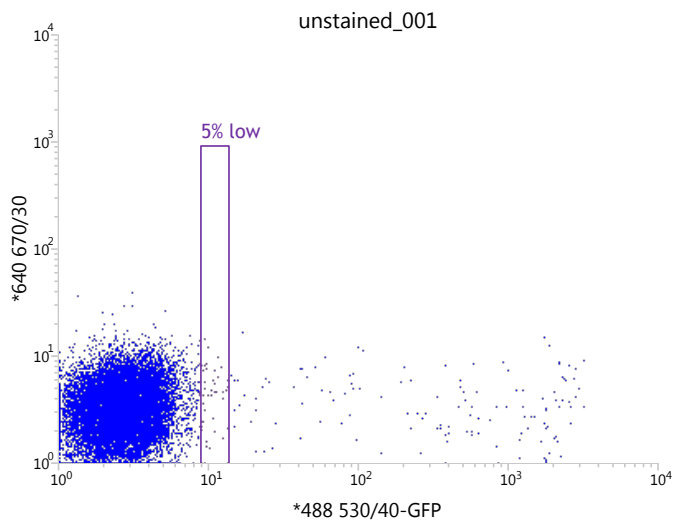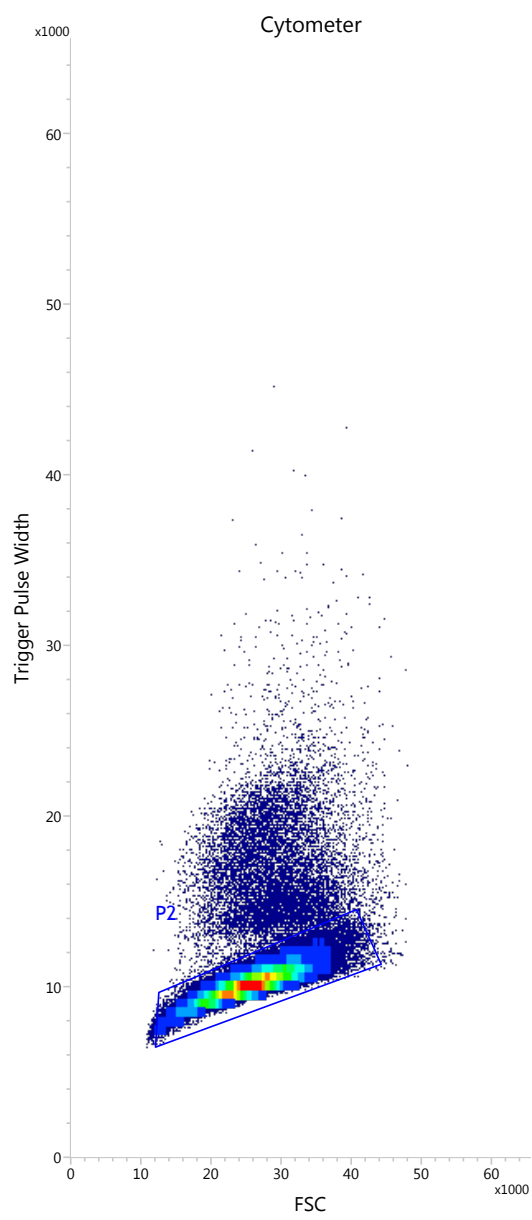

Supplement: Supplementary file 5 — Source data Fig. 1 [file 44318_2024_166_MOESM5_ESM.zip › Figure 1/F1 C GFP low sort/screen sort layout.pdf]

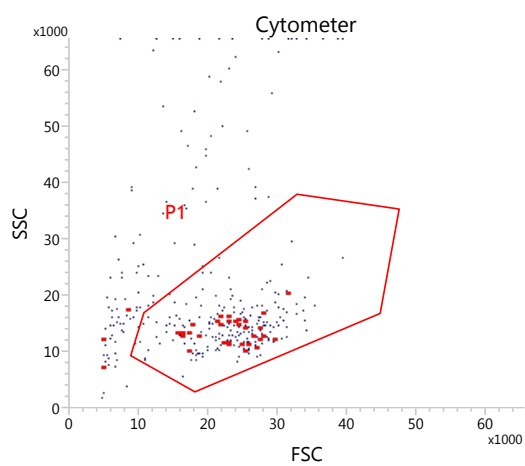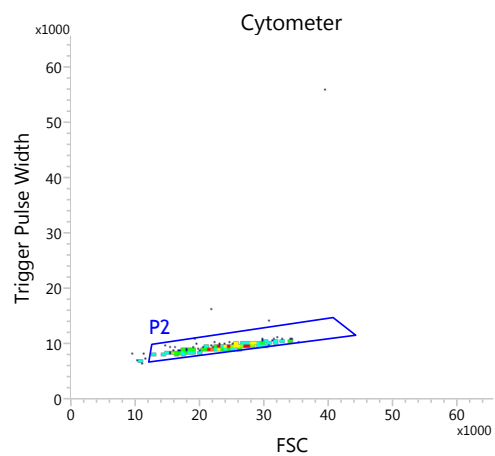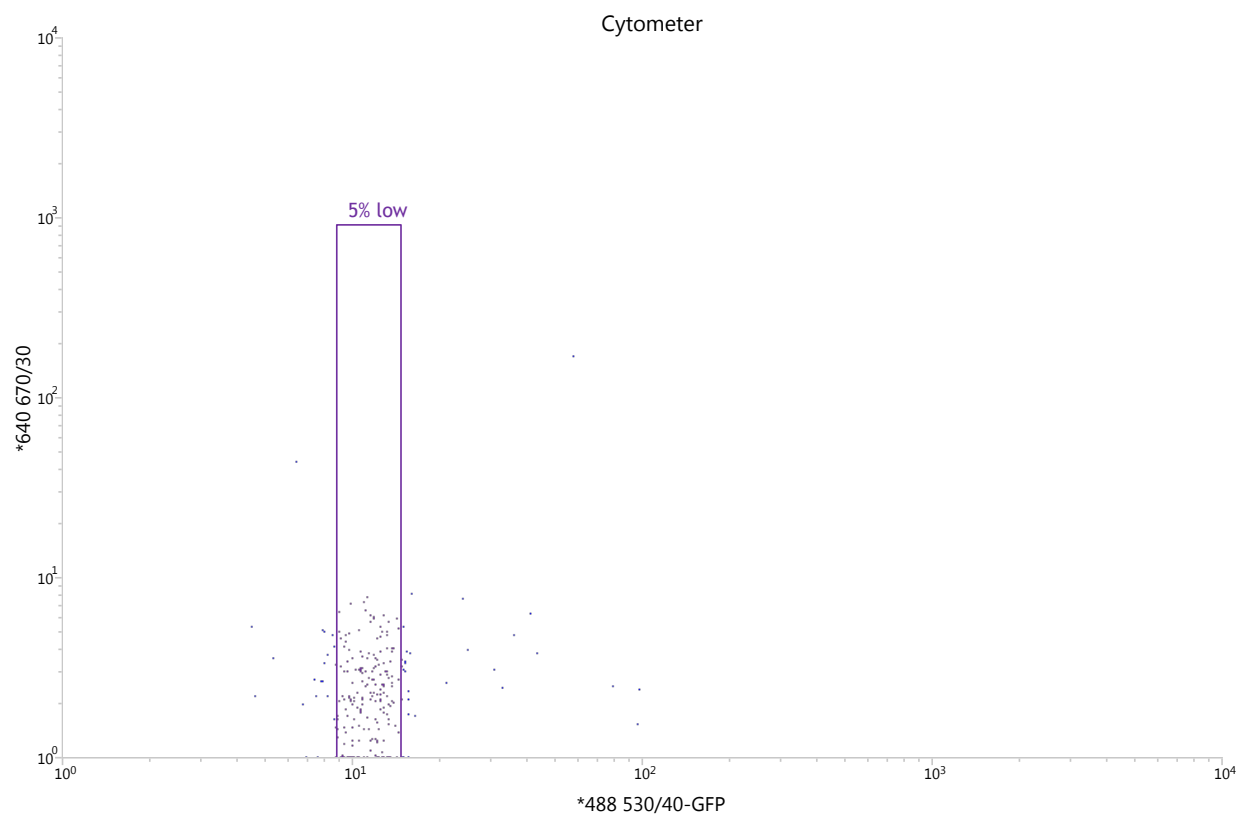

| Statistics: Cytometer |        |         |          |          |          |  |
|-----------------------|--------|---------|----------|----------|----------|--|
| Populations           | Events | % Total | % Parent | FSC Mean | SSC Mean |  |
| All Events            | 356    | 100.00% | ####     | 20,286   | 19,549   |  |
| P1                    | 245    | 68.82%  | 68.82%   | 23,289   | 14,154   |  |
| P2                    | 235    | 66.01%  | 95.92%   | 23,577   | 14,081   |  |
| 5% low                | 189    | 53.09%  | 80.43%   | 23,350   | 13,818   |  |

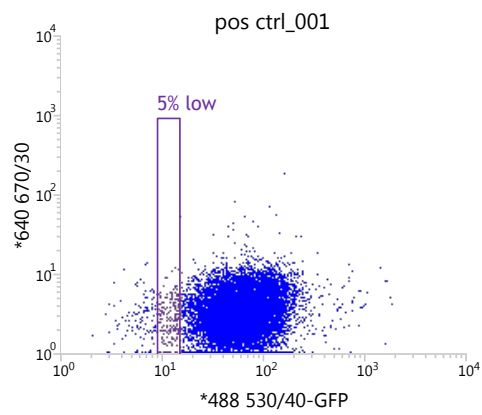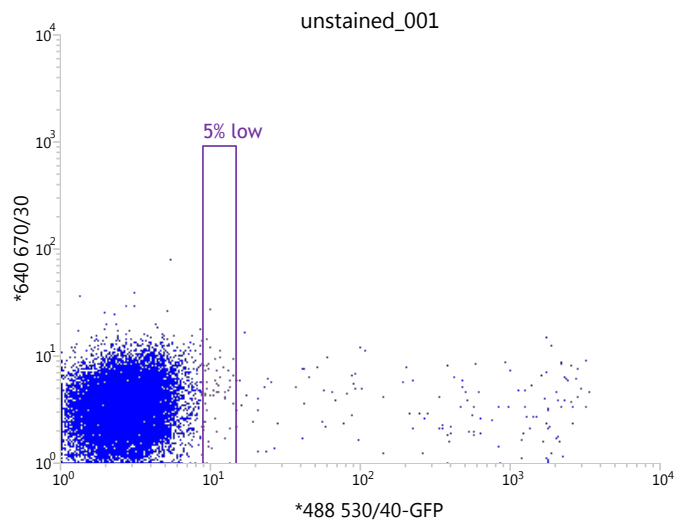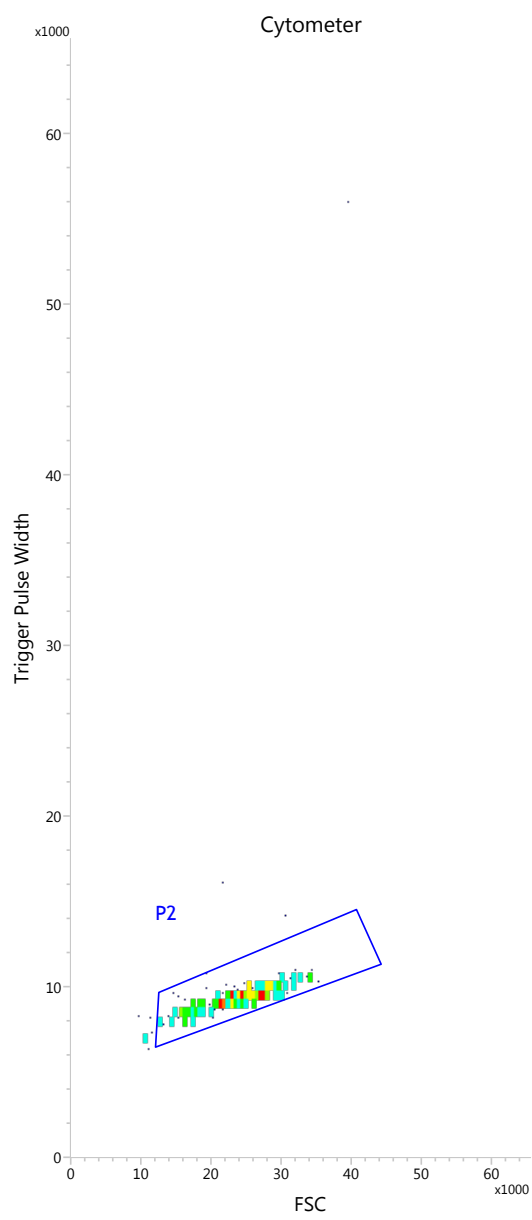

Supplement: Supplementary file 5 — Source data Fig. 1 [file 44318_2024_166_MOESM5_ESM.zip › Figure 1/F1 C GFP low sort/Purity check.pdf]

## Slide 1
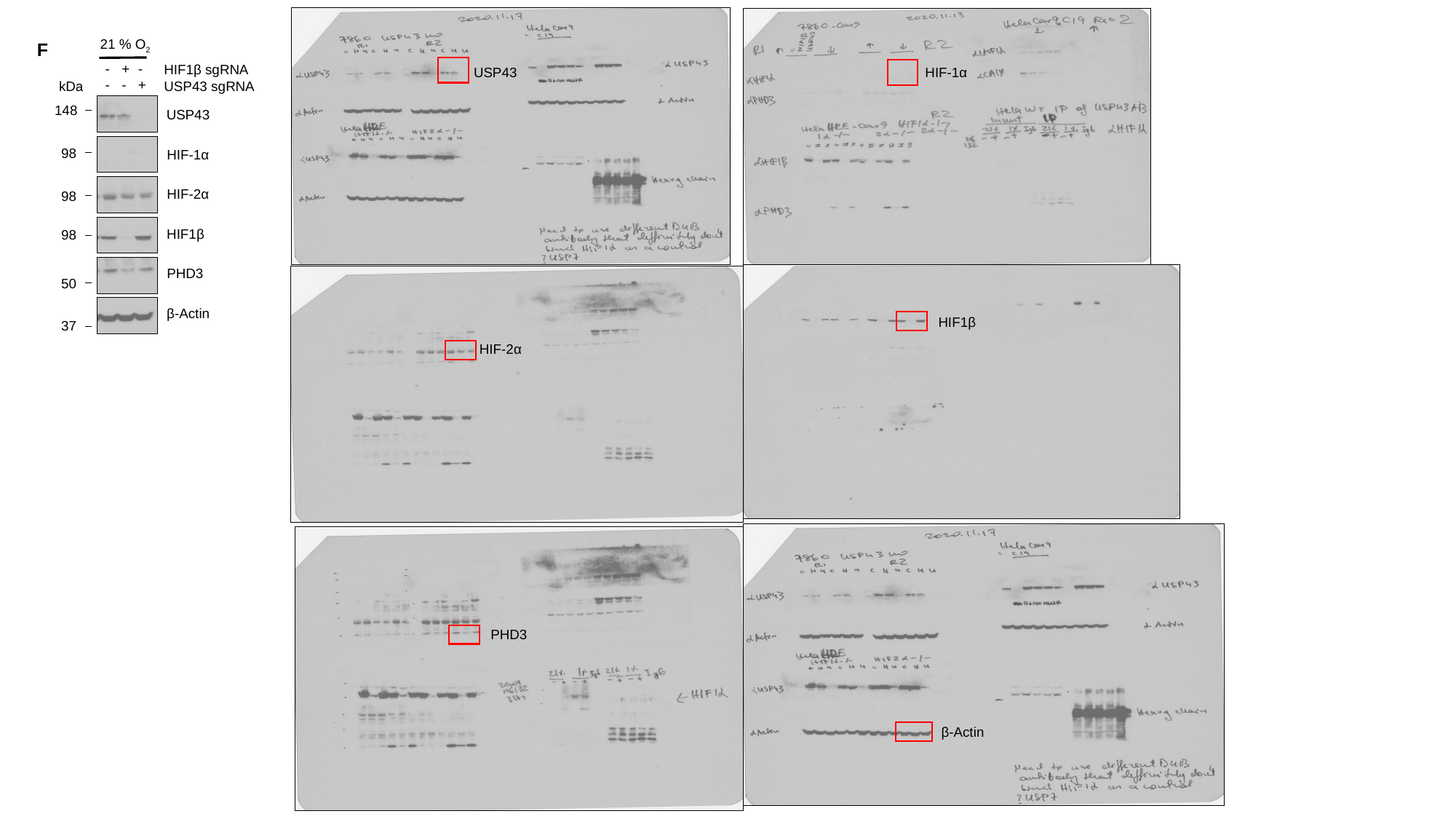

21 % O2
-
+
-
-
-
+
F
HIF1β sgRNA
USP43
HIF-1α
kDa
USP43 sgRNA
148
USP43
98
HIF-1α
HIF-2α
98
HIF1β
98
PHD3
50
β-Actin
HIF1β
37
HIF-2α
PHD3
β-Actin

Supplement: Supplementary file 7 — Source data Fig. 3 [file 44318_2024_166_MOESM7_ESM.zip › Figure 3/F3 F 7860 WB.pptx]

## Slide 1
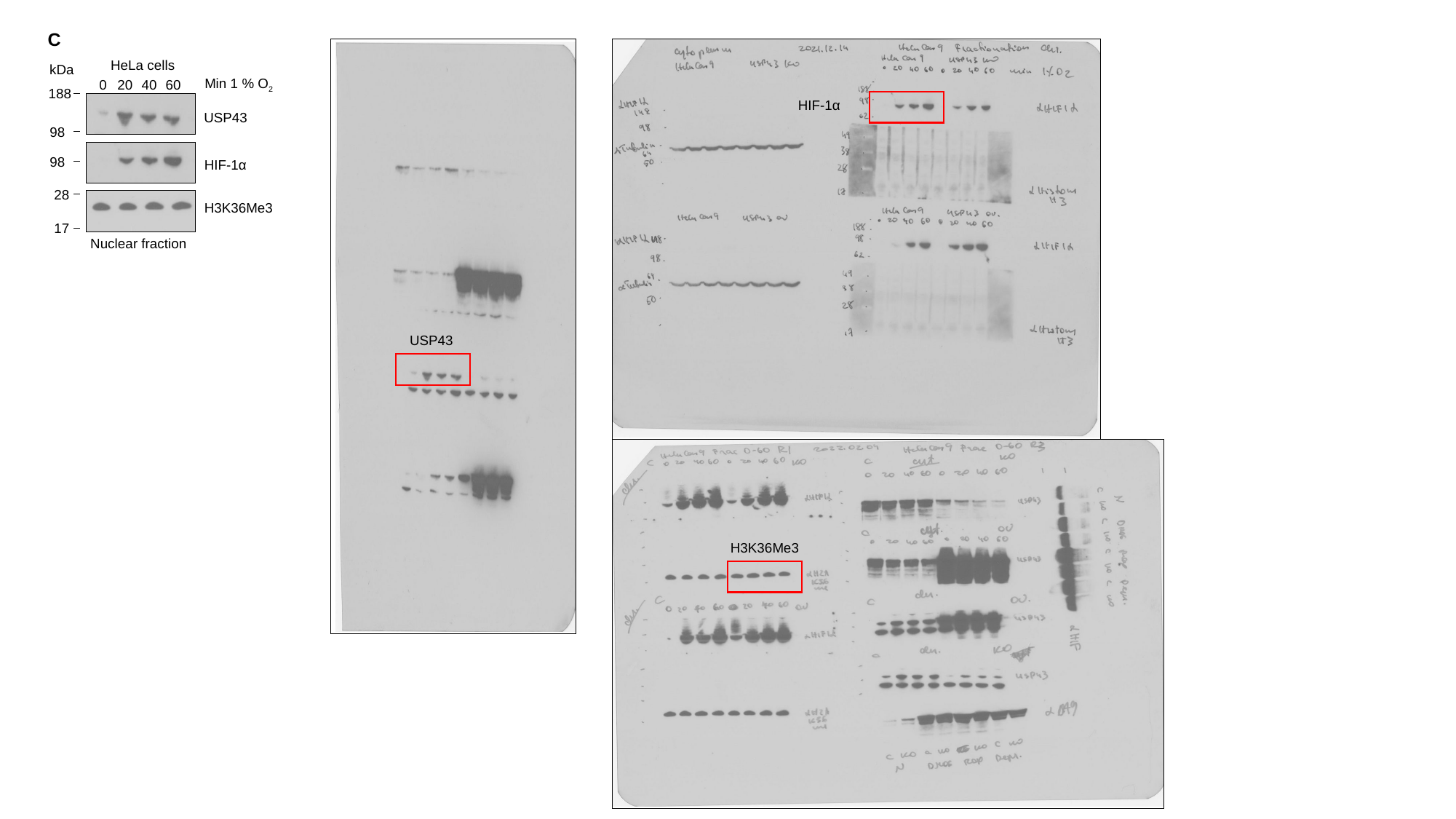

C
HeLa cells
kDa
Min 1 % O2
0
20
40
60
188
HIF-1α
USP43
98
98
HIF-1α
28
H3K36Me3
17
Nuclear fraction
USP43
H3K36Me3

Supplement: Supplementary file 9 — Source data Fig. 5 [file 44318_2024_166_MOESM9_ESM.zip › Figure 5/F5 C fract WB.pptx]
