# Supplementary material for: Deubiquitinating enzyme mutagenesis screens identify a USP43-dependent HIF-1 transcriptional response
Source: EMBO J. 2024 Jul 15;43(17):8. doi: 10.1038/s44318-024-00166-6 (PMC11377827; doi:10.1038/s44318-024-00166-6)
Supplement: Supplementary file 6 — Source data Fig. 2 [file 44318_2024_166_MOESM6_ESM.zip › Figure 2/F2 D WB.pptx]

## Slide 1
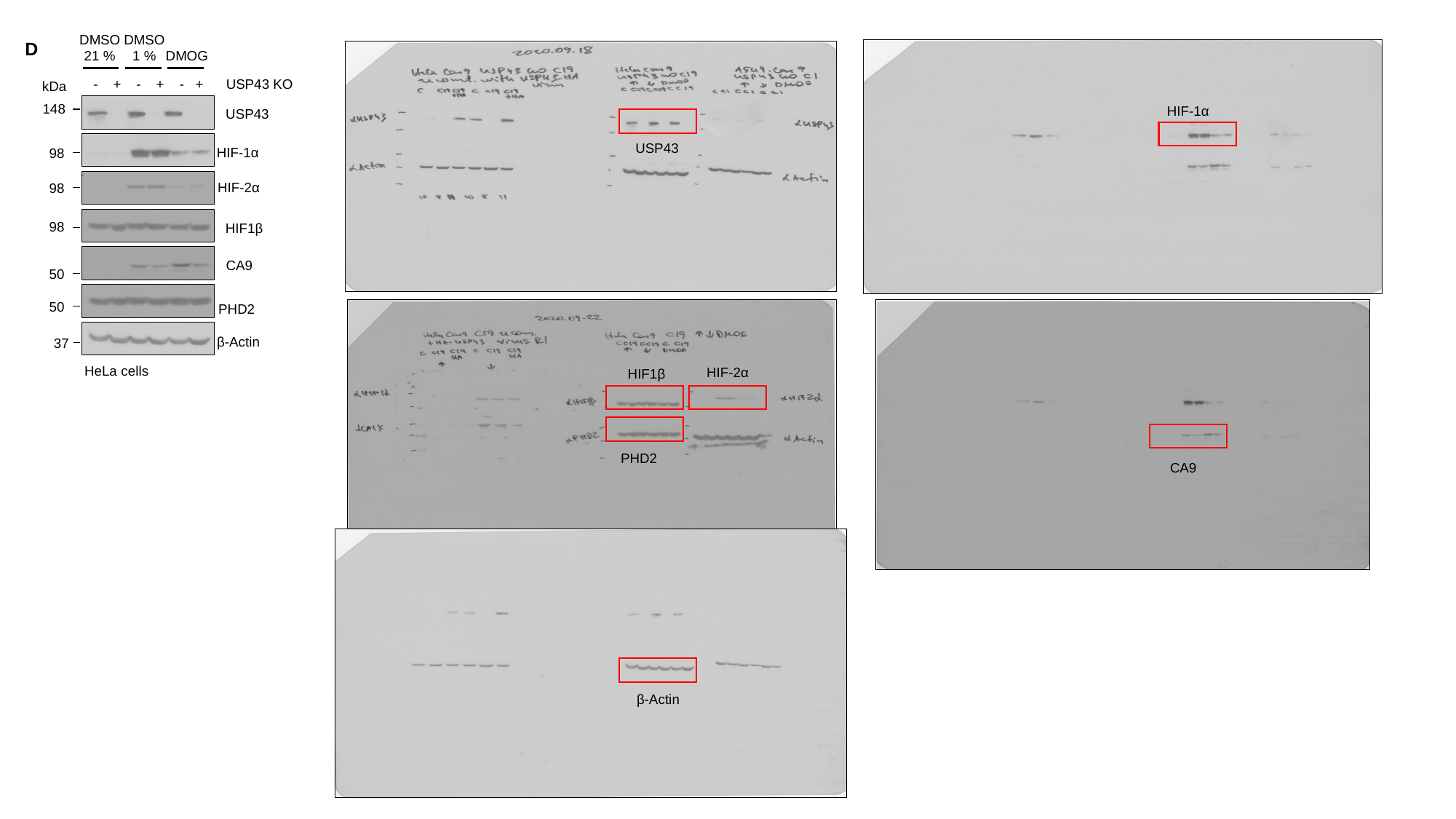

DMSO 21 %
DMSO 1 %
D
DMOG
- + - + - +
USP43 KO
kDa
148
HIF-1α
USP43
USP43
HIF-1α
98
HIF-2α
98
98
HIF1β
CA9
50
50
PHD2
β-Actin
37
HeLa cells
HIF-2α
HIF1β
PHD2
CA9
β-Actin
