# Supplementary material for: Deubiquitinating enzyme mutagenesis screens identify a USP43-dependent HIF-1 transcriptional response
Source: EMBO J. 2024 Jul 15;43(17):8. doi: 10.1038/s44318-024-00166-6 (PMC11377827; doi:10.1038/s44318-024-00166-6)
Supplement: Supplementary file 6 — Source data Fig. 2 [file 44318_2024_166_MOESM6_ESM.zip › Figure 2/F2 A WB HeLa A549 MCF7.pptx]

## Slide 1
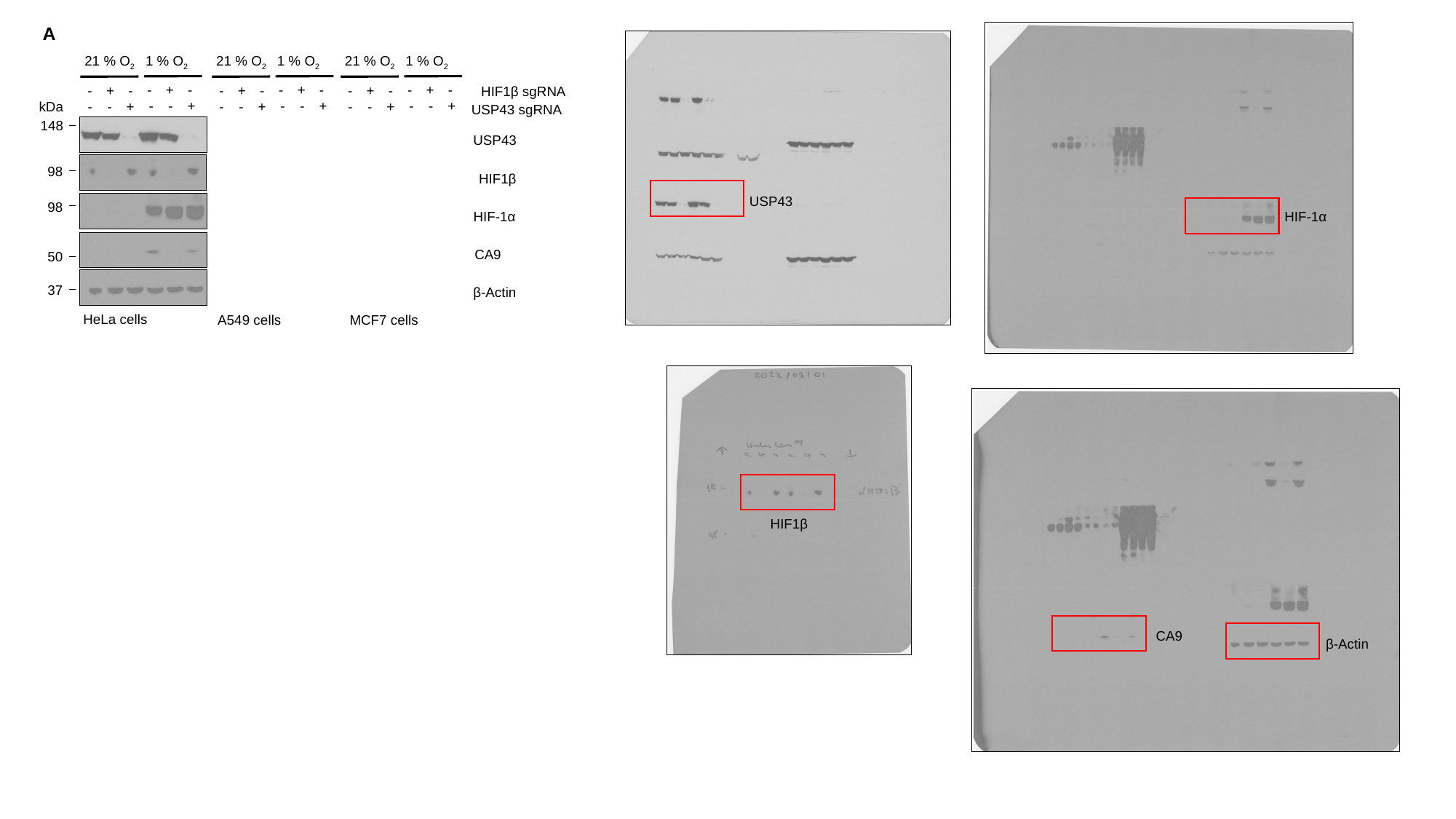

A
1 % O2
21 % O2
-
+
-
-
+
-
-
-
+
-
-
+
1 % O2
21 % O2
-
+
-
-
+
-
-
-
+
-
-
+
1 % O2
21 % O2
-
+
-
-
+
-
-
-
+
-
-
+
HIF1β sgRNA
kDa
USP43 sgRNA
148
USP43
98
HIF1β
USP43
98
HIF-1α
HIF-1α
CA9
50
37
β-Actin
HeLa cells
A549 cells
MCF7 cells
HIF1β
CA9
β-Actin

## Slide 2
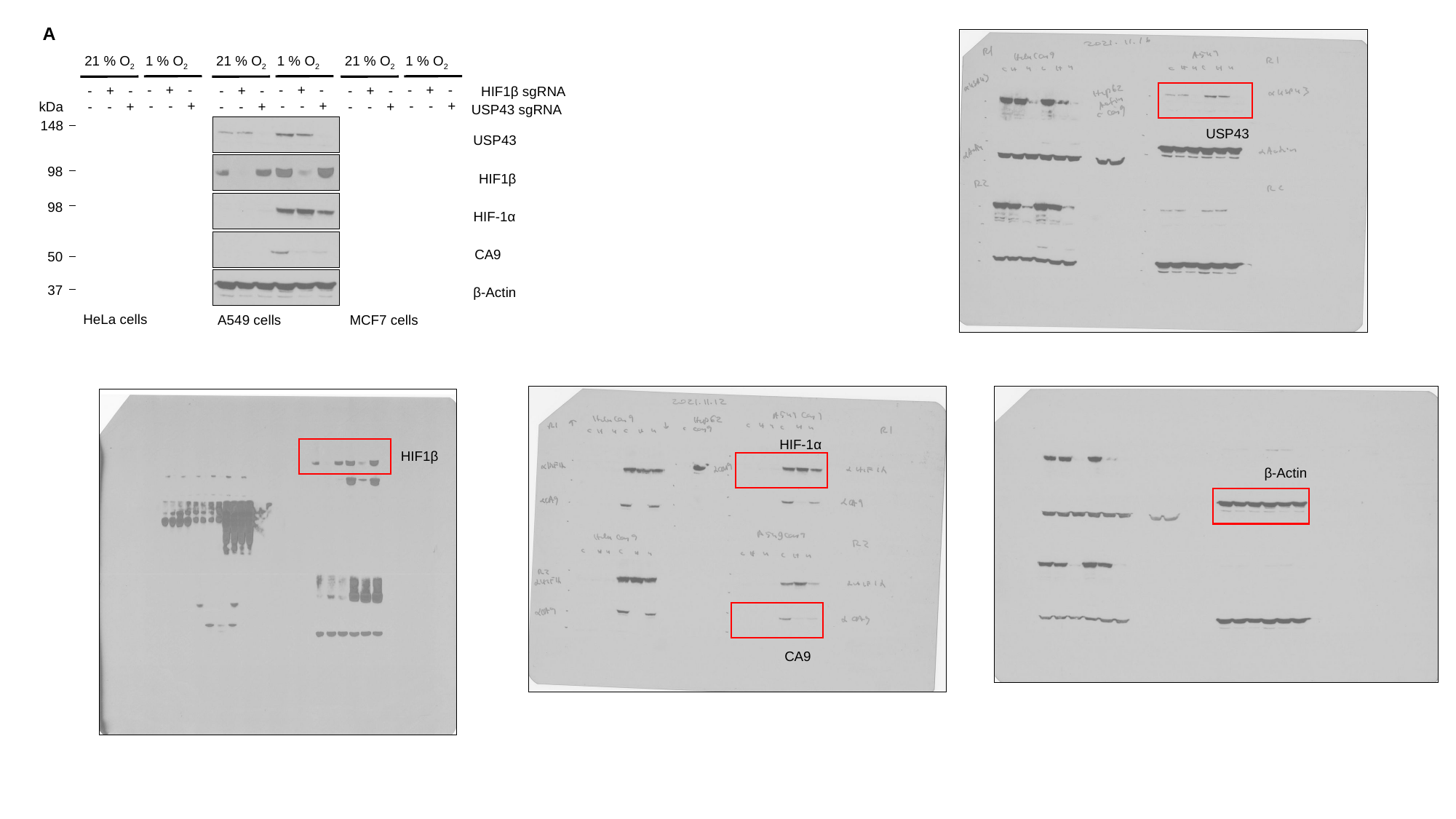

A
1 % O2
21 % O2
-
+
-
-
+
-
-
-
+
-
-
+
1 % O2
21 % O2
-
+
-
-
+
-
-
-
+
-
-
+
1 % O2
21 % O2
-
+
-
-
+
-
-
-
+
-
-
+
HIF1β sgRNA
kDa
USP43 sgRNA
148
USP43
USP43
98
HIF1β
98
HIF-1α
CA9
50
37
β-Actin
HeLa cells
A549 cells
MCF7 cells
HIF-1α
HIF1β
β-Actin
CA9

## Slide 3
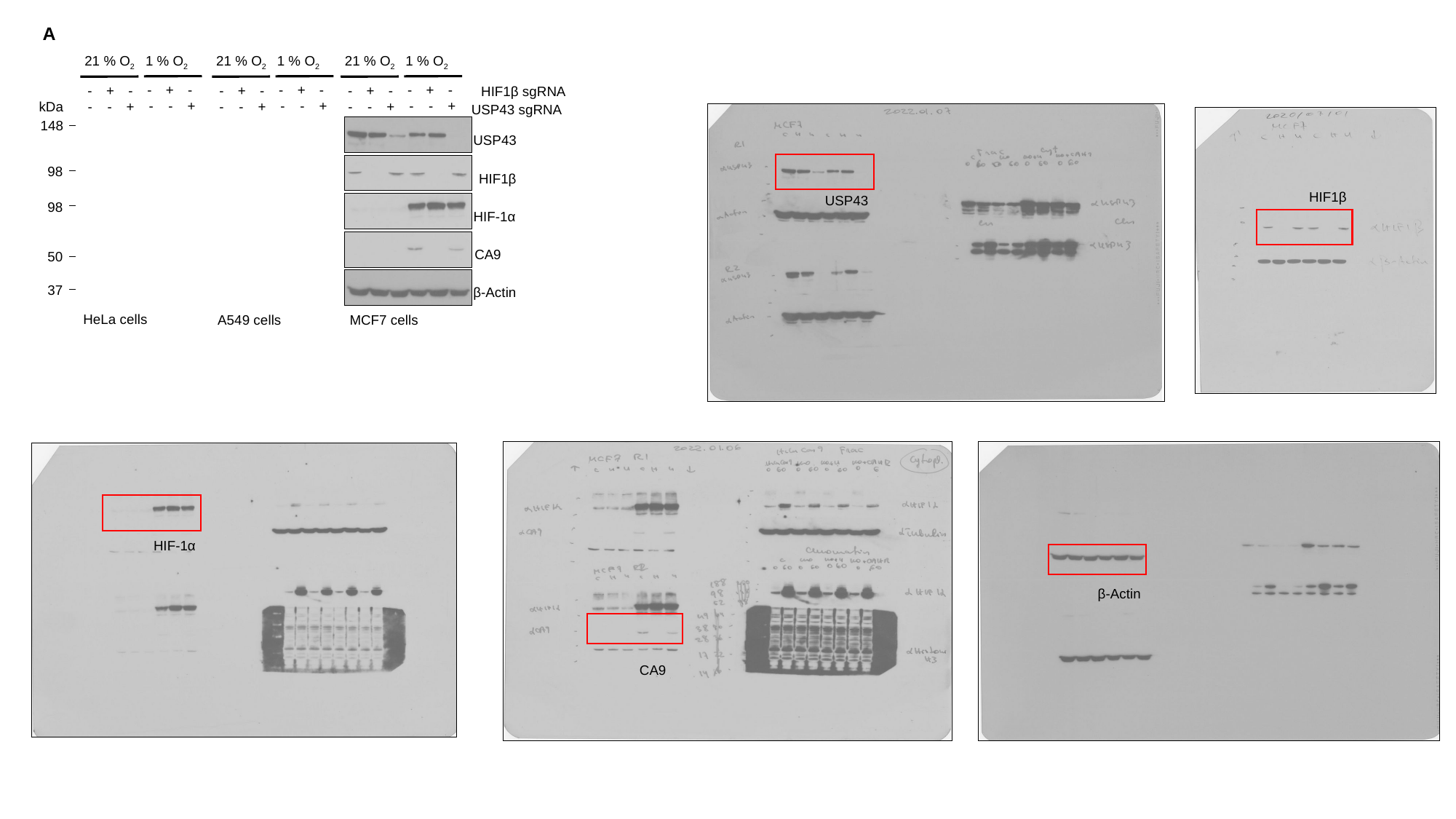

A
1 % O2
21 % O2
-
+
-
-
+
-
-
-
+
-
-
+
1 % O2
21 % O2
-
+
-
-
+
-
-
-
+
-
-
+
1 % O2
21 % O2
-
+
-
-
+
-
-
-
+
-
-
+
HIF1β sgRNA
kDa
USP43 sgRNA
148
USP43
98
HIF1β
HIF1β
USP43
98
HIF-1α
CA9
50
37
β-Actin
HeLa cells
A549 cells
MCF7 cells
HIF-1α
β-Actin
CA9
