# Supplementary material for: Deubiquitinating enzyme mutagenesis screens identify a USP43-dependent HIF-1 transcriptional response
Source: EMBO J. 2024 Jul 15;43(17):8. doi: 10.1038/s44318-024-00166-6 (PMC11377827; doi:10.1038/s44318-024-00166-6)
Supplement: Supplementary file 6 — Source data Fig. 2 [file 44318_2024_166_MOESM6_ESM.zip › Figure 2/F2 H A549 Rox.pptx]

## Slide 1
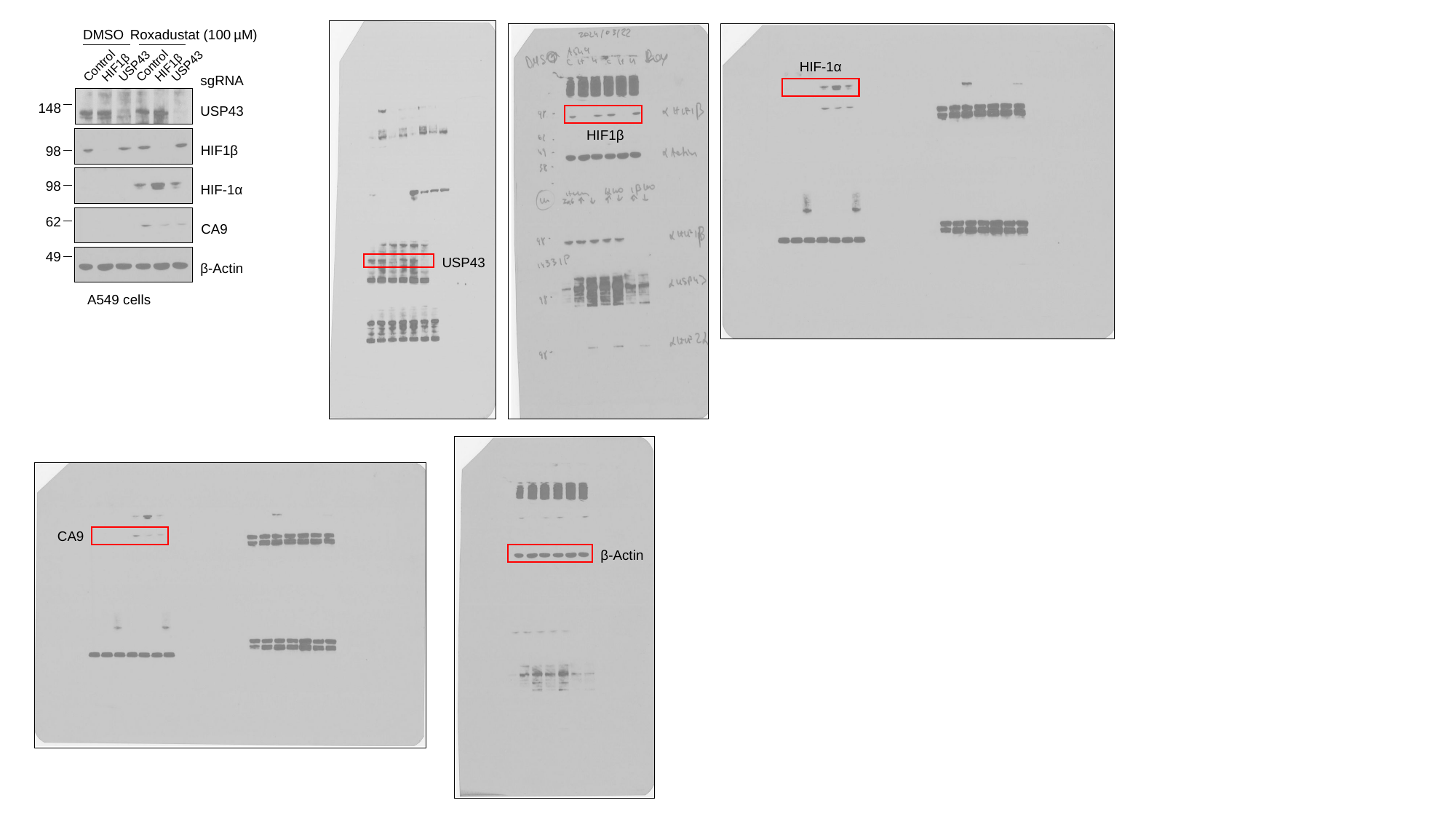

DMSO
Roxadustat (100 µM)
HIF-1α
Control
Control
USP43
USP43
HIF1β
HIF1β
sgRNA
148
USP43
HIF1β
HIF1β
98
98
HIF-1α
62
CA9
49
USP43
β-Actin
A549 cells
CA9
β-Actin
