# Supplementary material for: Deubiquitinating enzyme mutagenesis screens identify a USP43-dependent HIF-1 transcriptional response
Source: EMBO J. 2024 Jul 15;43(17):8. doi: 10.1038/s44318-024-00166-6 (PMC11377827; doi:10.1038/s44318-024-00166-6)
Supplement: Supplementary file 7 — Source data Fig. 3 [file 44318_2024_166_MOESM7_ESM.zip › Figure 3/F3 C WB RCC4.pptx]

## Slide 1
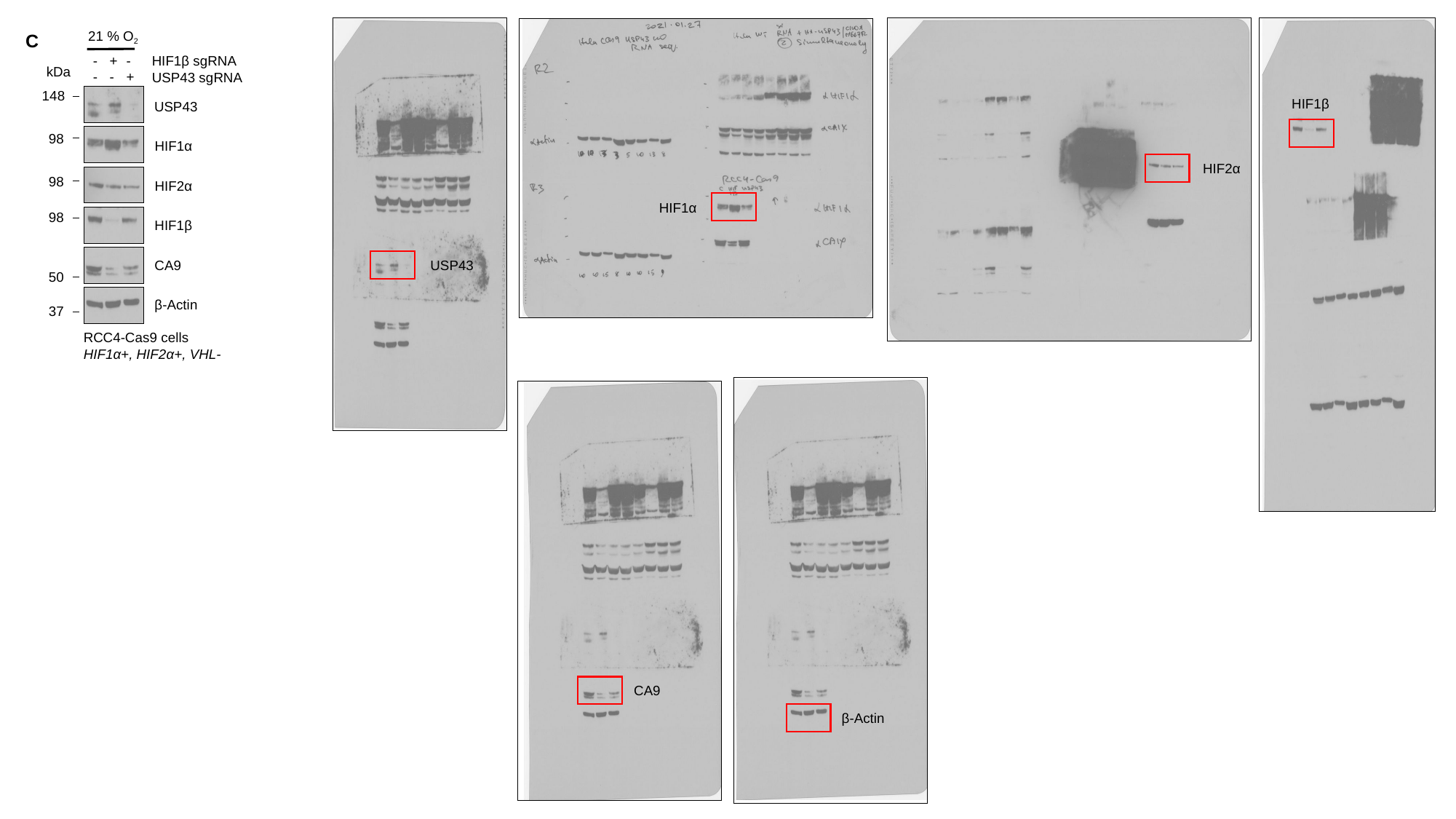

21 % O2
-
+
-
-
-
+
C
HIF1β sgRNA
kDa
USP43 sgRNA
148
HIF1β
USP43
98
HIF1α
HIF2α
98
HIF2α
HIF1α
98
HIF1β
CA9
USP43
50
β-Actin
37
RCC4-Cas9 cells
HIF1α+, HIF2α+, VHL-
CA9
β-Actin
