# Supplementary material for: Deubiquitinating enzyme mutagenesis screens identify a USP43-dependent HIF-1 transcriptional response
Source: EMBO J. 2024 Jul 15;43(17):8. doi: 10.1038/s44318-024-00166-6 (PMC11377827; doi:10.1038/s44318-024-00166-6)
Supplement: Supplementary file 8 — Source data Fig. 4 [file 44318_2024_166_MOESM8_ESM.zip › Figure 4/F4 A and B USP43 HIF1A IP.pptx]

## Slide 1
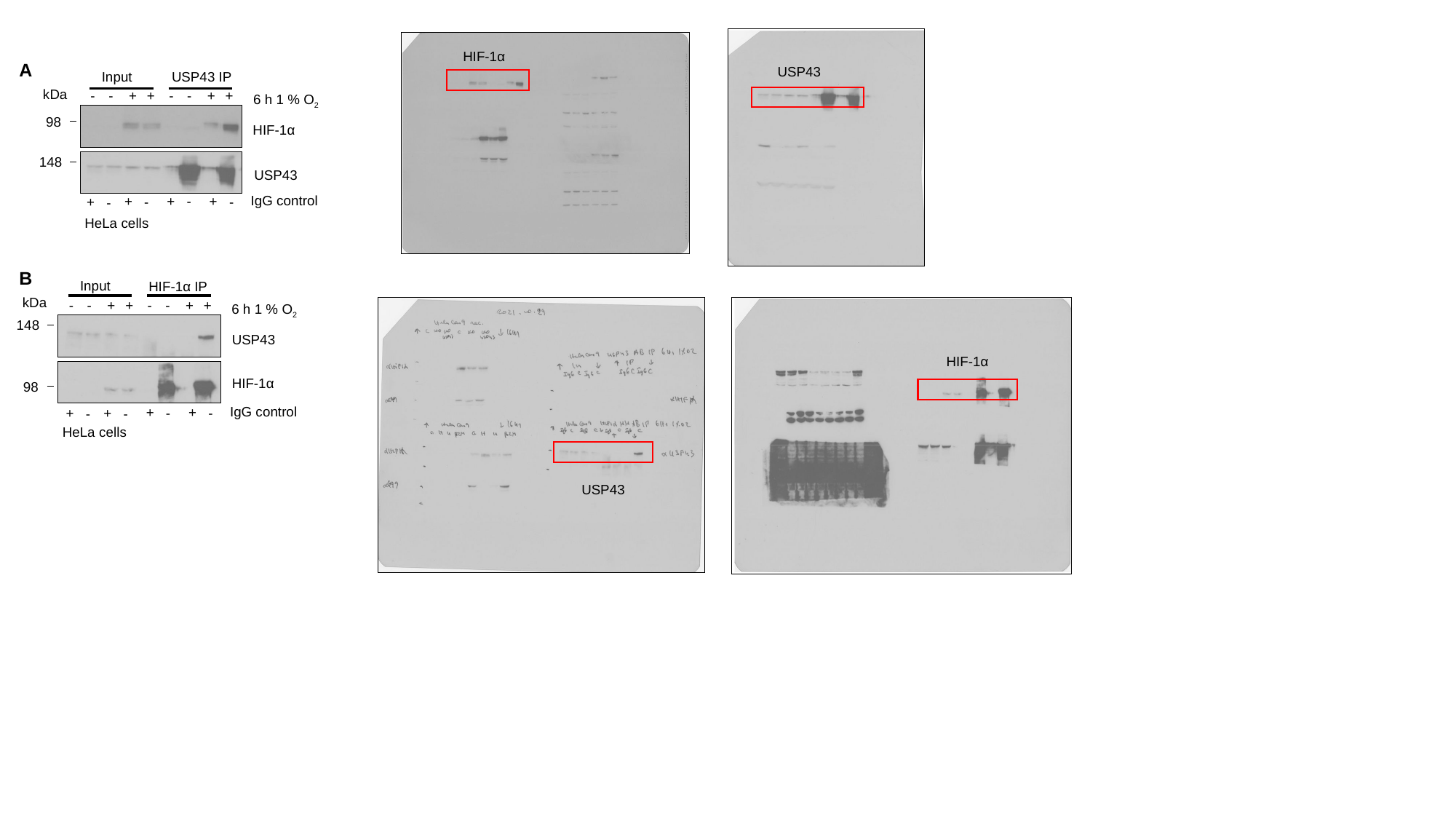

HIF-1α
A
USP43
Input
USP43 IP
kDa
-
-
+
+
-
-
+
+
6 h 1 % O2
98
HIF-1α
148
USP43
IgG control
+
+
+
-
+
-
-
-
HeLa cells
B
Input
HIF-1α IP
kDa
-
-
+
+
-
-
+
+
6 h 1 % O2
148
USP43
HIF-1α
HIF-1α
98
IgG control
+
+
+
-
+
-
-
-
HeLa cells
USP43
