# Supplementary material for: Deubiquitinating enzyme mutagenesis screens identify a USP43-dependent HIF-1 transcriptional response
Source: EMBO J. 2024 Jul 15;43(17):8. doi: 10.1038/s44318-024-00166-6 (PMC11377827; doi:10.1038/s44318-024-00166-6)
Supplement: Supplementary file 8 — Source data Fig. 4 [file 44318_2024_166_MOESM8_ESM.zip › Figure 4/F4 E UbiQ WB.pptx]

## Slide 1
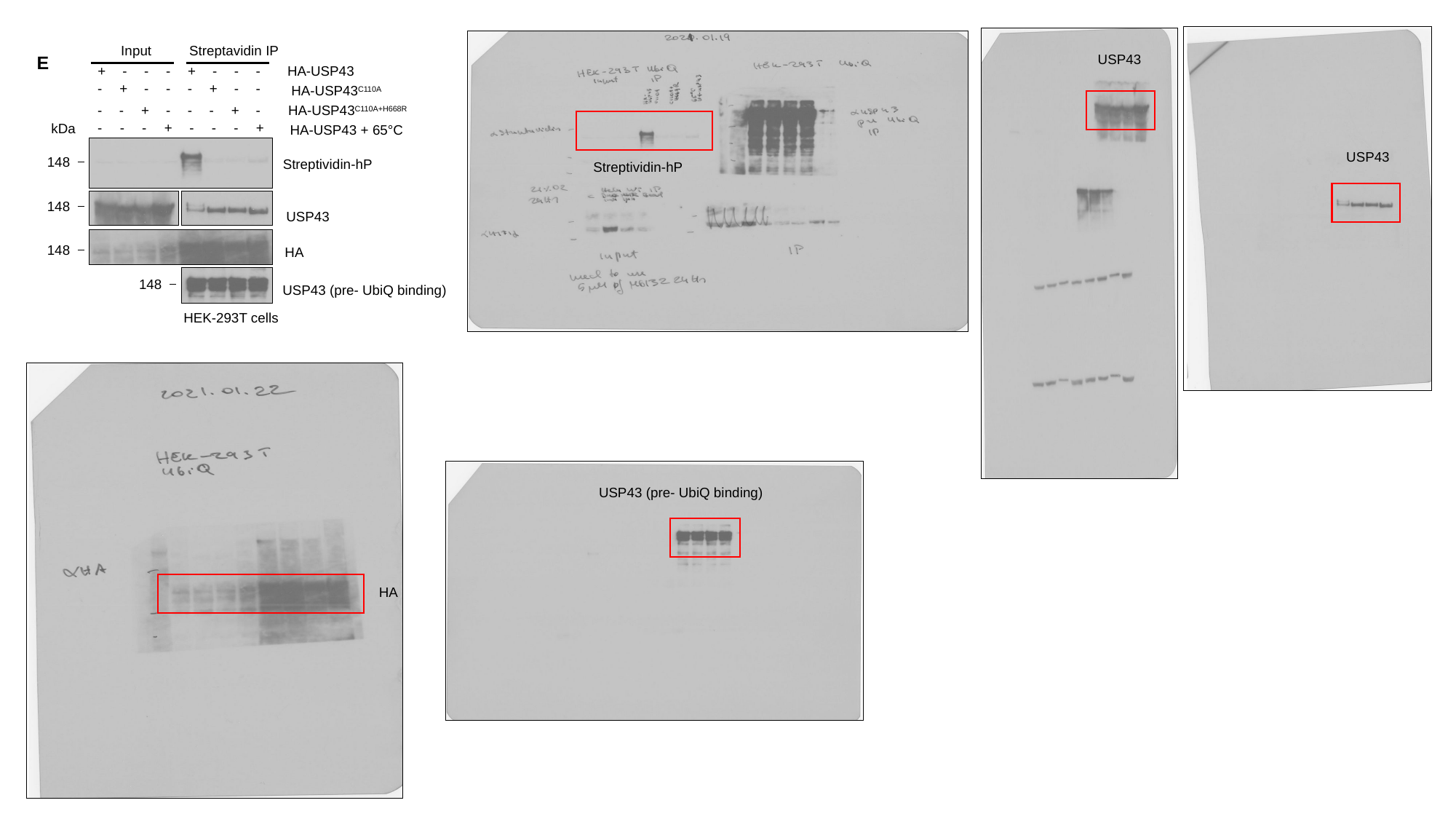

Streptavidin IP
Input
E
USP43
HA-USP43
+
-
-
-
+
-
-
-
-
+
-
-
-
+
-
-
HA-USP43C110A
-
-
+
-
-
-
+
-
HA-USP43C110A+H668R
-
-
-
+
-
-
-
+
kDa
HA-USP43 + 65°C
USP43
148
Streptividin-hP
Streptividin-hP
148
USP43
148
HA
148
USP43 (pre- UbiQ binding)
HEK-293T cells
USP43 (pre- UbiQ binding)
HA
