# Supplementary material for: Deubiquitinating enzyme mutagenesis screens identify a USP43-dependent HIF-1 transcriptional response
Source: EMBO J. 2024 Jul 15;43(17):8. doi: 10.1038/s44318-024-00166-6 (PMC11377827; doi:10.1038/s44318-024-00166-6)
Supplement: Supplementary file 9 — Source data Fig. 5 [file 44318_2024_166_MOESM9_ESM.zip › Figure 5/F5 D and F.pptx]

## Slide 1
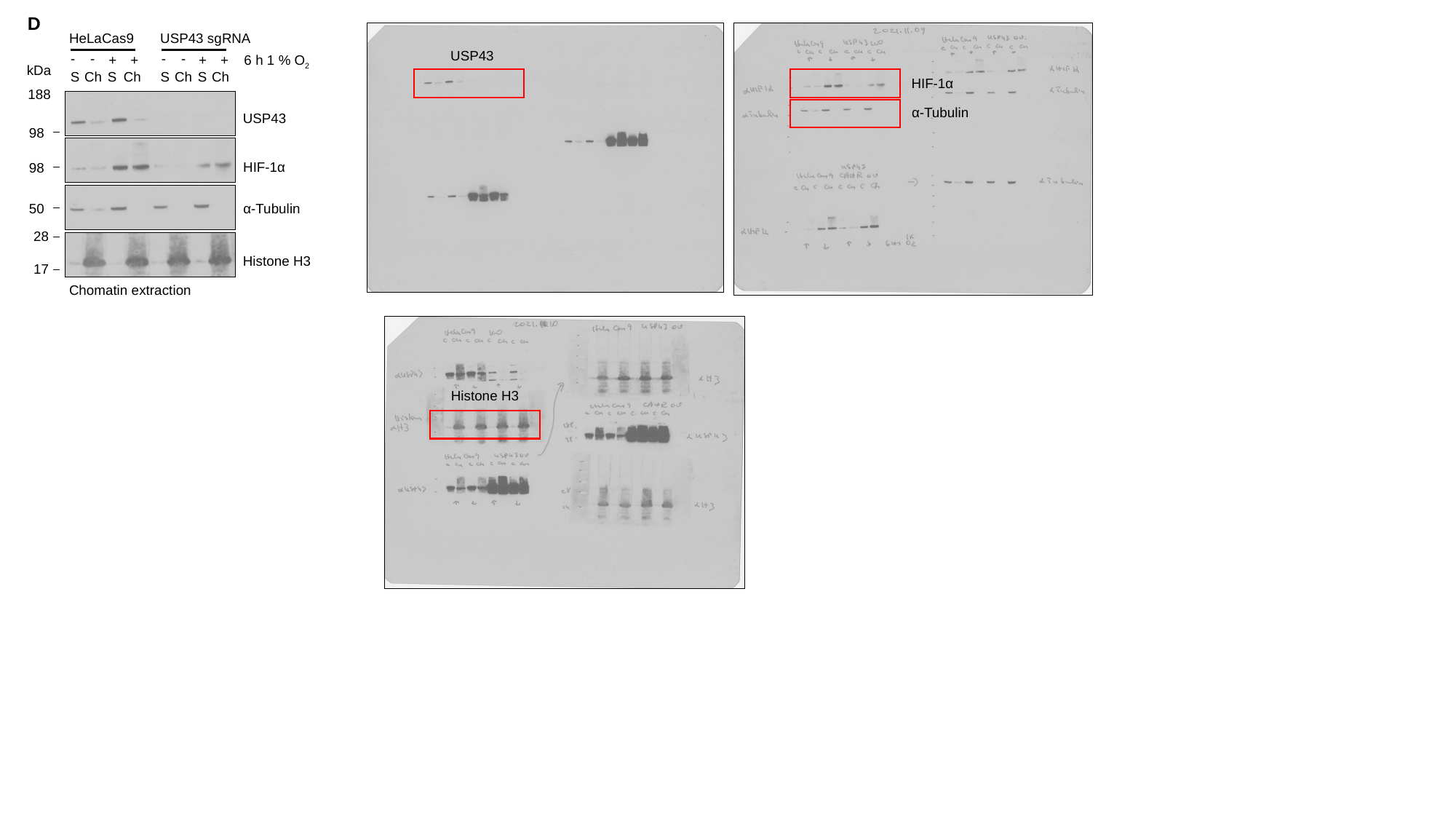

D
HeLaCas9
USP43 sgRNA
USP43
-
-
-
-
+
+
+
+
6 h 1 % O2
kDa
 S
 Ch
 S
Ch
 S
Ch
S
Ch
HIF-1α
188
α-Tubulin
USP43
98
HIF-1α
98
α-Tubulin
50
28
Histone H3
17
Chomatin extraction
Histone H3

## Slide 2
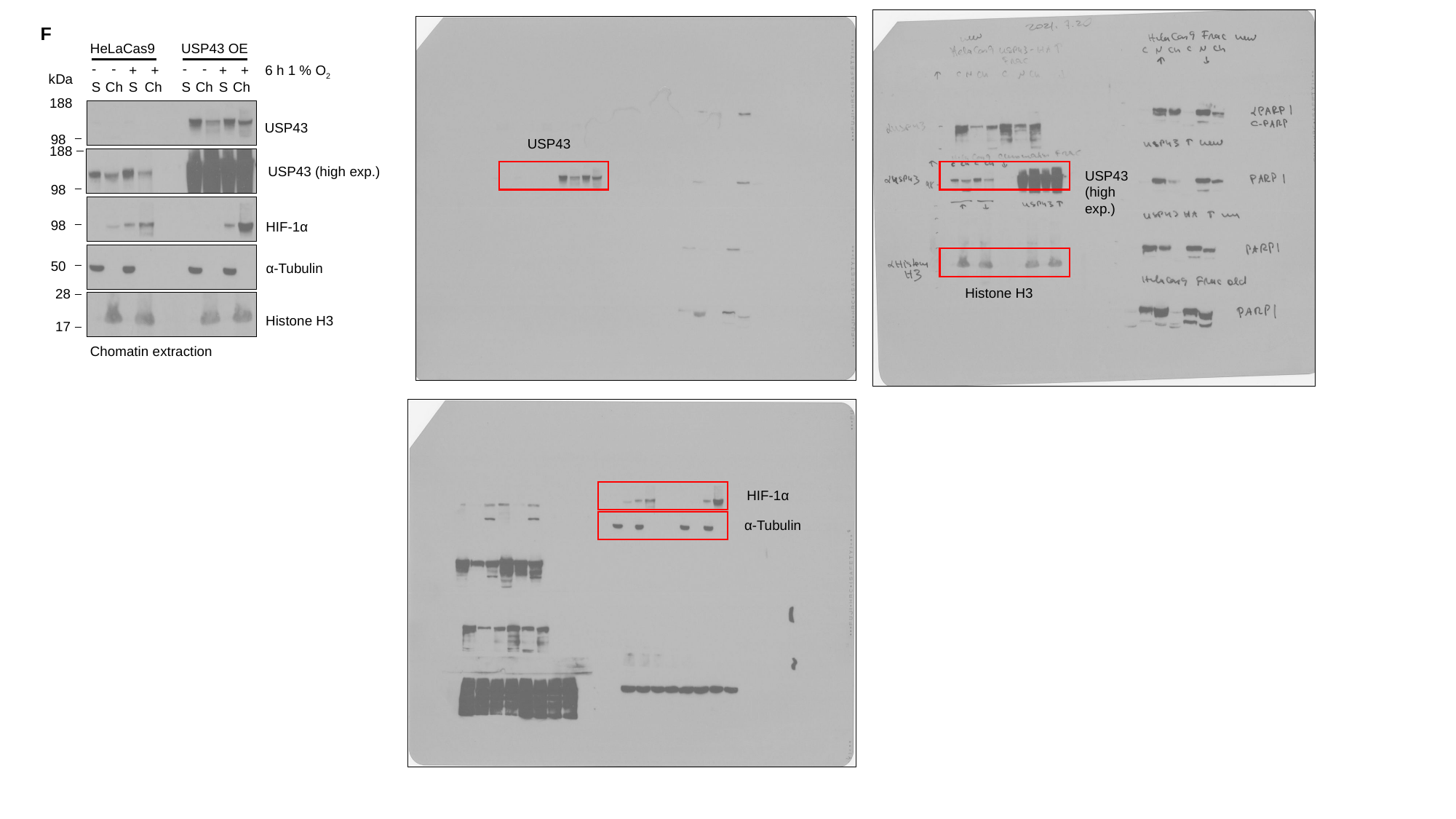

F
HeLaCas9
USP43 OE
-
-
-
-
+
+
+
+
6 h 1 % O2
kDa
 S
 Ch
 S
Ch
 S
Ch
S
Ch
188
USP43
98
USP43
188
USP43 (high exp.)
USP43 (high exp.)
98
98
HIF-1α
50
α-Tubulin
Histone H3
28
Histone H3
17
Chomatin extraction
HIF-1α
α-Tubulin
