# Supplementary material for: Deubiquitinating enzyme mutagenesis screens identify a USP43-dependent HIF-1 transcriptional response
Source: EMBO J. 2024 Jul 15;43(17):8. doi: 10.1038/s44318-024-00166-6 (PMC11377827; doi:10.1038/s44318-024-00166-6)
Supplement: Supplementary file 11 — Source data Fig. 7 [file 44318_2024_166_MOESM11_ESM.zip › Figure 7/F7 A, B, D, F, K WB.pptx]

## Slide 1
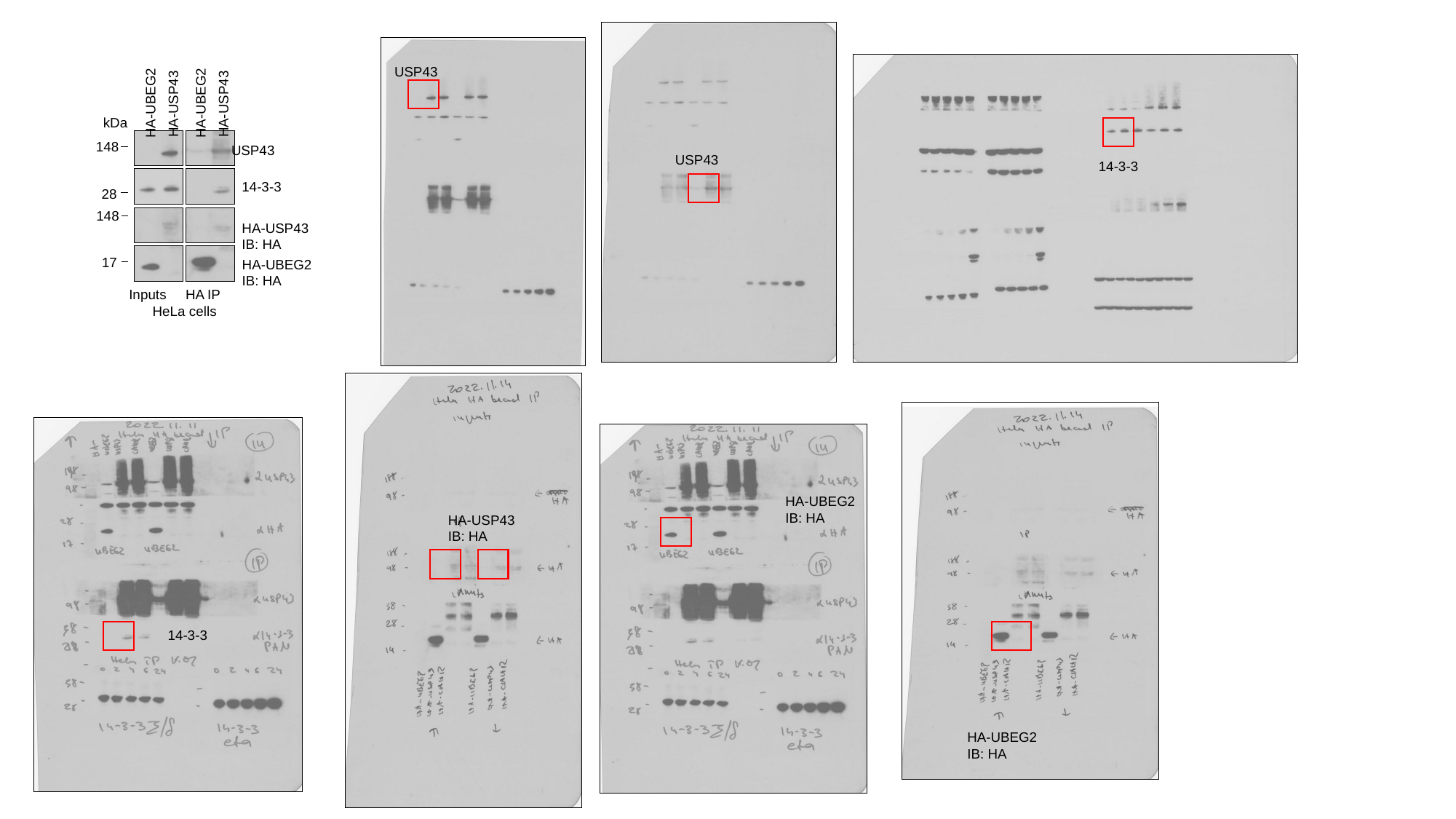

USP43
HA-UBEG2
HA-UBEG2
HA-USP43
HA-USP43
kDa
148
USP43
USP43
14-3-3
14-3-3
28
148
HA-USP43
IB: HA
17
HA-UBEG2
IB: HA
Inputs
HA IP
HeLa cells
HA-UBEG2
IB: HA
HA-USP43
IB: HA
14-3-3
HA-UBEG2
IB: HA

## Slide 2
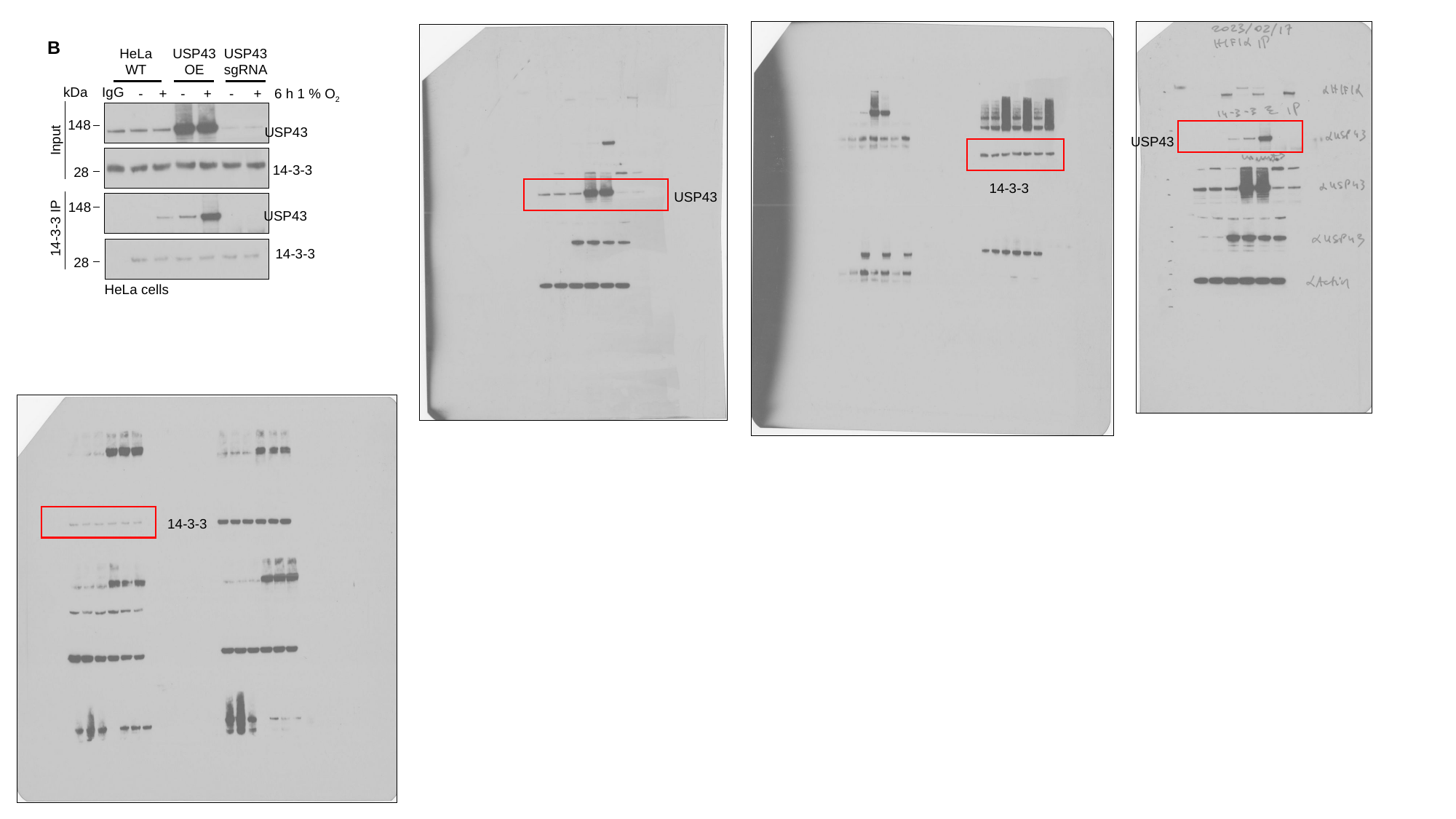

B
HeLa WT
USP43 OE
USP43 sgRNA
IgG
kDa
-
+
-
+
-
+
6 h 1 % O2
148
USP43
USP43
Input
14-3-3
28
14-3-3
USP43
148
USP43
14-3-3 IP
14-3-3
28
HeLa cells
14-3-3

## Slide 3
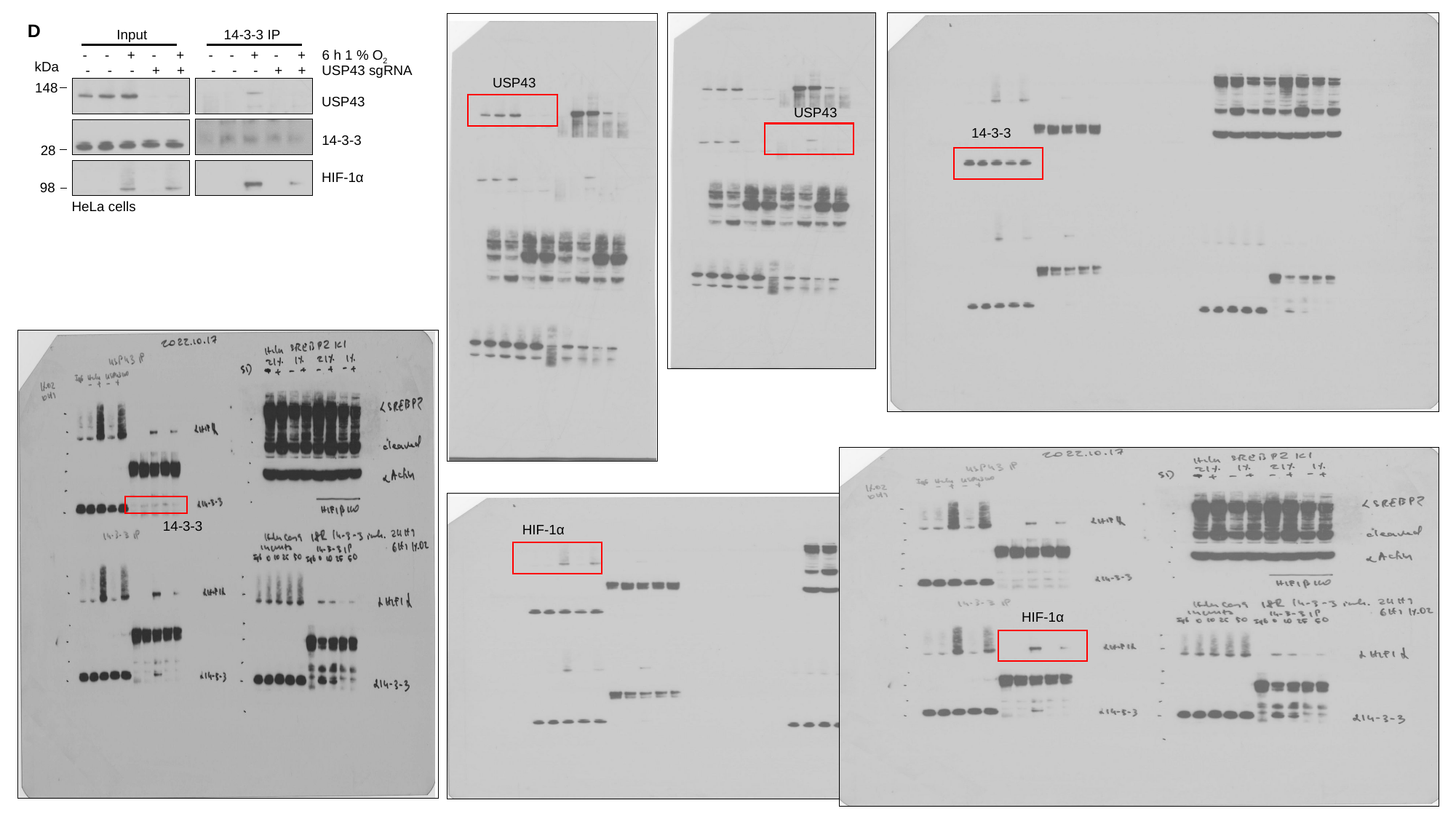

D
Input
14-3-3 IP
6 h 1 % O2
-
-
+
-
+
-
-
+
-
+
kDa
-
-
-
+
+
-
-
-
+
+
USP43 sgRNA
USP43
148
USP43
USP43
14-3-3
14-3-3
28
HIF-1α
98
HeLa cells
14-3-3
HIF-1α
HIF-1α

## Slide 4
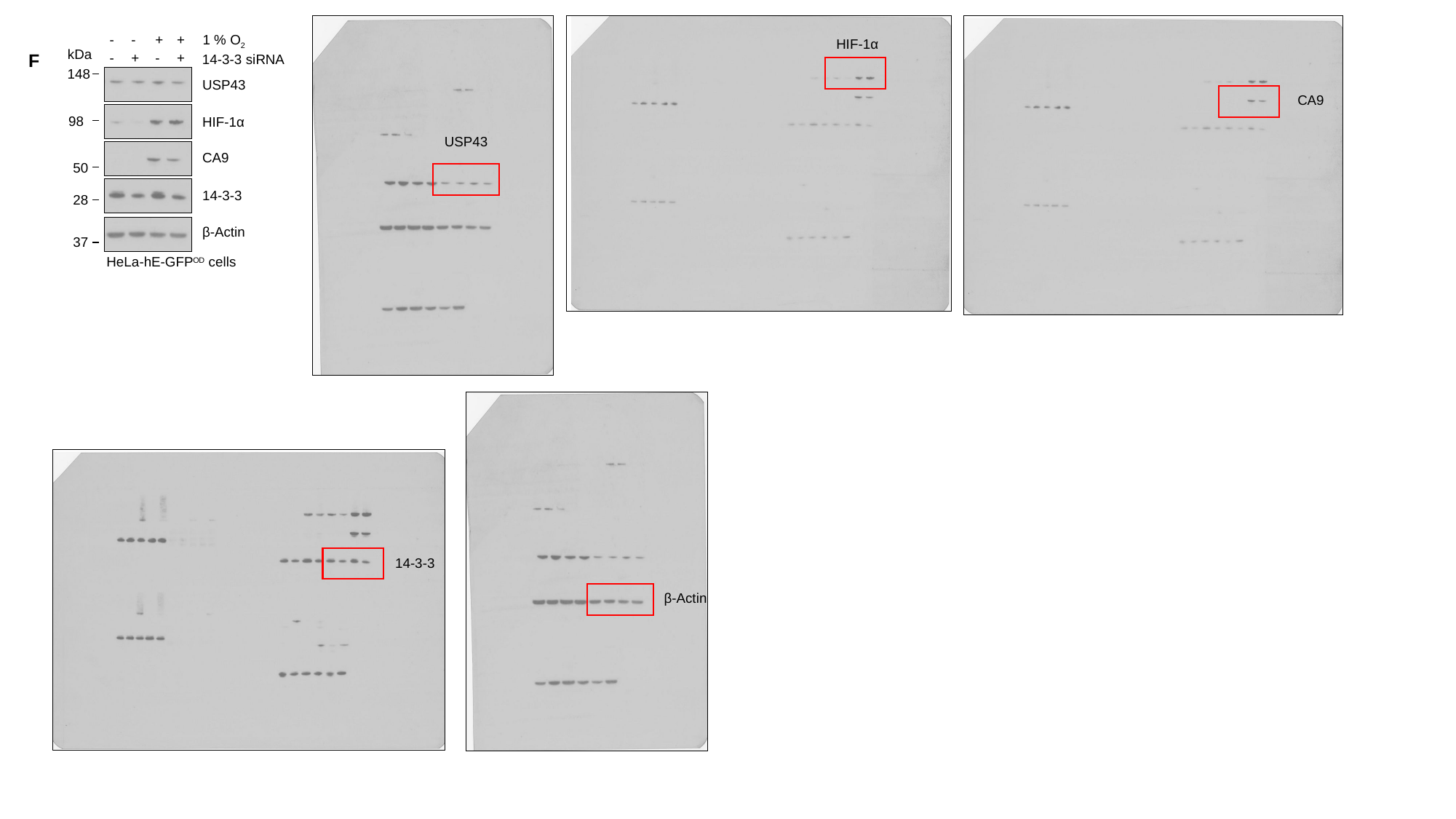

-
-
+
+
1 % O2
HIF-1α
kDa
F
-
+
-
+
14-3-3 siRNA
148
USP43
CA9
98
HIF-1α
USP43
CA9
50
14-3-3
28
β-Actin
37
HeLa-hE-GFPOD cells
14-3-3
β-Actin

## Slide 5
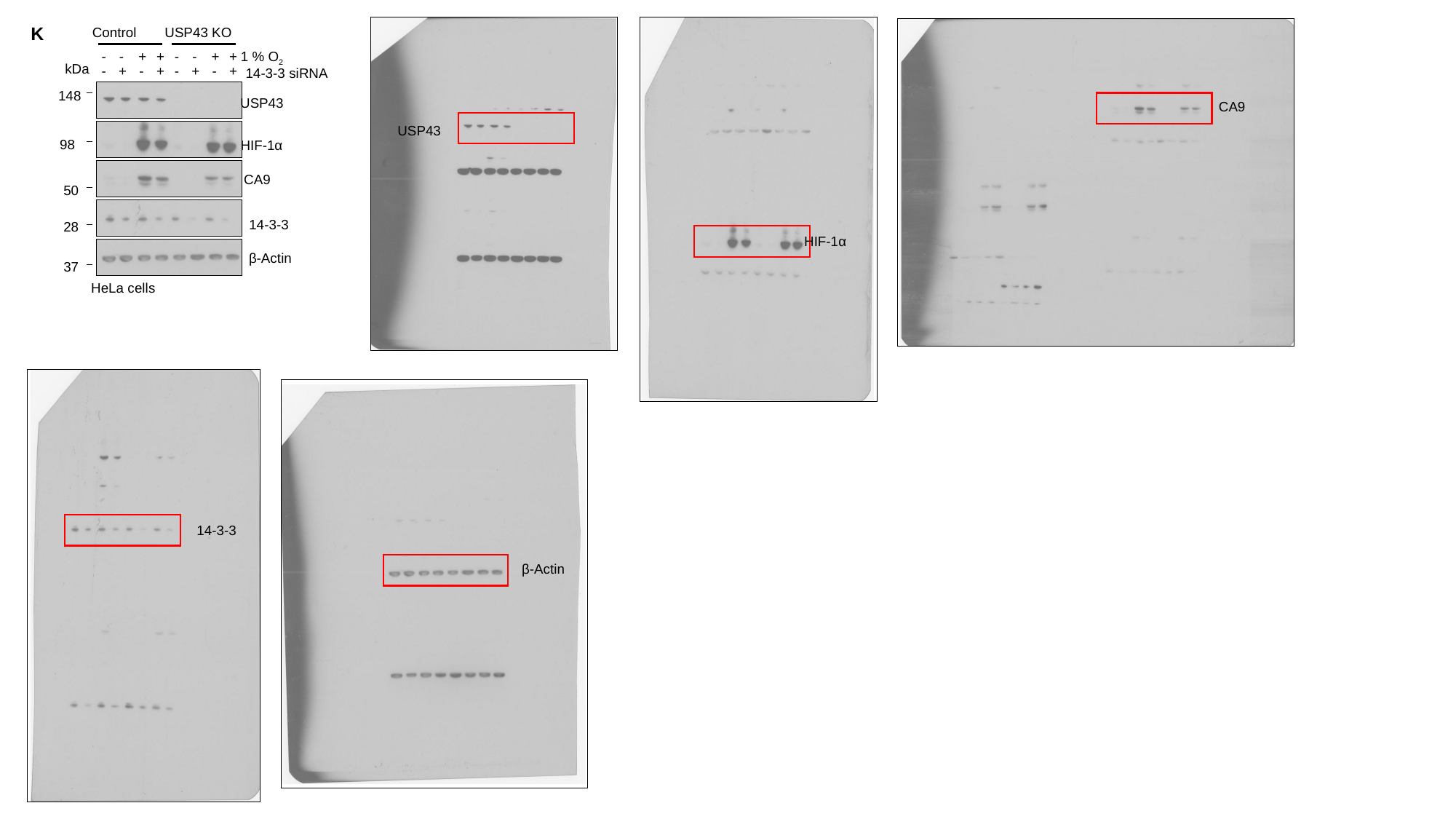

K
USP43 KO
Control
-
-
+
+
-
-
+
+
1 % O2
kDa
-
+
-
+
-
+
-
+
14-3-3 siRNA
148
USP43
CA9
USP43
98
HIF-1α
CA9
50
14-3-3
28
HIF-1α
β-Actin
37
HeLa cells
14-3-3
β-Actin
