# Supplementary material for: Deubiquitinating enzyme mutagenesis screens identify a USP43-dependent HIF-1 transcriptional response
Source: EMBO J. 2024 Jul 15;43(17):8. doi: 10.1038/s44318-024-00166-6 (PMC11377827; doi:10.1038/s44318-024-00166-6)
Supplement: Supplementary file 12 — Extended View and Appendix Source Data [file 44318_2024_166_MOESM12_ESM.zip › Extended View and Appendix Source Data/Appendix Figure S1/S1 A, B, C, E WB.pptx]

## Slide 1
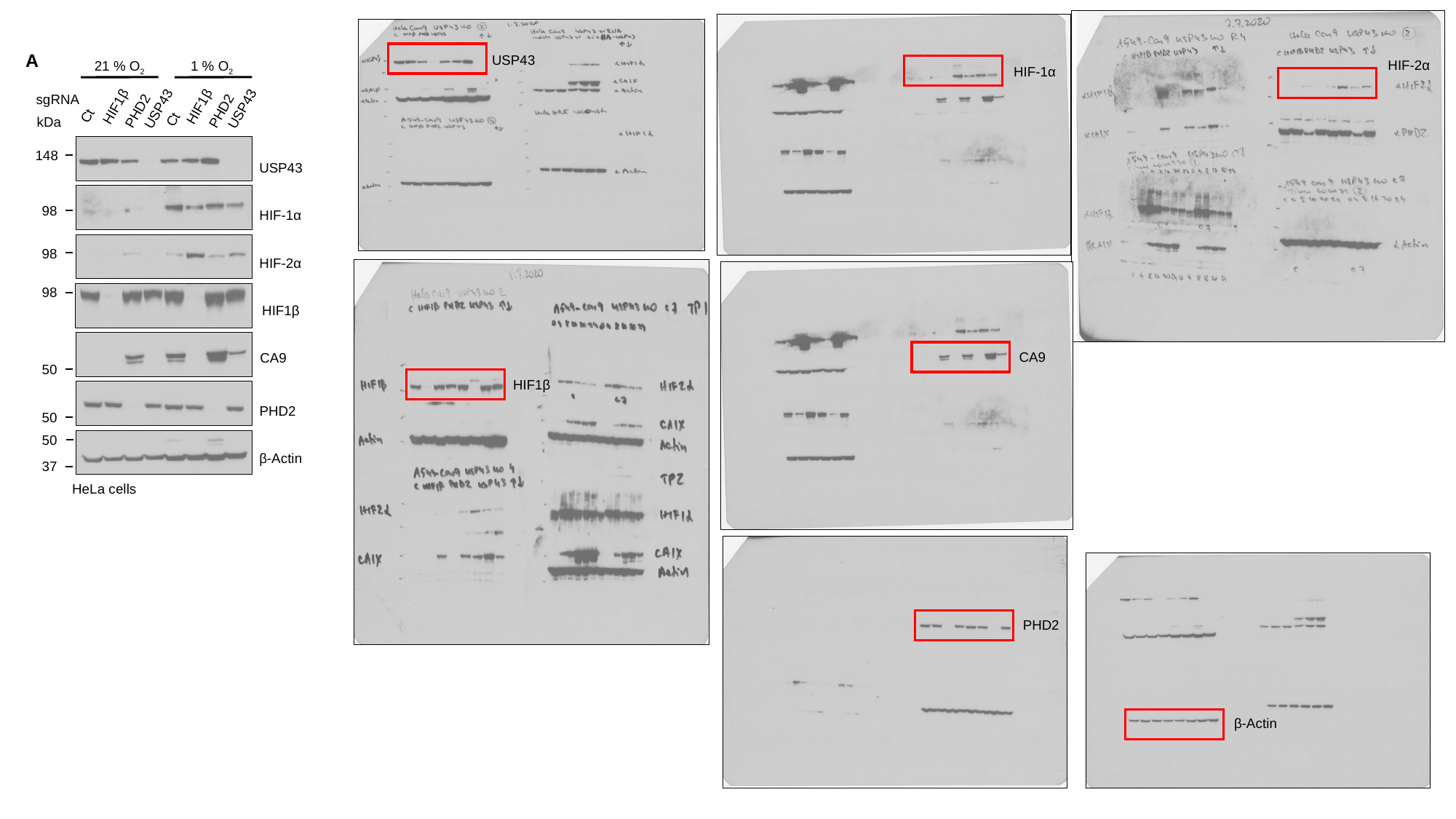

A
USP43
HIF-2α
21 % O2
1 % O2
HIF-1α
sgRNA
HIF1β
HIF1β
USP43
USP43
PHD2
PHD2
Ct
Ct
kDa
148
USP43
98
HIF-1α
98
HIF-2α
98
HIF1β
CA9
CA9
50
HIF1β
PHD2
50
50
β-Actin
37
HeLa cells
PHD2
β-Actin

## Slide 2
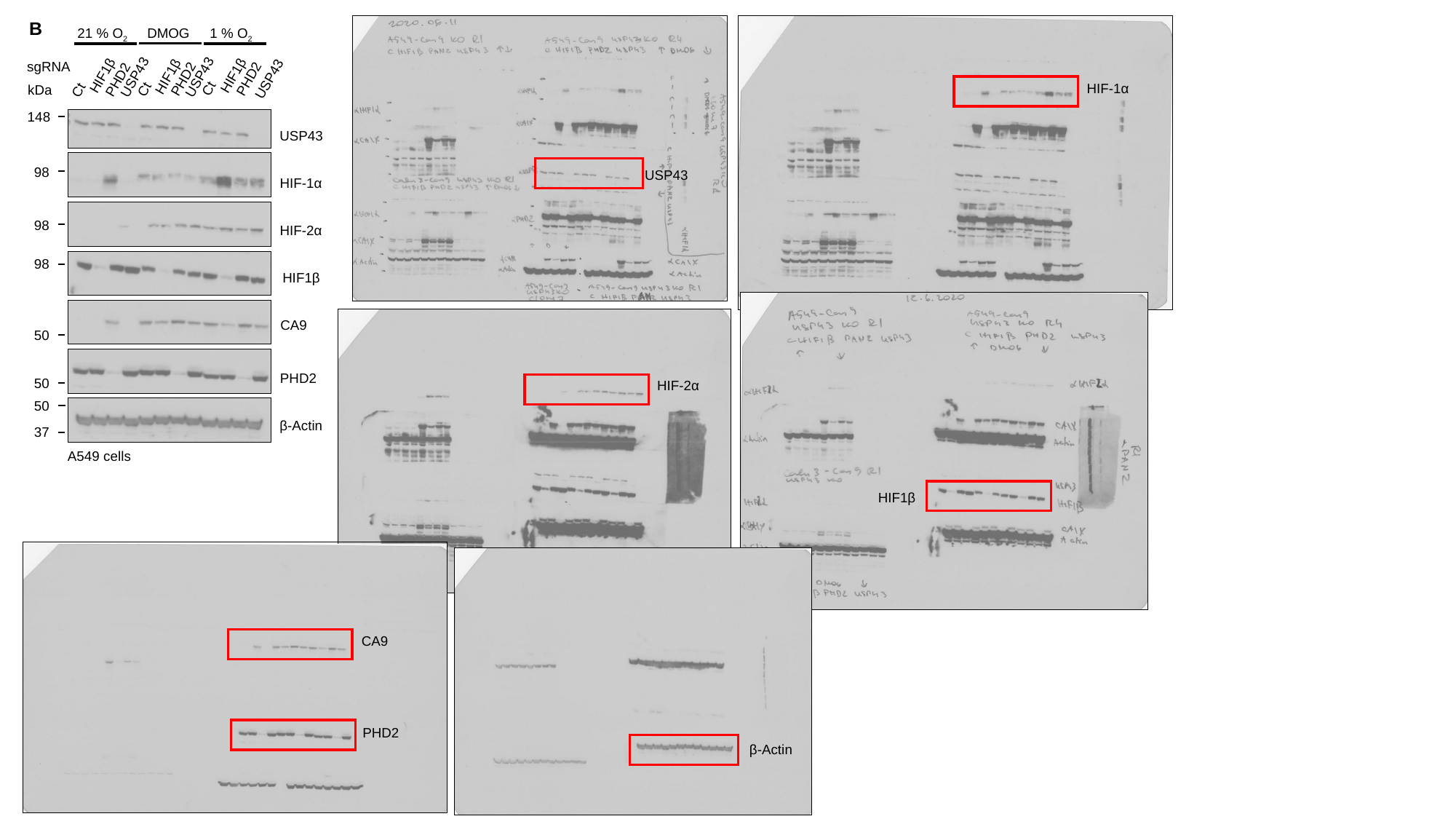

B
21 % O2
DMOG
1 % O2
sgRNA
HIF1β
HIF1β
HIF1β
USP43
USP43
PHD2
USP43
PHD2
PHD2
HIF-1α
Ct
Ct
kDa
Ct
148
USP43
98
USP43
HIF-1α
98
HIF-2α
98
HIF1β
CA9
50
PHD2
50
HIF-2α
50
β-Actin
37
A549 cells
HIF1β
CA9
PHD2
β-Actin

## Slide 3
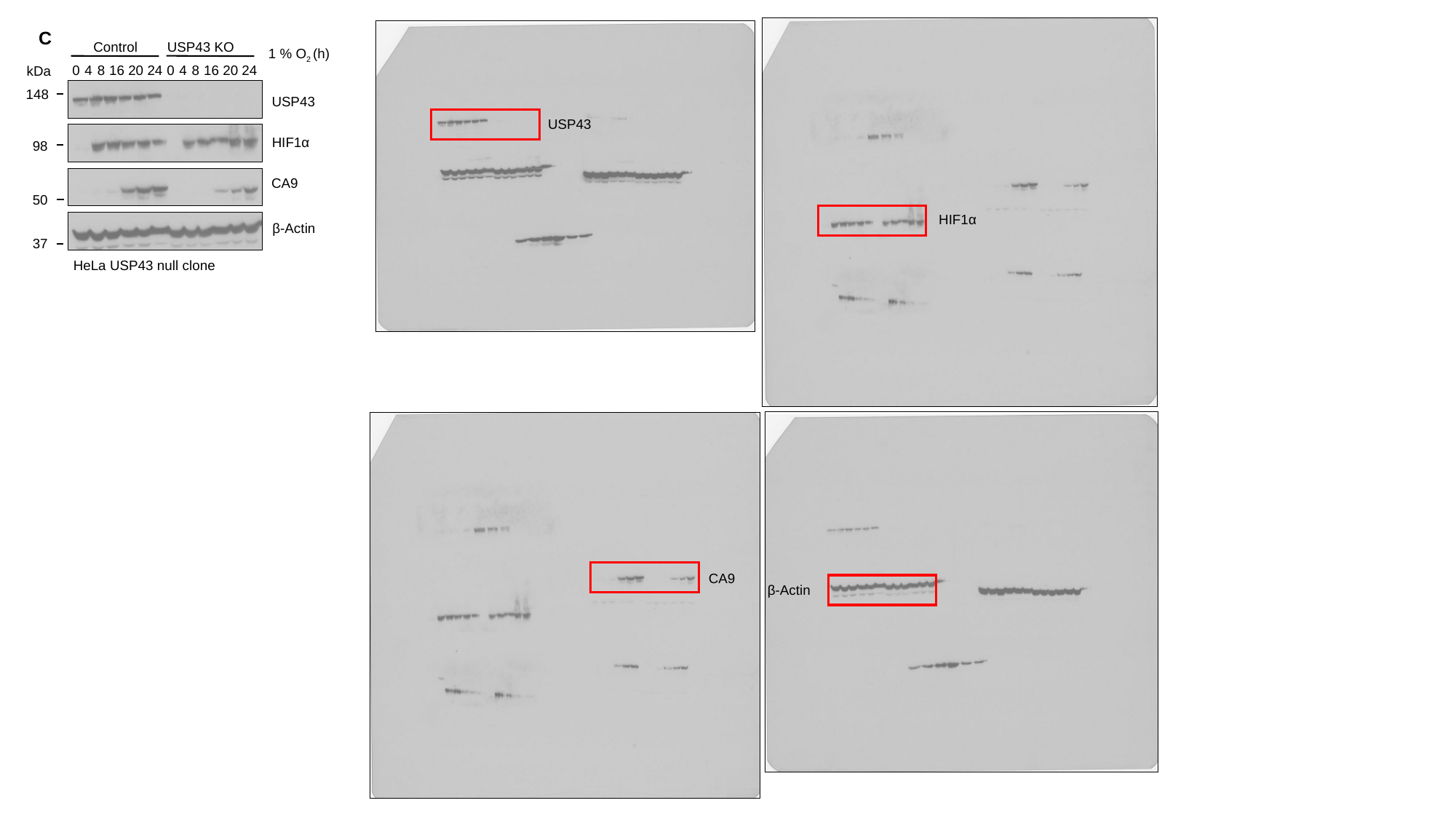

C
USP43 KO
Control
1 % O2 (h)
0
4
8
16
20
24
0
4
8
16
20
24
kDa
148
USP43
USP43
HIF1α
98
CA9
50
HIF1α
β-Actin
37
HeLa USP43 null clone
CA9
β-Actin

## Slide 4
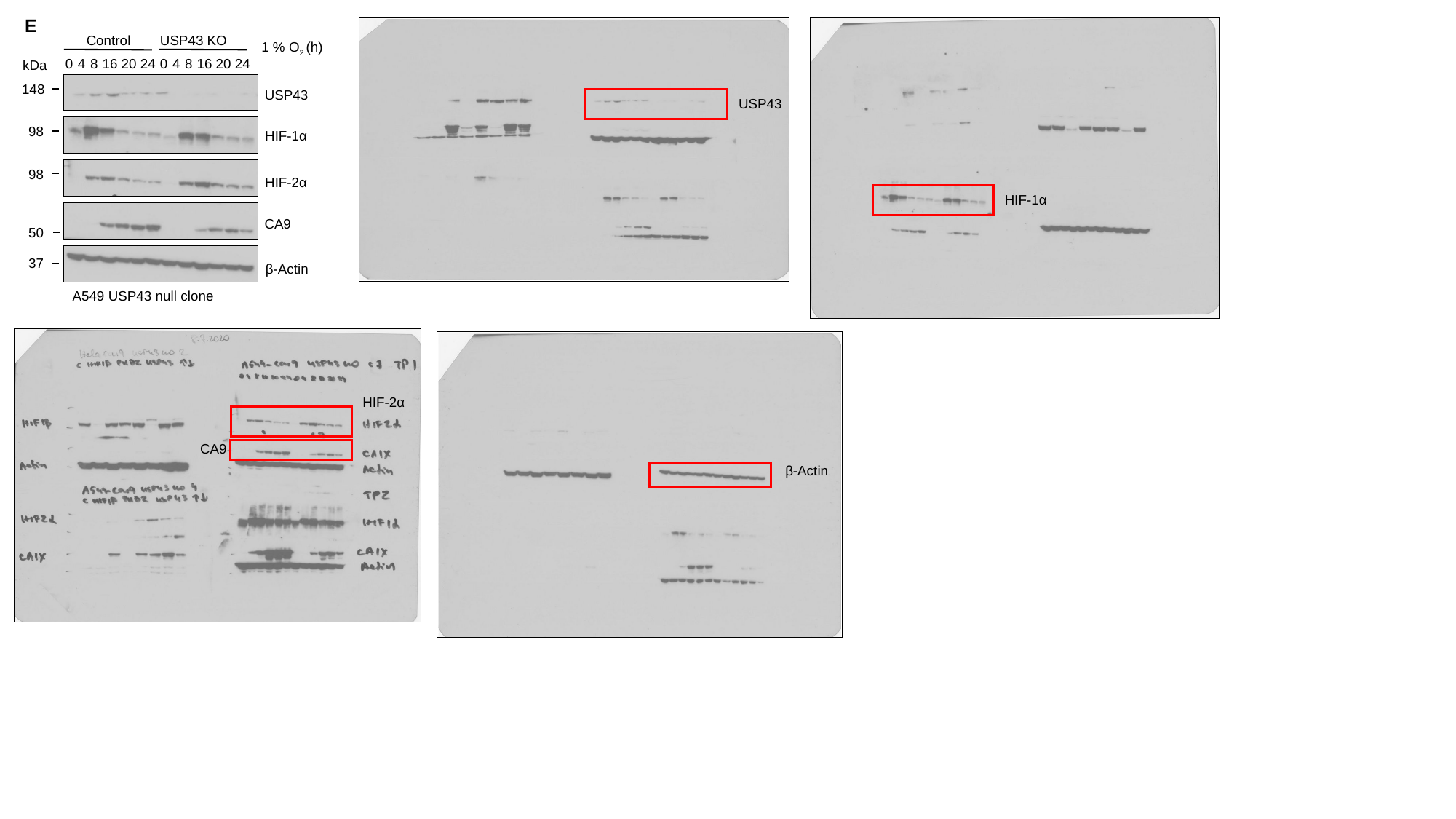

E
USP43 KO
Control
1 % O2 (h)
0
4
8
16
20
24
0
4
8
16
20
24
kDa
148
USP43
USP43
98
HIF-1α
98
HIF-2α
HIF-1α
CA9
50
37
β-Actin
A549 USP43 null clone
HIF-2α
CA9
β-Actin
