# Supplementary material for: Deubiquitinating enzyme mutagenesis screens identify a USP43-dependent HIF-1 transcriptional response
Source: EMBO J. 2024 Jul 15;43(17):8. doi: 10.1038/s44318-024-00166-6 (PMC11377827; doi:10.1038/s44318-024-00166-6)
Supplement: Supplementary file 12 — Extended View and Appendix Source Data [file 44318_2024_166_MOESM12_ESM.zip › Extended View and Appendix Source Data/Figure EV4/EV4 A-D.pptx]

## Slide 1
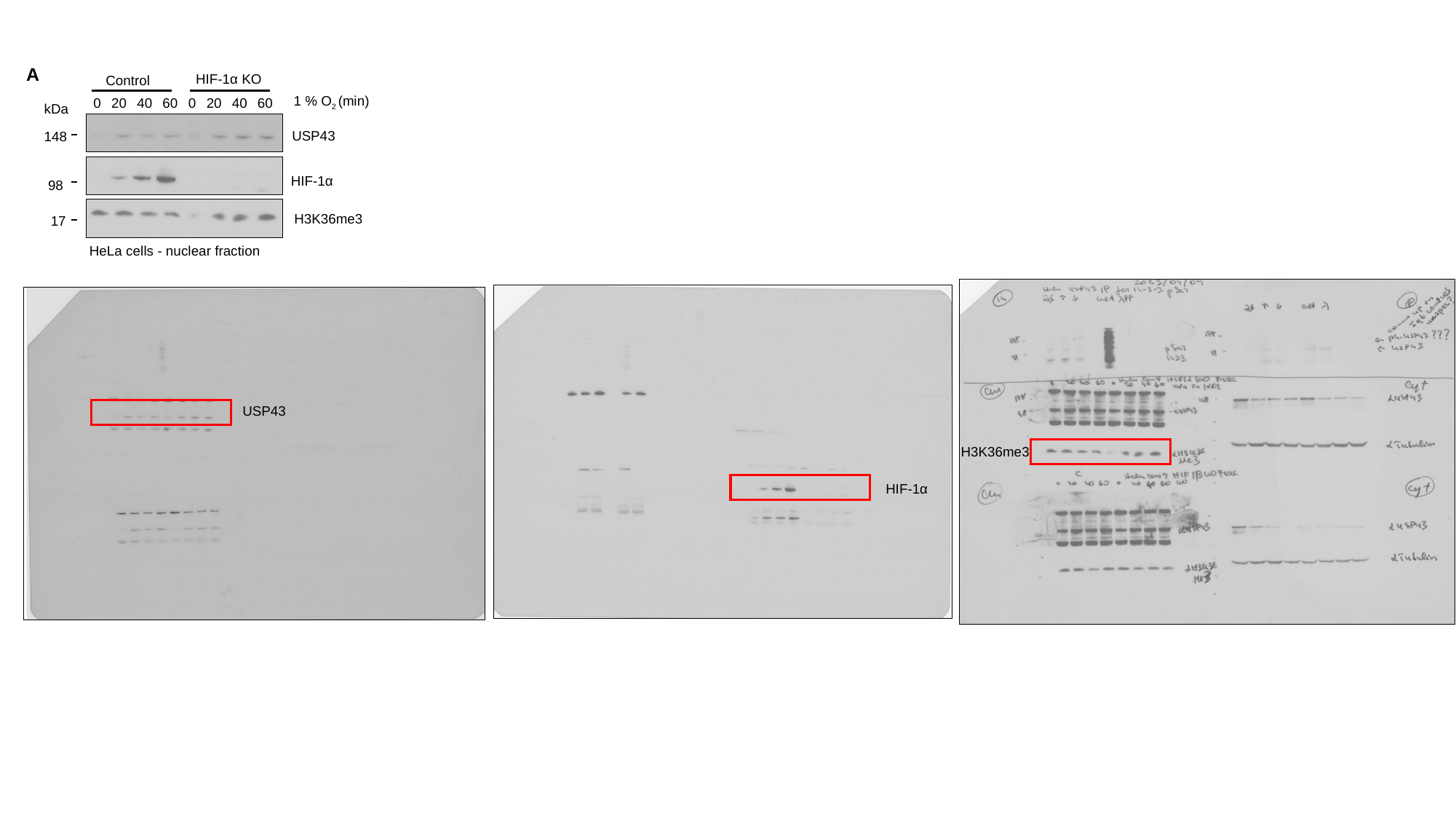

A
HIF-1α KO
Control
1 % O2 (min)
0
20
40
60
0
20
40
60
kDa
USP43
148
HIF-1α
98
H3K36me3
17
HeLa cells - nuclear fraction
USP43
H3K36me3
HIF-1α

## Slide 2
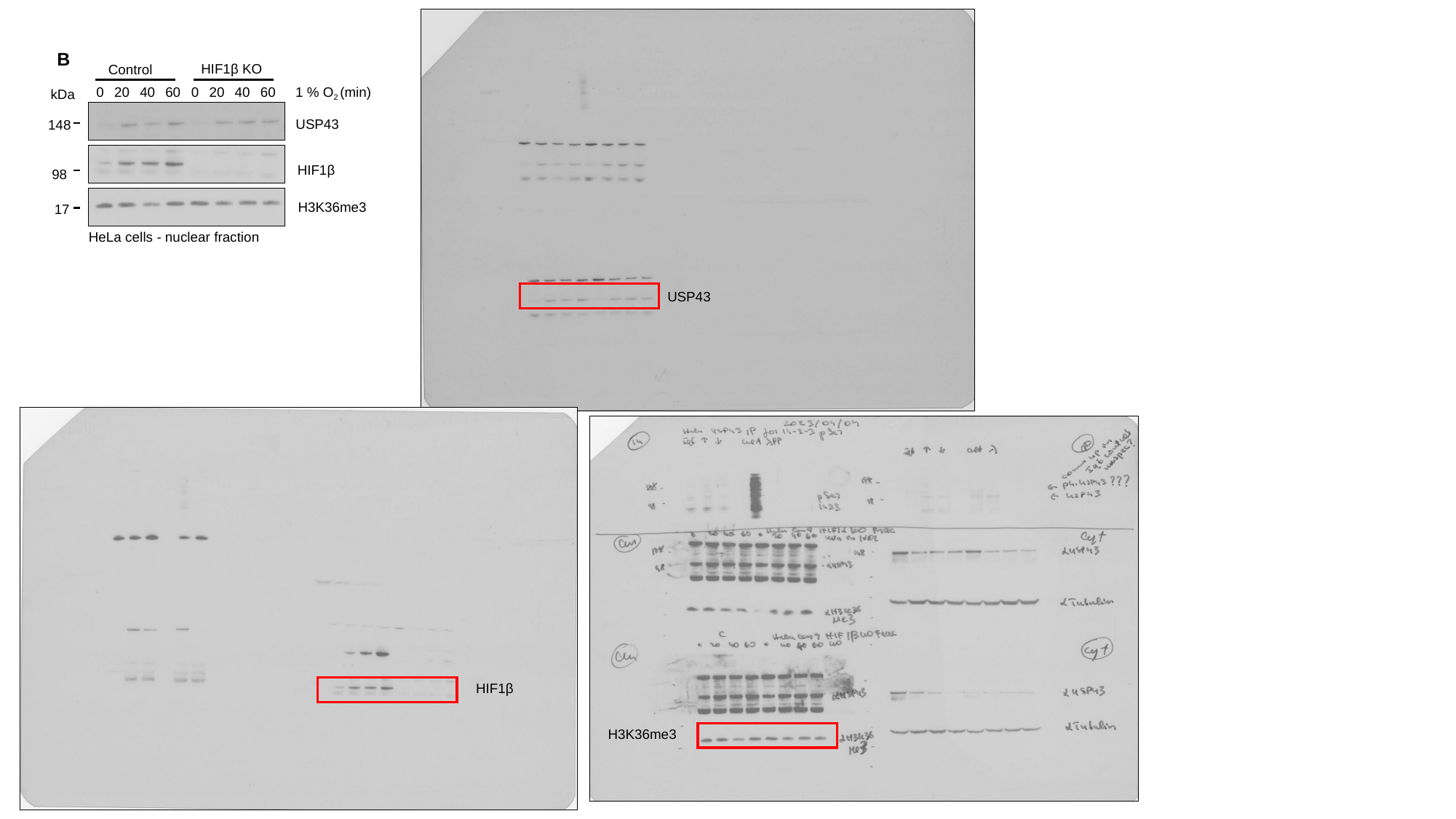

B
HIF1β KO
Control
1 % O2 (min)
0
20
40
60
0
20
40
60
kDa
USP43
148
HIF1β
98
H3K36me3
17
HeLa cells - nuclear fraction
USP43
HIF1β
H3K36me3

## Slide 3
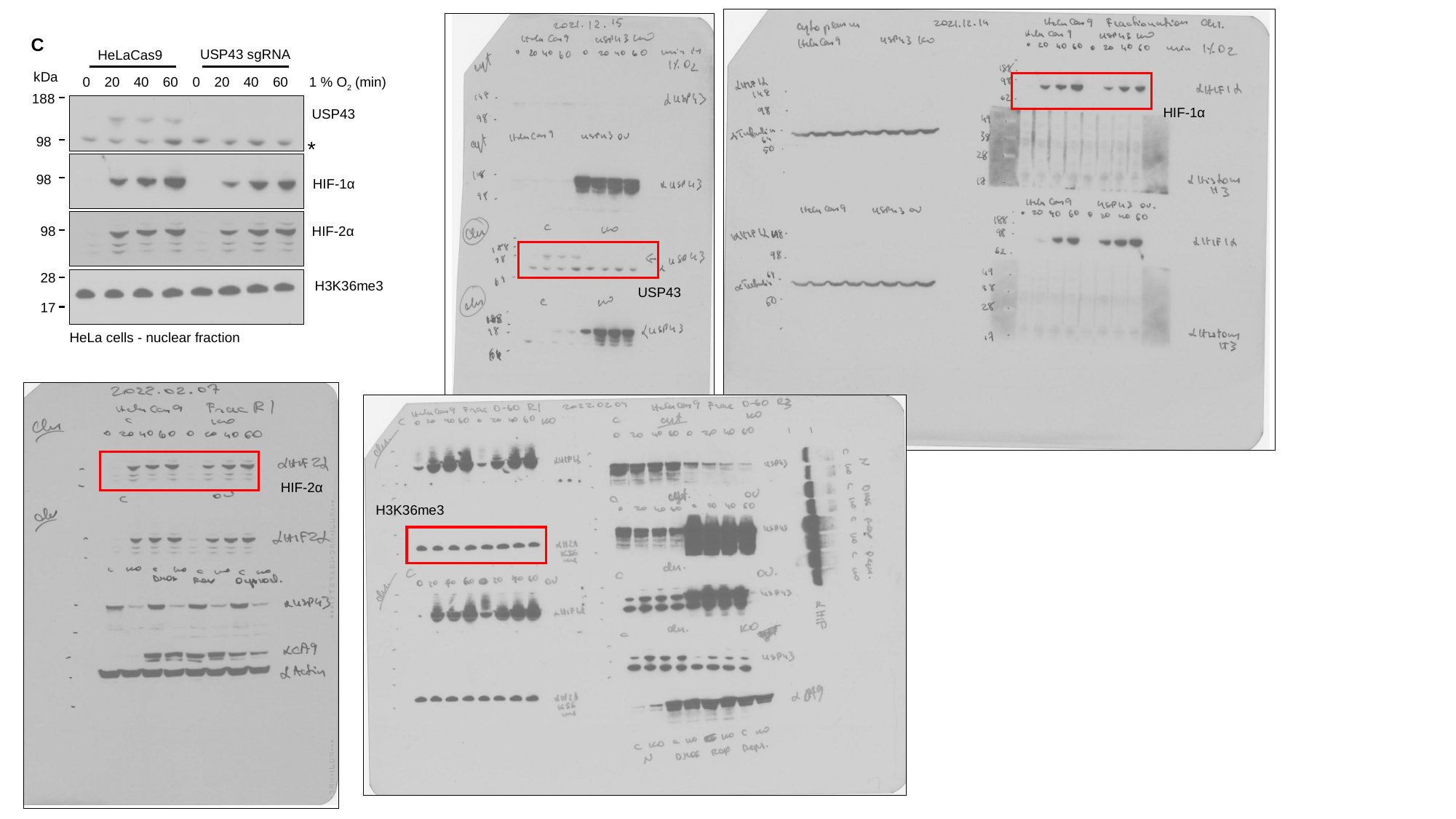

C
USP43 sgRNA
HeLaCas9
kDa
1 % O2 (min)
0
20
40
60
0
20
40
60
188
HIF-1α
USP43
98
*
98
HIF-1α
HIF-2α
98
28
H3K36me3
USP43
17
HeLa cells - nuclear fraction
HIF-2α
H3K36me3

## Slide 4
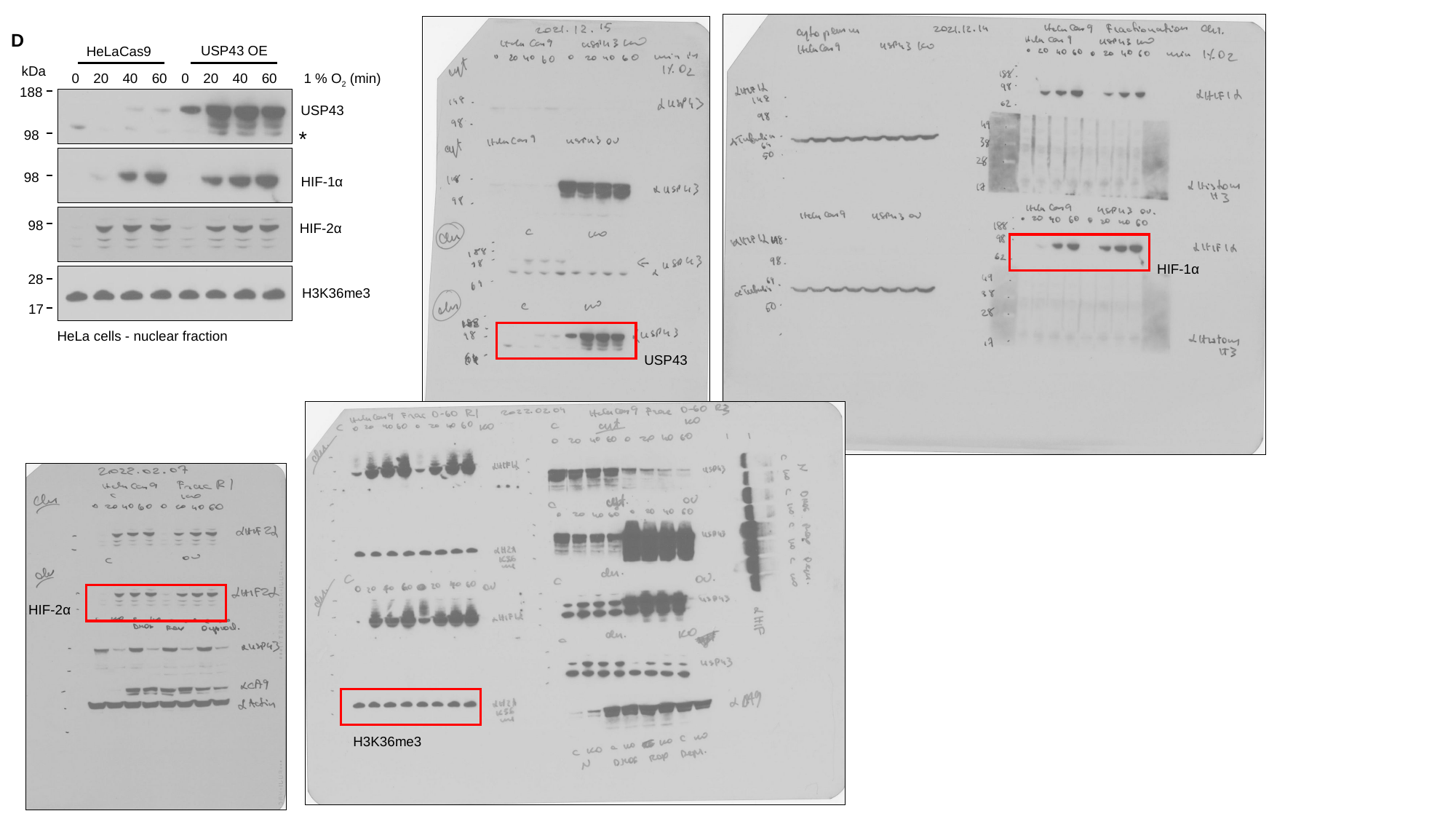

D
USP43 OE
HeLaCas9
kDa
1 % O2 (min)
0
20
40
60
0
20
40
60
188
USP43
*
98
98
HIF-1α
98
HIF-2α
HIF-1α
28
H3K36me3
17
HeLa cells - nuclear fraction
USP43
HIF-2α
H3K36me3

## Slide 5
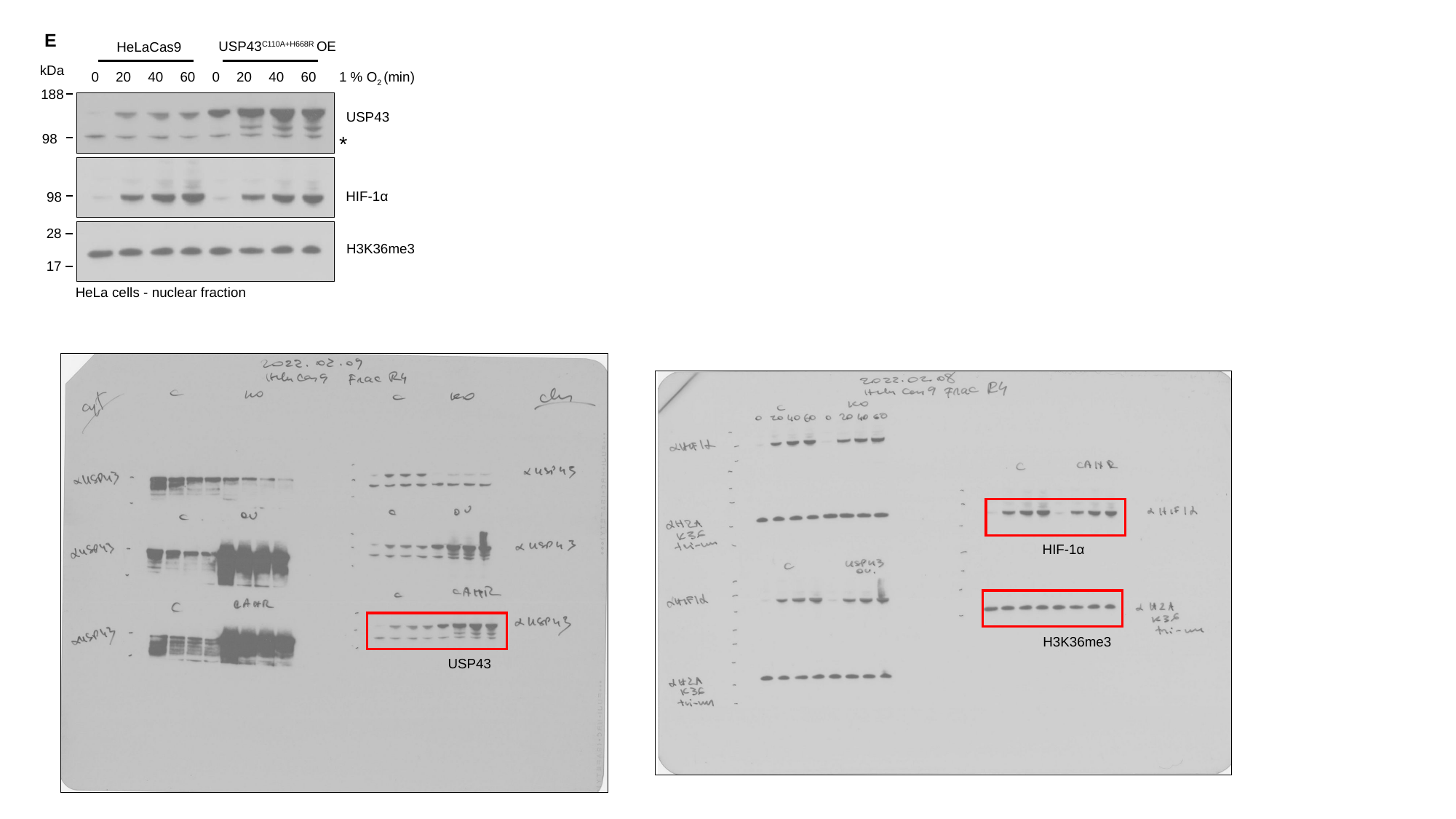

E
USP43C110A+H668R OE
HeLaCas9
kDa
1 % O2 (min)
0
20
40
60
0
20
40
60
188
USP43
98
*
HIF-1α
98
28
H3K36me3
17
HeLa cells - nuclear fraction
HIF-1α
H3K36me3
USP43
