# Supplementary material for: Deubiquitinating enzyme mutagenesis screens identify a USP43-dependent HIF-1 transcriptional response
Source: EMBO J. 2024 Jul 15;43(17):8. doi: 10.1038/s44318-024-00166-6 (PMC11377827; doi:10.1038/s44318-024-00166-6)
Supplement: Supplementary file 12 — Extended View and Appendix Source Data [file 44318_2024_166_MOESM12_ESM.zip › Extended View and Appendix Source Data/Figure EV3/EV3 A-F WB.pptx]

## Slide 1
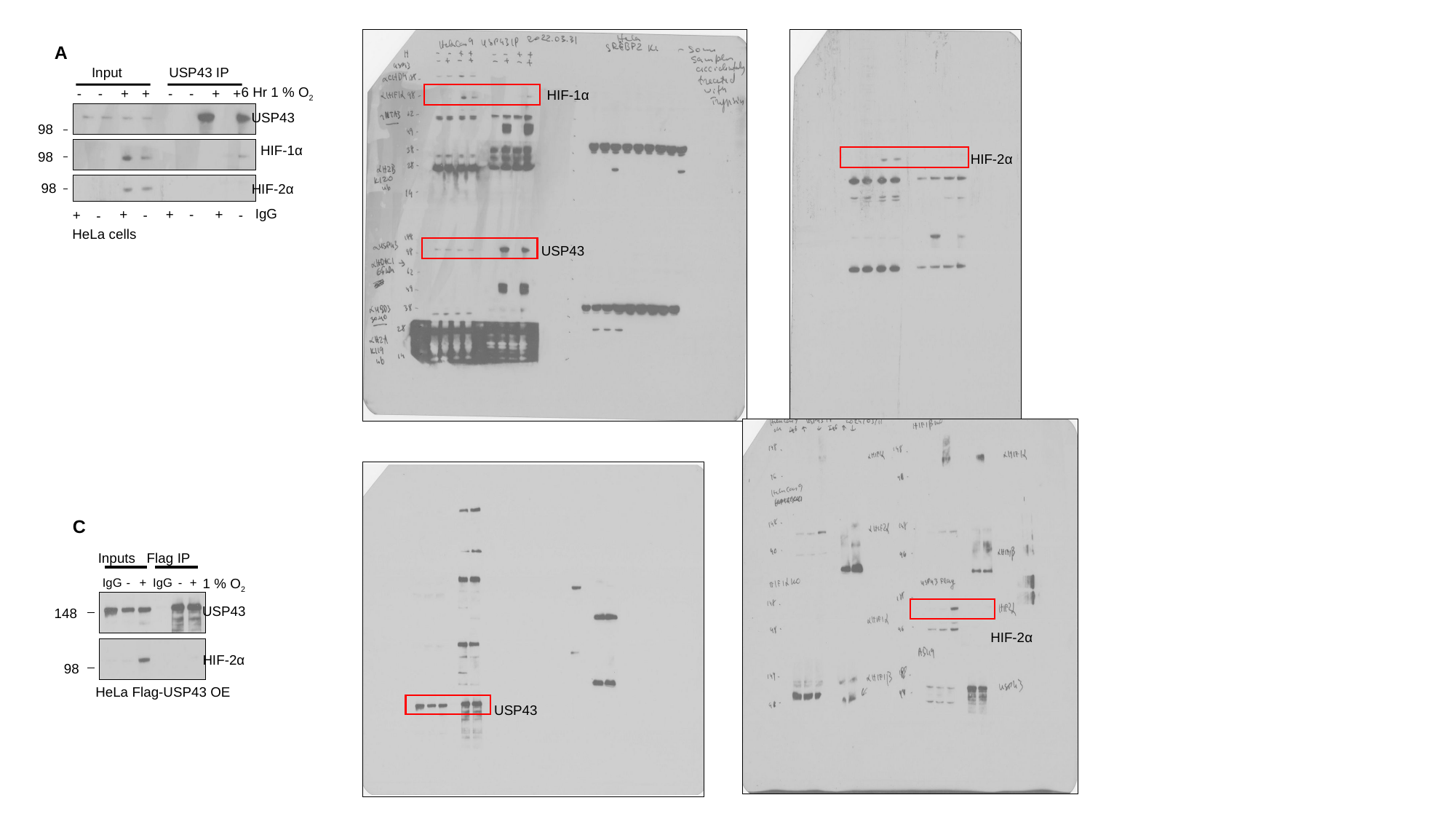

A
Input
USP43 IP
6 Hr 1 % O2
-
-
+
+
-
-
+
+
HIF-1α
USP43
98
HIF-1α
98
HIF-2α
98
HIF-2α
IgG
+
+
+
-
+
-
-
-
HeLa cells
USP43
C
Inputs
Flag IP
1 % O2
IgG
-
+
IgG
-
+
USP43
148
HIF-2α
HIF-2α
98
HeLa Flag-USP43 OE
USP43

## Slide 2
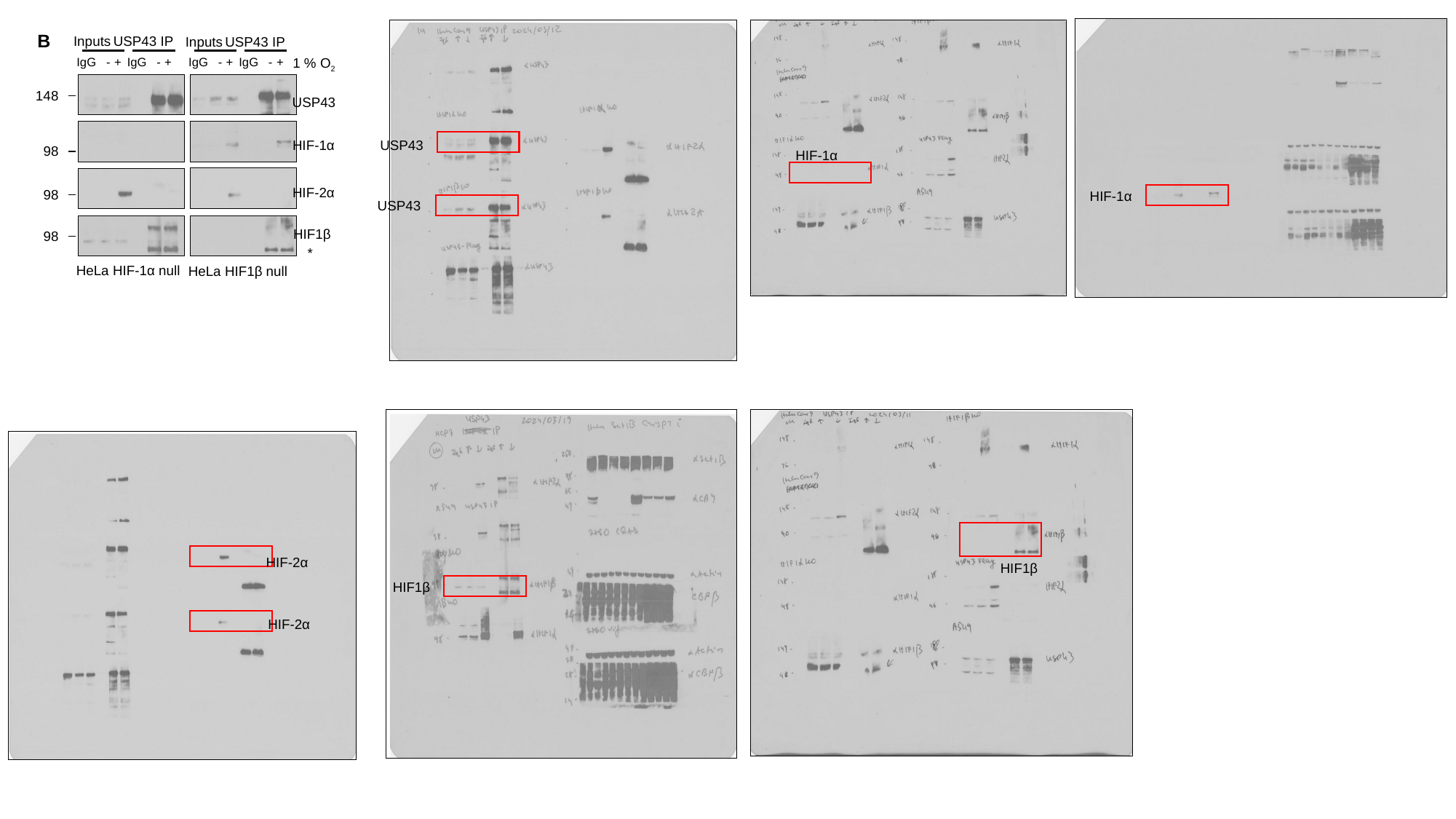

B
Inputs
USP43 IP
Inputs
USP43 IP
IgG
-
+
IgG
-
+
IgG
-
+
IgG
-
+
1 % O2
148
USP43
USP43
HIF-1α
98
HIF-1α
HIF-2α
98
HIF-1α
USP43
HIF1β
98
*
HeLa HIF-1α null
HeLa HIF1β null
HIF-2α
HIF1β
HIF1β
HIF-2α

## Slide 3
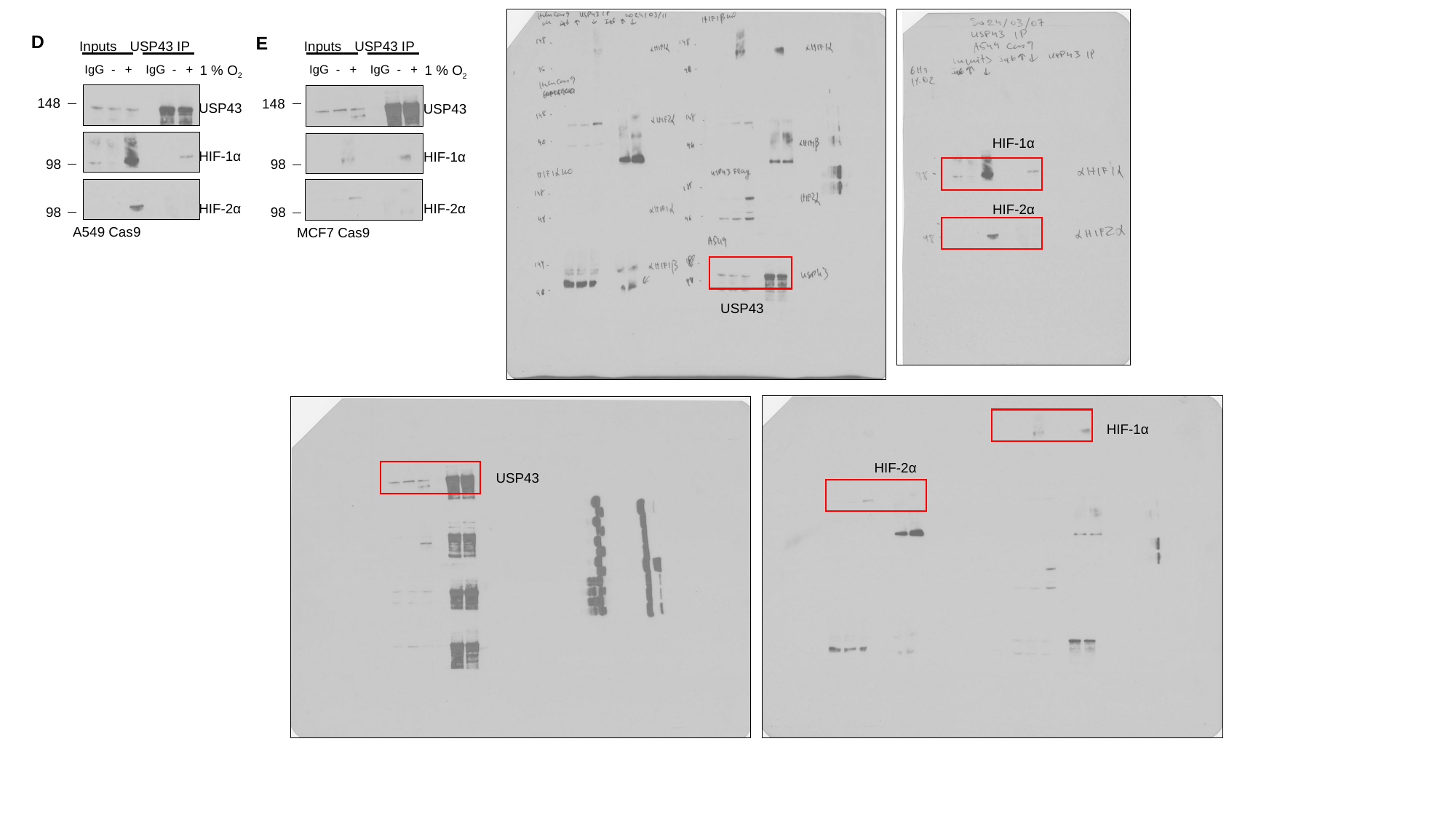

D
E
Inputs
USP43 IP
Inputs
USP43 IP
IgG
-
+
IgG
-
+
1 % O2
1 % O2
IgG
-
+
IgG
-
+
148
148
USP43
USP43
HIF-1α
HIF-1α
HIF-1α
98
98
HIF-2α
HIF-2α
HIF-2α
98
98
A549 Cas9
MCF7 Cas9
USP43
HIF-1α
HIF-2α
USP43

## Slide 4
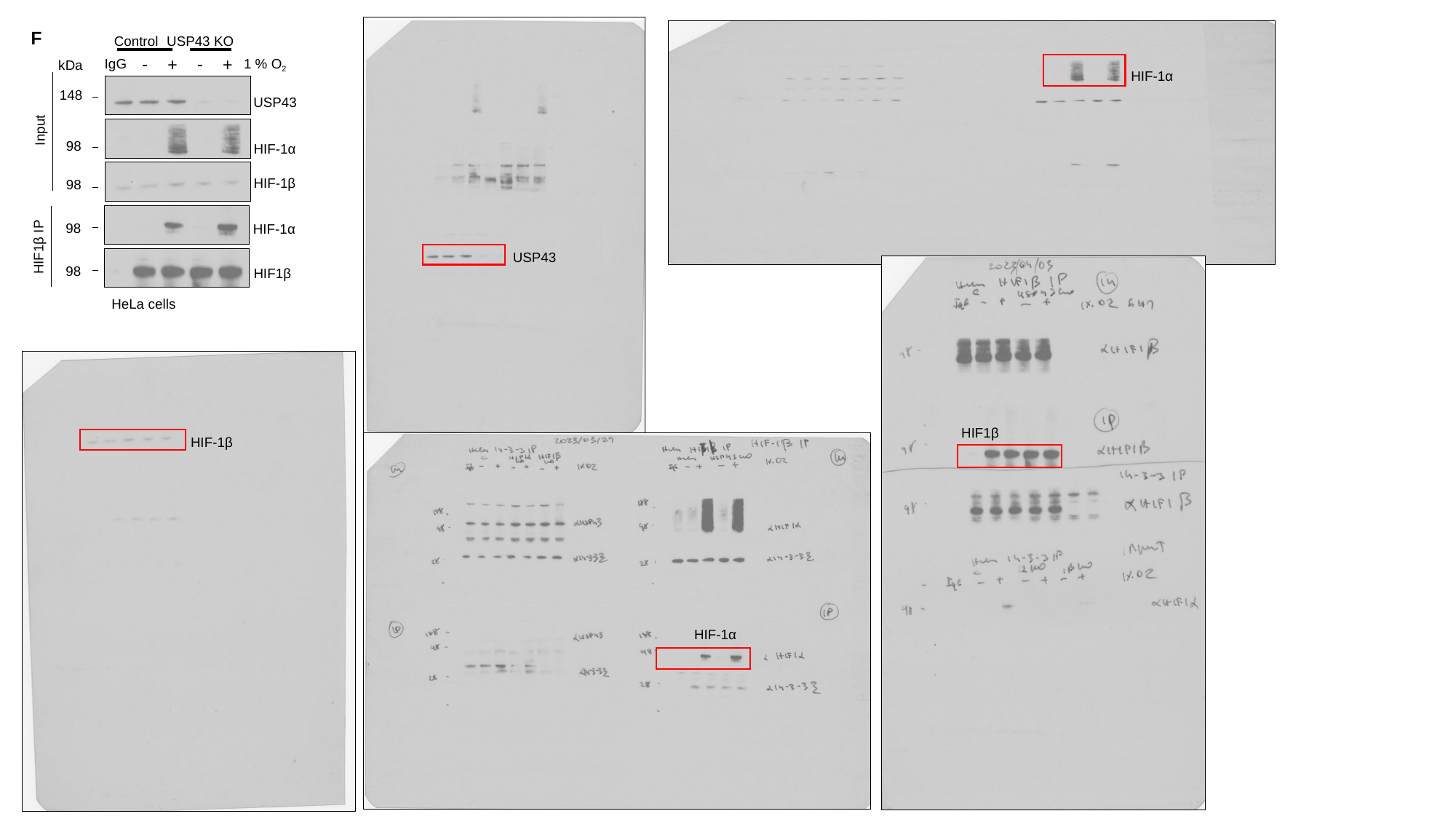

F
Control
USP43 KO
-
+
-
+
1 % O2
IgG
kDa
HIF-1α
148
USP43
Input
98
HIF-1α
HIF-1β
98
98
HIF-1α
HIF1β IP
USP43
98
HIF1β
HeLa cells
HIF1β
HIF-1β
HIF-1α
