# Supplementary material for: Deubiquitinating enzyme mutagenesis screens identify a USP43-dependent HIF-1 transcriptional response
Source: EMBO J. 2024 Jul 15;43(17):8. doi: 10.1038/s44318-024-00166-6 (PMC11377827; doi:10.1038/s44318-024-00166-6)
Supplement: Supplementary file 12 — Extended View and Appendix Source Data [file 44318_2024_166_MOESM12_ESM.zip › Extended View and Appendix Source Data/Figure EV3/EV3 G, I.pptx]

## Slide 1
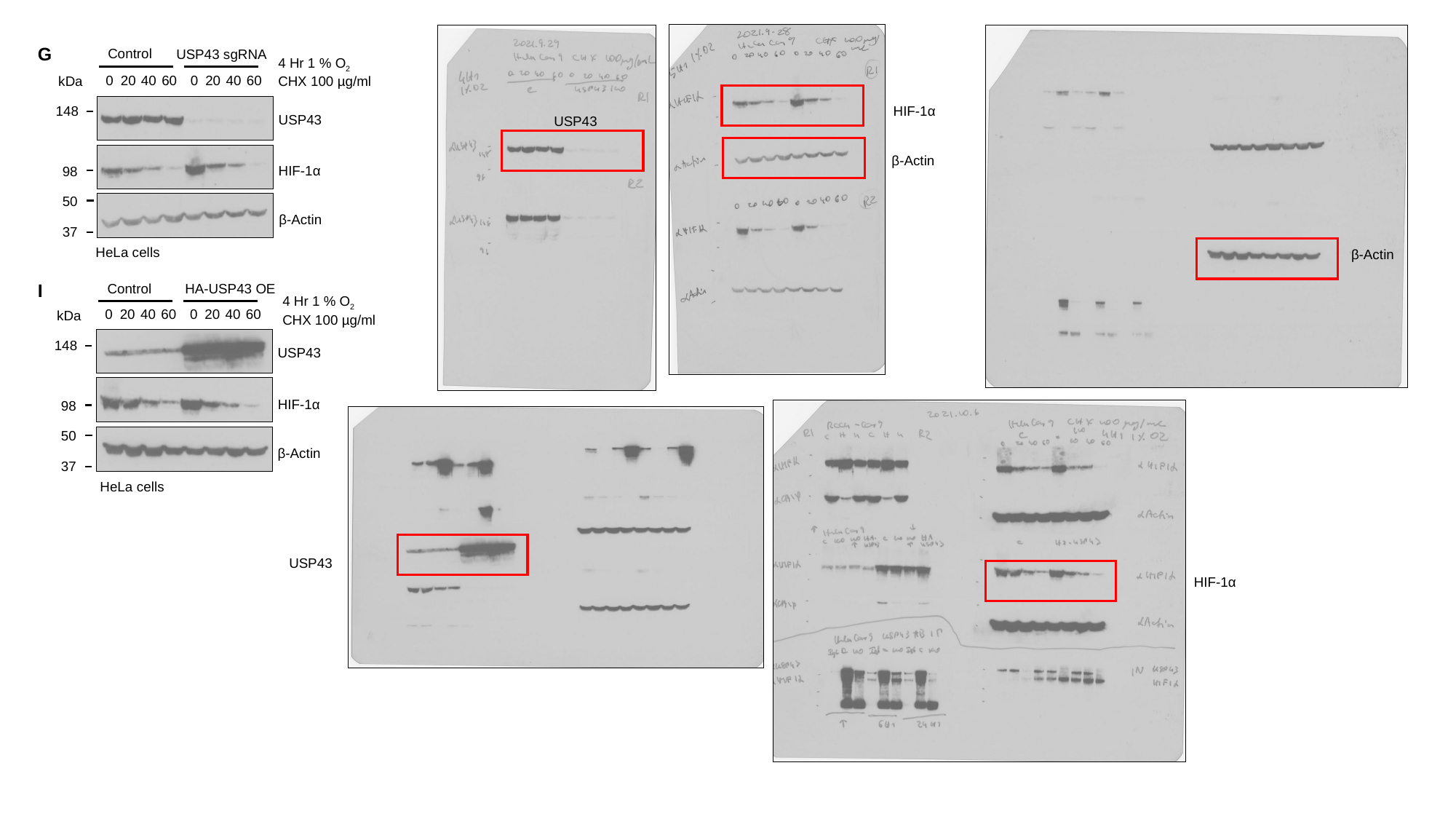

G
Control
USP43 sgRNA
4 Hr 1 % O2
CHX 100 µg/ml
0
20
40
60
0
20
40
60
kDa
HIF-1α
148
USP43
USP43
β-Actin
HIF-1α
98
50
β-Actin
37
HeLa cells
β-Actin
I
Control
HA-USP43 OE
4 Hr 1 % O2
CHX 100 µg/ml
0
20
40
60
0
20
40
60
kDa
148
USP43
HIF-1α
98
50
β-Actin
37
HeLa cells
USP43
HIF-1α
