# Supplementary material for: Deubiquitinating enzyme mutagenesis screens identify a USP43-dependent HIF-1 transcriptional response
Source: EMBO J. 2024 Jul 15;43(17):8. doi: 10.1038/s44318-024-00166-6 (PMC11377827; doi:10.1038/s44318-024-00166-6)
Supplement: Supplementary file 12 — Extended View and Appendix Source Data [file 44318_2024_166_MOESM12_ESM.zip › Extended View and Appendix Source Data/Figure EV5/EV5 A, B, C, D, F, G, I, J, K WB.pptx]

## Slide 1
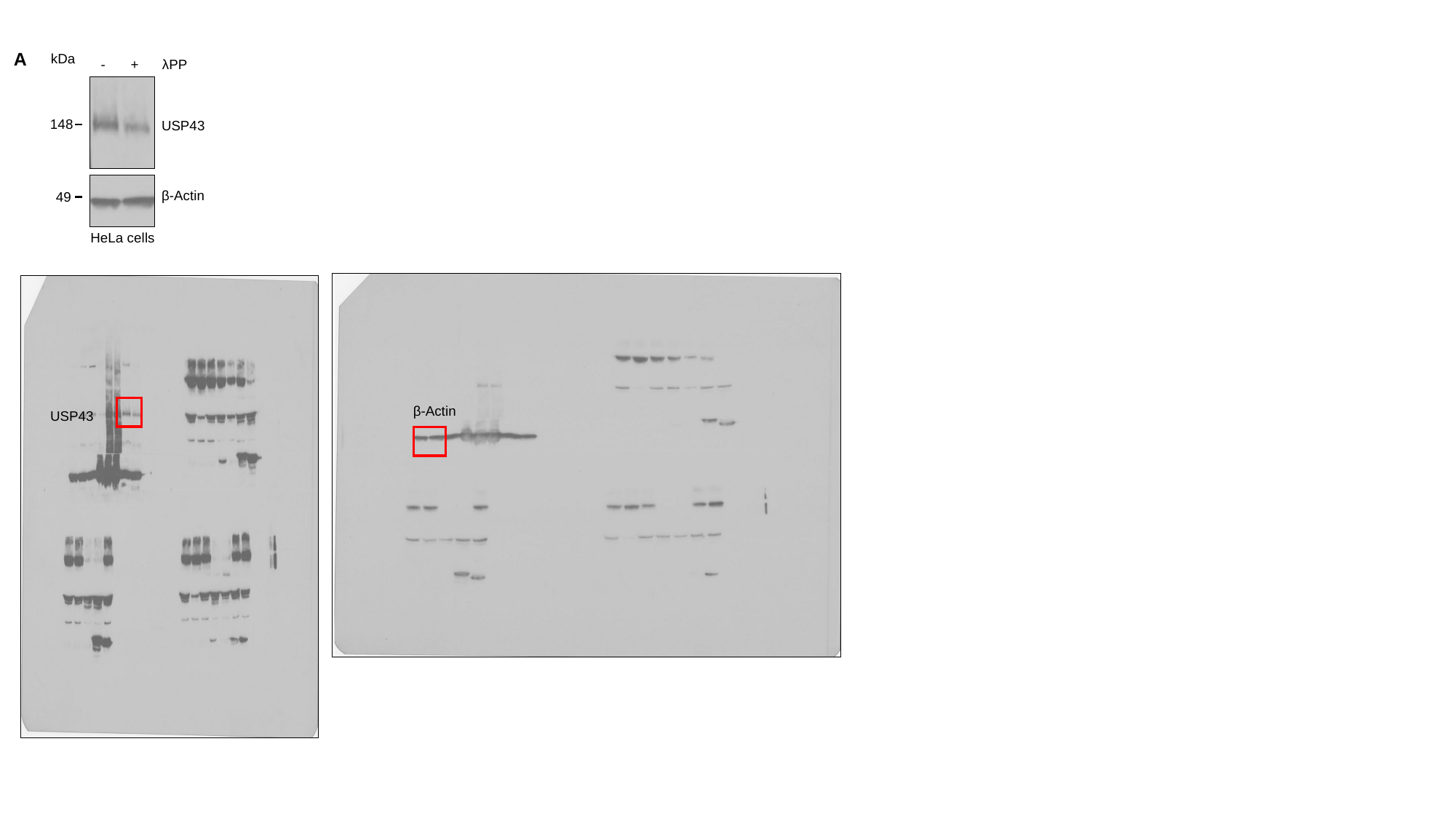

A
kDa
-
+
λPP
148
USP43
β-Actin
49
HeLa cells
β-Actin
USP43

## Slide 2
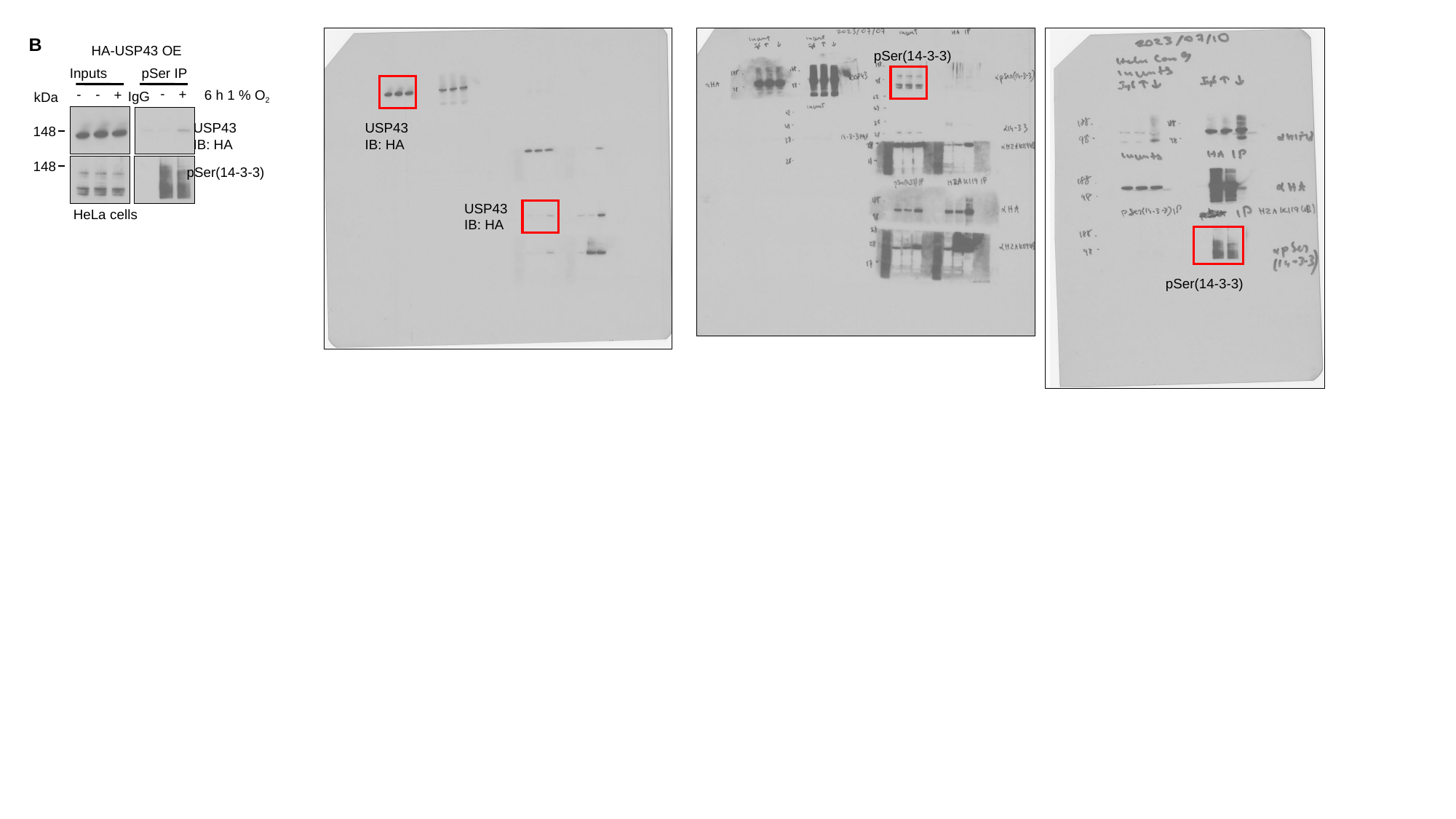

B
HA-USP43 OE
pSer(14-3-3)
pSer IP
Inputs
-
-
-
+
+
6 h 1 % O2
IgG
kDa
USP43
IB: HA
USP43
IB: HA
148
148
pSer(14-3-3)
USP43
IB: HA
HeLa cells
pSer(14-3-3)

## Slide 3
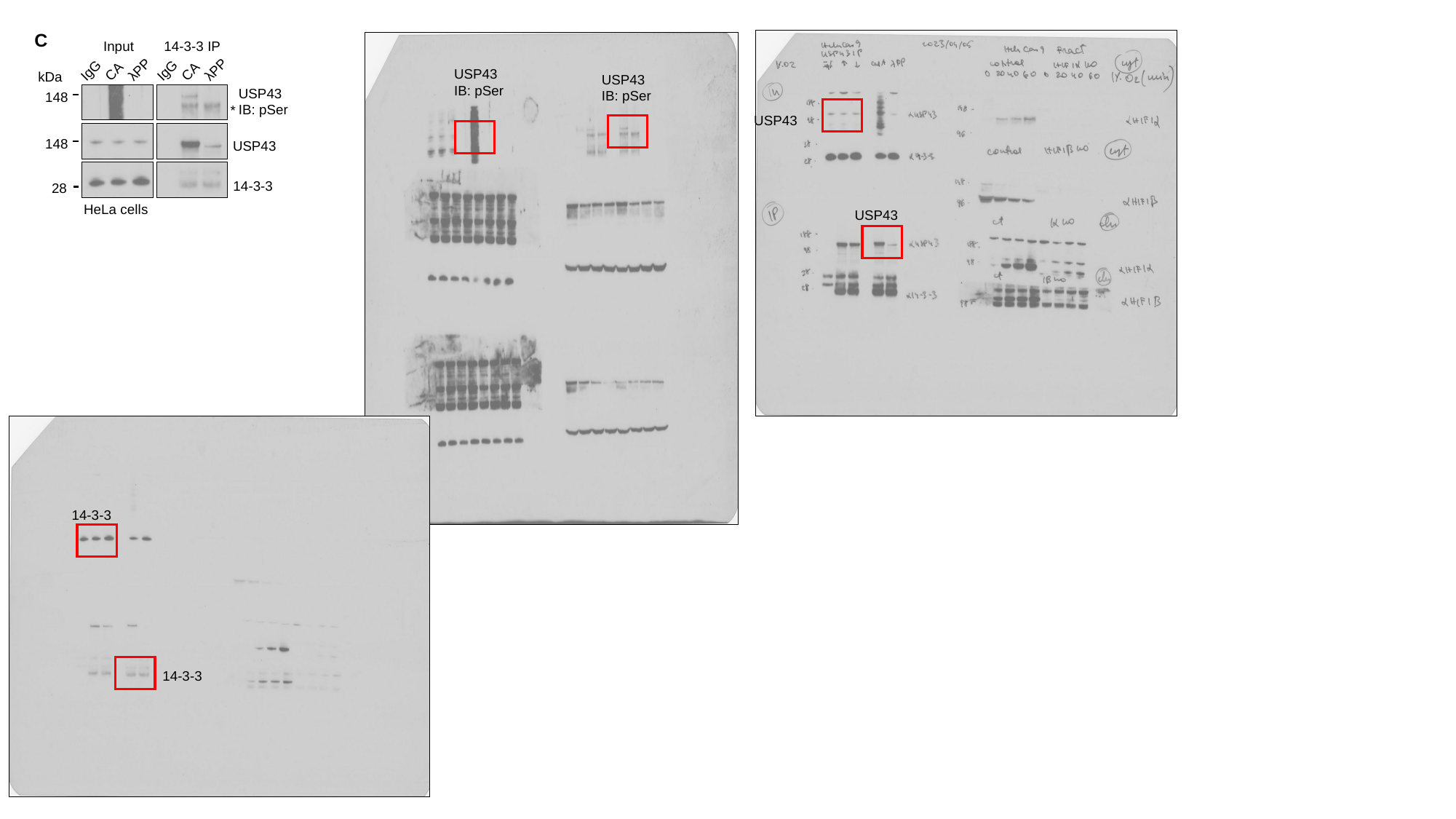

C
Input
14-3-3 IP
λPP
λPP
IgG
IgG
CA
CA
USP43
IB: pSer
kDa
USP43
IB: pSer
USP43
IB: pSer
148
*
USP43
148
USP43
14-3-3
28
HeLa cells
USP43
14-3-3
14-3-3

## Slide 4
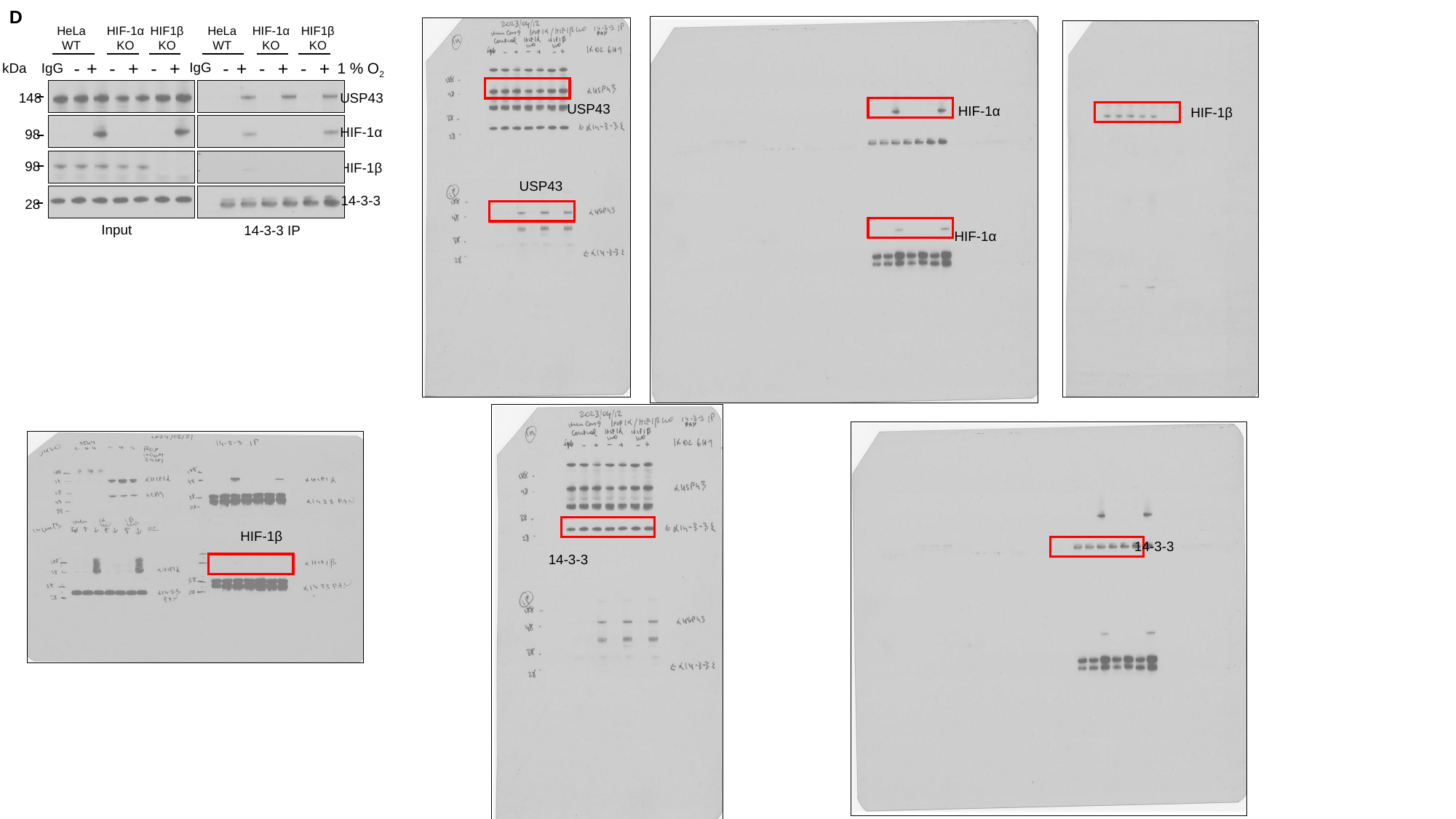

D
HeLa WT
HIF-1α KO
HIF1β KO
HeLa WT
HIF-1α KO
HIF1β KO
-
+
-
+
-
+
-
+
-
+
-
+
1 % O2
IgG
IgG
kDa
148
USP43
USP43
HIF-1α
HIF-1β
HIF-1α
98
98
HIF-1β
USP43
14-3-3
28
Input
14-3-3 IP
HIF-1α
HIF-1β
14-3-3
14-3-3

## Slide 5
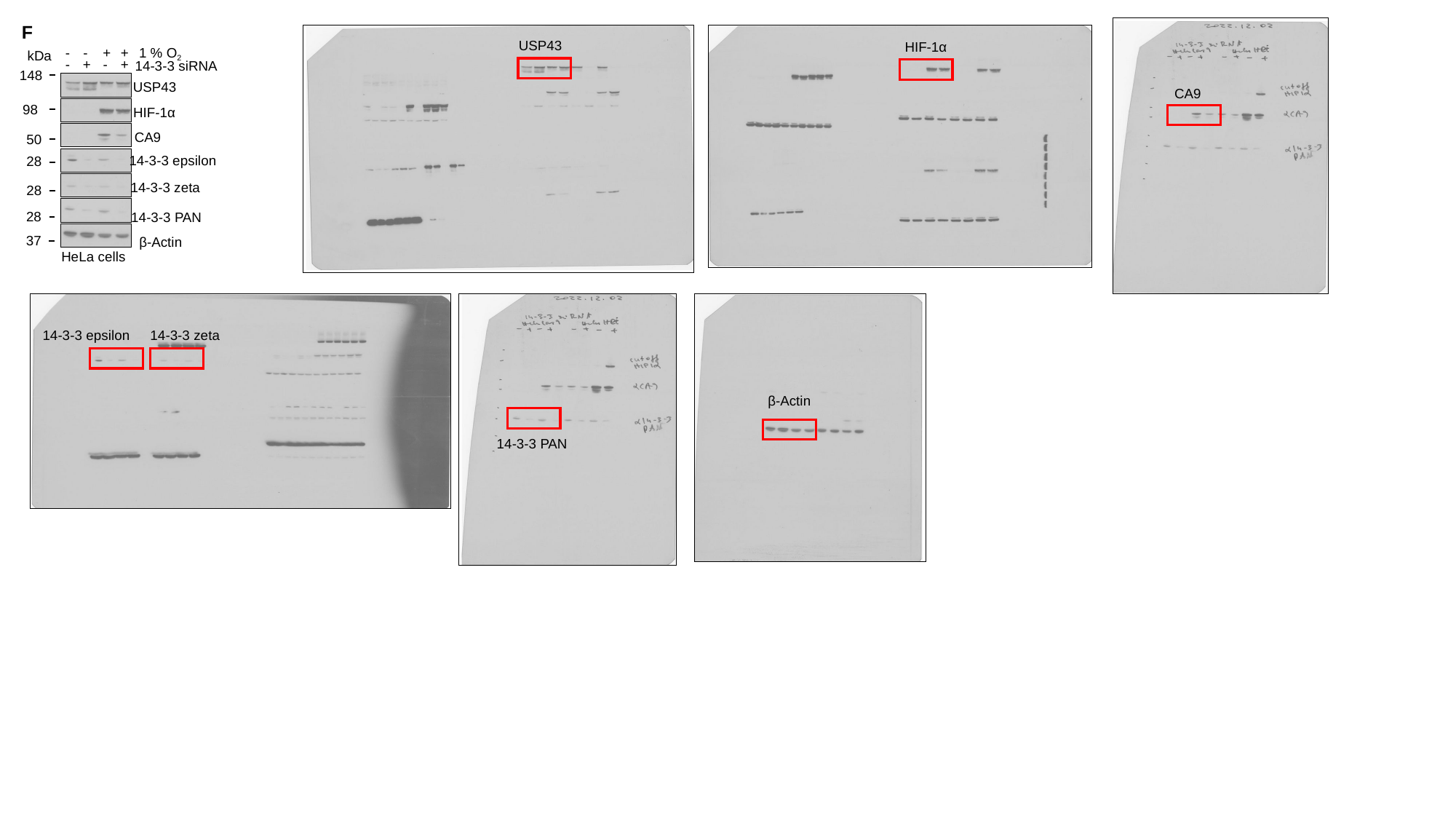

F
USP43
HIF-1α
-
-
+
+
1 % O2
kDa
-
+
-
+
14-3-3 siRNA
148
USP43
CA9
98
HIF-1α
CA9
50
14-3-3 epsilon
28
14-3-3 zeta
28
28
14-3-3 PAN
37
β-Actin
HeLa cells
14-3-3 zeta
14-3-3 epsilon
β-Actin
14-3-3 PAN

## Slide 6
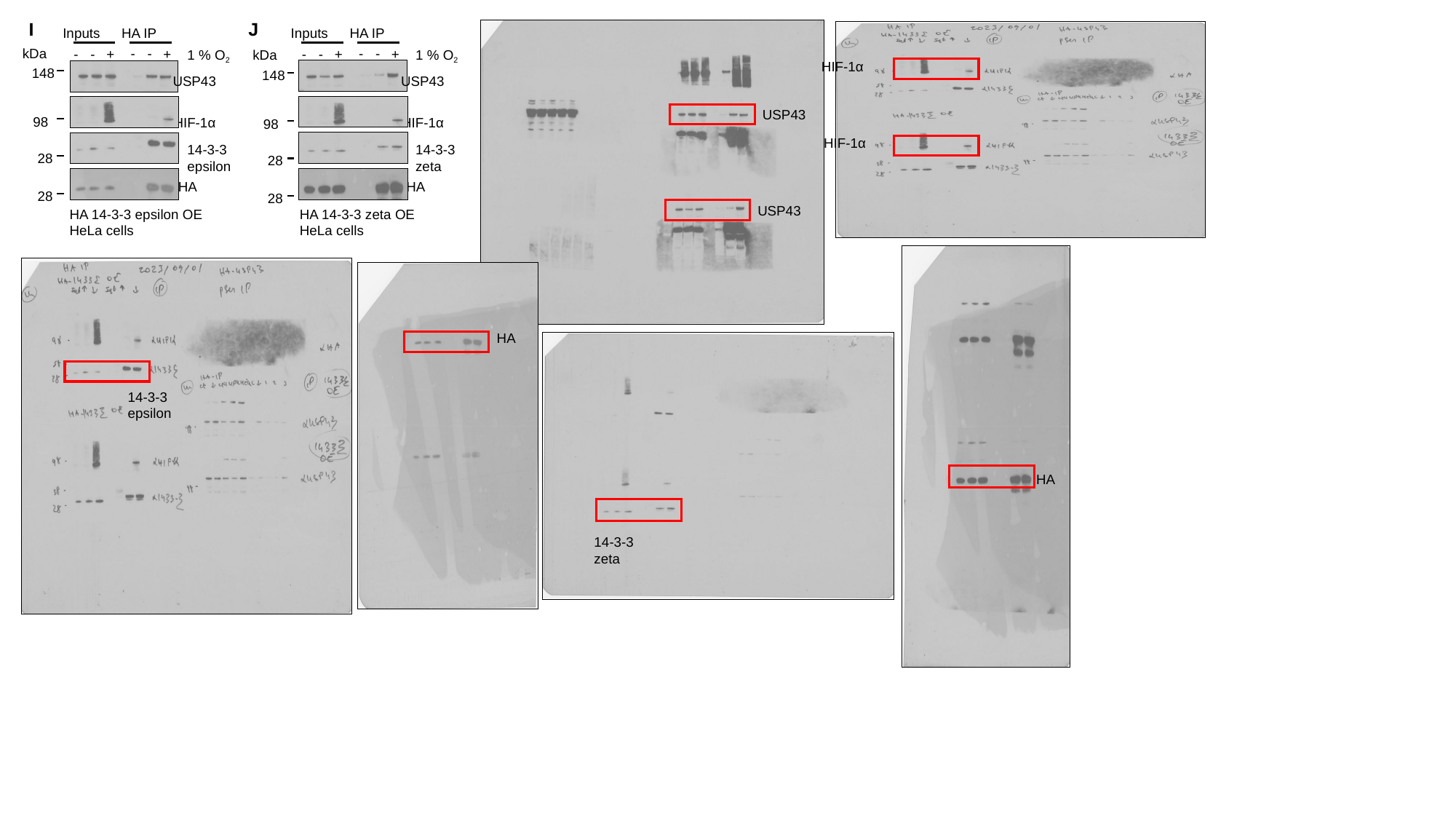

I
J
Inputs
HA IP
Inputs
HA IP
kDa
-
-
-
-
-
-
-
-
+
+
+
+
1 % O2
1 % O2
kDa
HIF-1α
148
148
USP43
USP43
USP43
98
HIF-1α
HIF-1α
98
HIF-1α
14-3-3
epsilon
14-3-3
zeta
28
28
HA
HA
28
28
USP43
HA 14-3-3 epsilon OE
HeLa cells
HA 14-3-3 zeta OE
HeLa cells
HA
14-3-3
epsilon
HA
14-3-3
zeta

## Slide 7
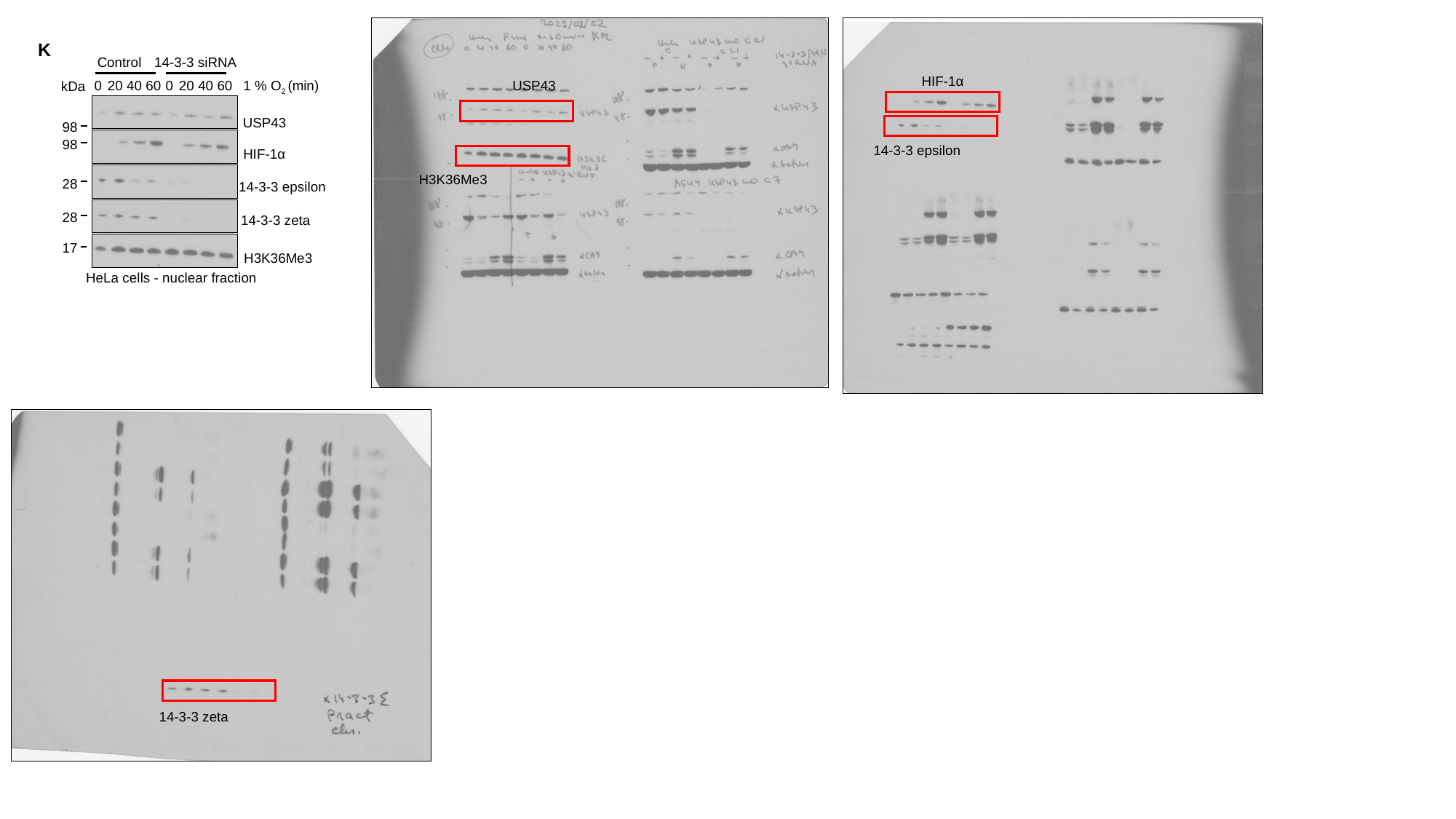

K
14-3-3 siRNA
Control
HIF-1α
USP43
1 % O2 (min)
0
20
40
60
0
20
40
60
kDa
USP43
98
98
14-3-3 epsilon
HIF-1α
H3K36Me3
28
14-3-3 epsilon
28
14-3-3 zeta
17
H3K36Me3
HeLa cells - nuclear fraction
14-3-3 zeta
