# Supplementary material for: Deubiquitinating enzyme mutagenesis screens identify a USP43-dependent HIF-1 transcriptional response
Source: EMBO J. 2024 Jul 15;43(17):8. doi: 10.1038/s44318-024-00166-6 (PMC11377827; doi:10.1038/s44318-024-00166-6)
Supplement: Supplementary file 12 — Extended View and Appendix Source Data [file 44318_2024_166_MOESM12_ESM.zip › Extended View and Appendix Source Data/Appendix Figure S4/S4 A, B WB.pptx]

## Slide 1
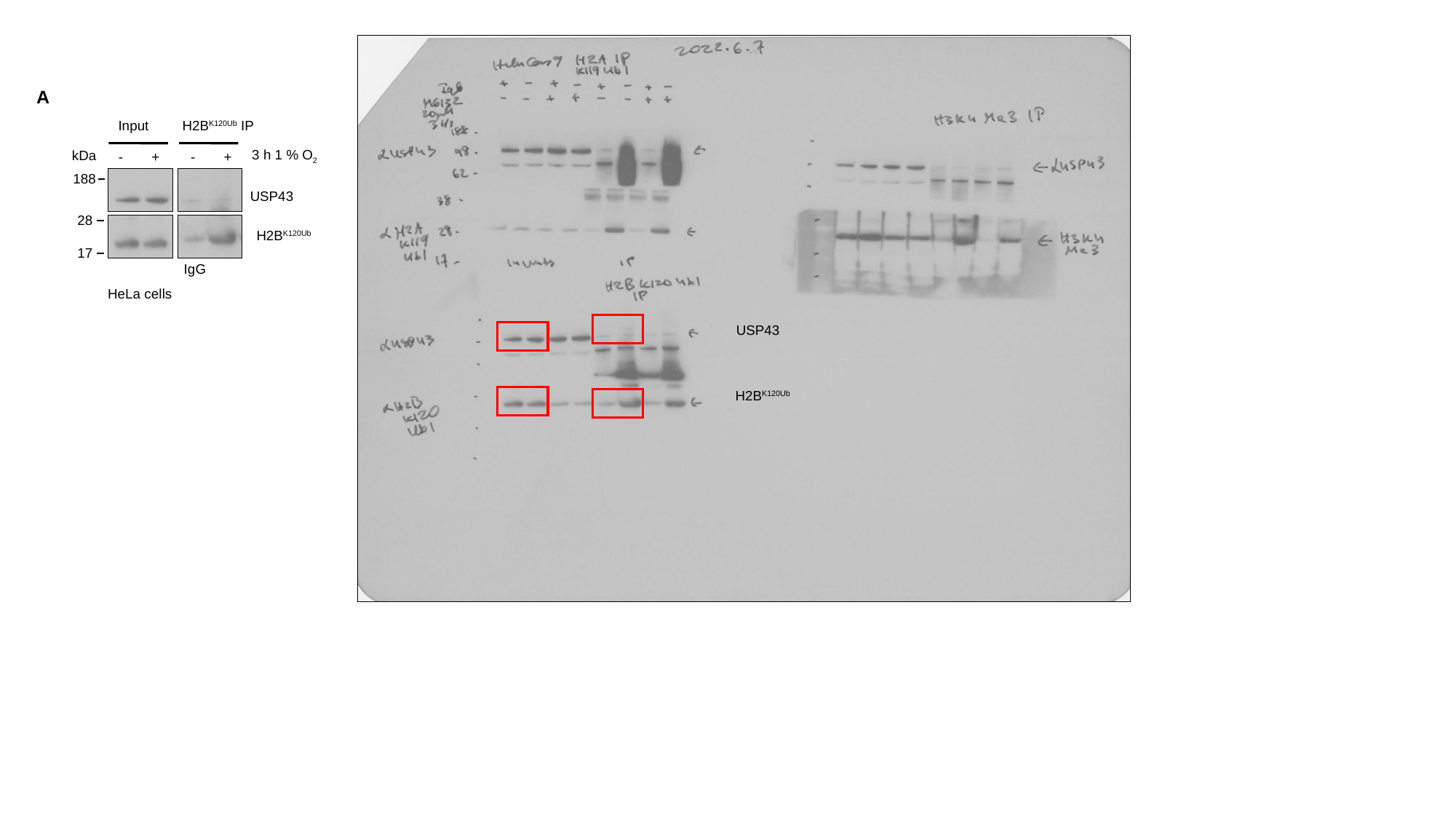

A
Input
H2BK120Ub IP
3 h 1 % O2
kDa
-
+
-
+
188
USP43
28
H2BK120Ub
17
IgG
HeLa cells
USP43
H2BK120Ub

## Slide 2
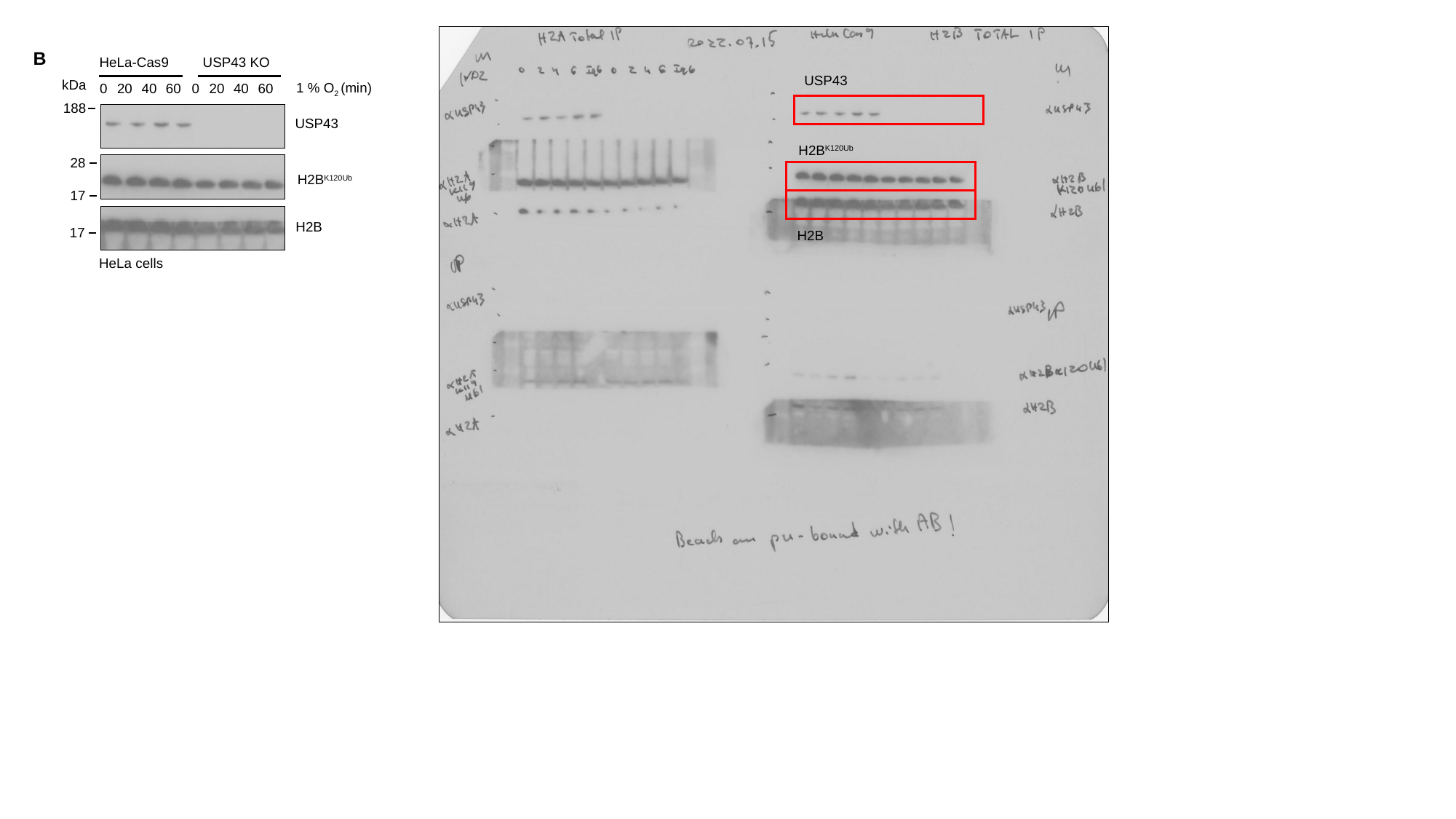

B
HeLa-Cas9
USP43 KO
USP43
kDa
1 % O2 (min)
0
20
40
60
0
20
40
60
188
USP43
H2BK120Ub
28
H2BK120Ub
17
H2B
17
H2B
HeLa cells
