# Supplementary material for: Deubiquitinating enzyme mutagenesis screens identify a USP43-dependent HIF-1 transcriptional response
Source: EMBO J. 2024 Jul 15;43(17):8. doi: 10.1038/s44318-024-00166-6 (PMC11377827; doi:10.1038/s44318-024-00166-6)
Supplement: Supplementary file 12 — Extended View and Appendix Source Data [file 44318_2024_166_MOESM12_ESM.zip › Extended View and Appendix Source Data/Appendix Figure S3/S3 A, C, D WB.pptx]

## Slide 1
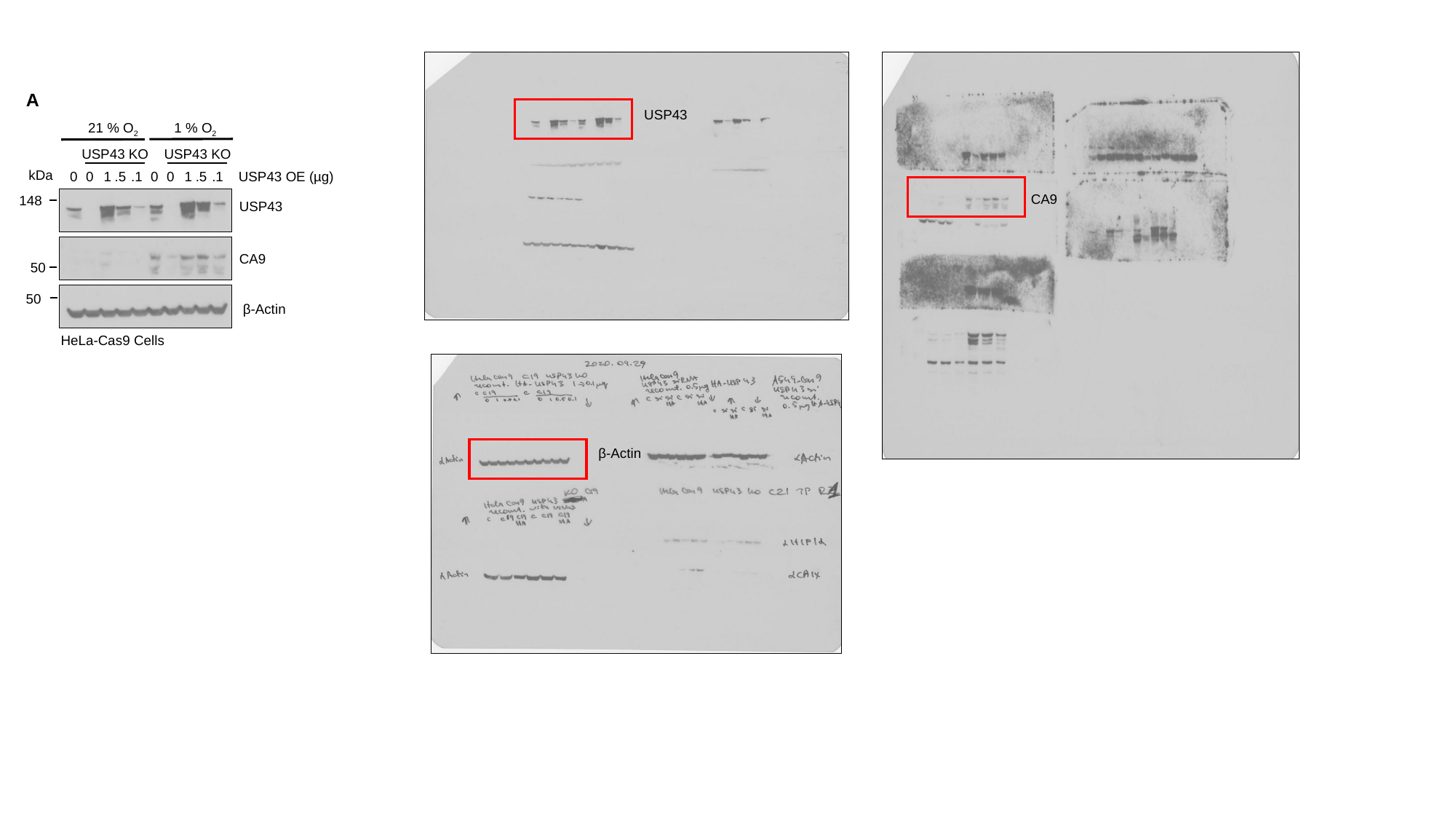

A
USP43
21 % O2
1 % O2
USP43 KO
USP43 KO
kDa
USP43 OE (µg)
0
0
1
.5
.1
0
0
1
.5
.1
CA9
148
USP43
CA9
50
50
β-Actin
HeLa-Cas9 Cells
β-Actin

## Slide 2
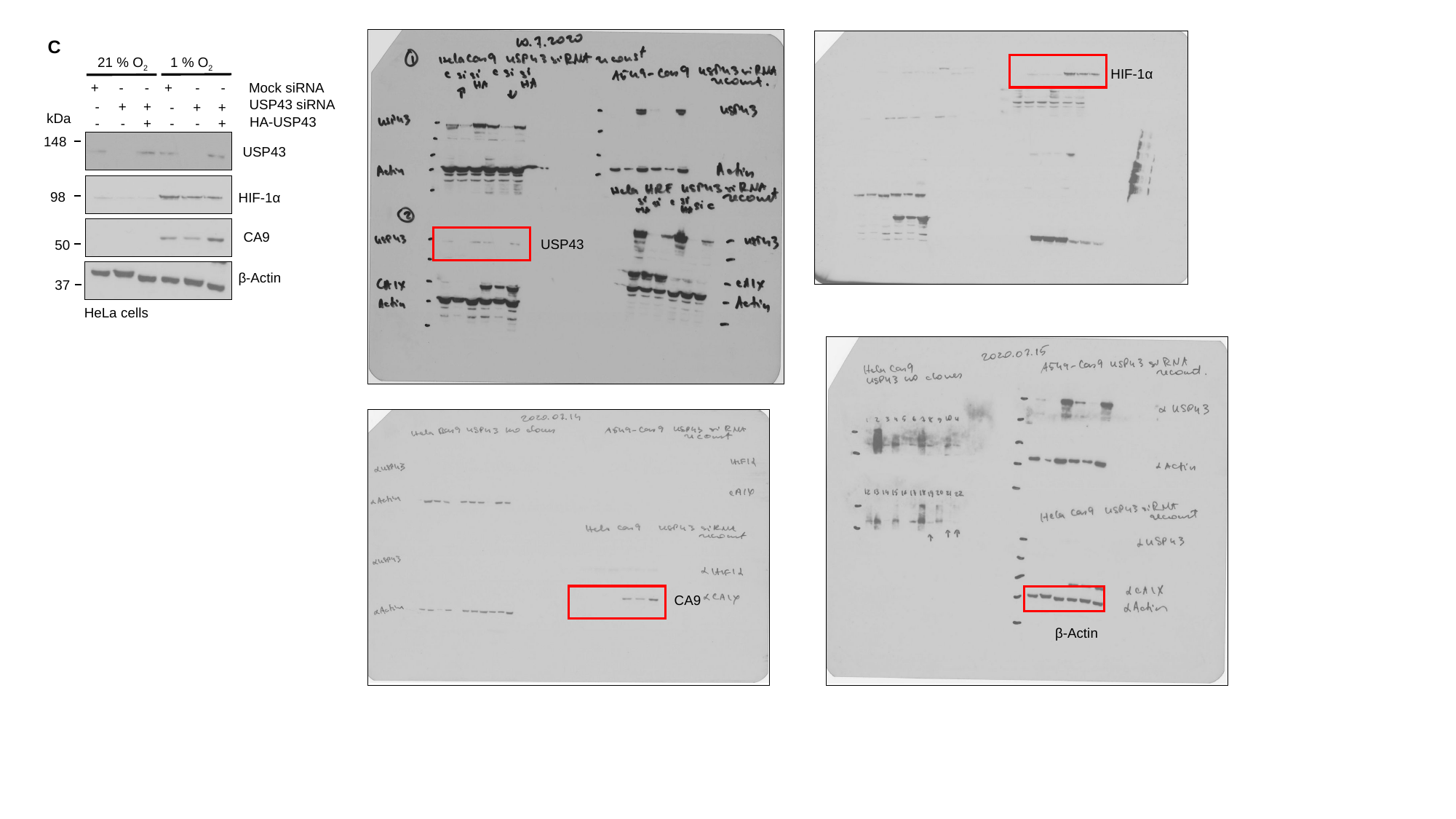

C
1 % O2
21 % O2
HIF-1α
+
-
-
+
-
-
Mock siRNA
USP43 siRNA
-
+
+
-
+
+
kDa
HA-USP43
-
-
+
-
-
+
148
USP43
98
HIF-1α
CA9
USP43
50
β-Actin
37
HeLa cells
CA9
β-Actin

## Slide 3
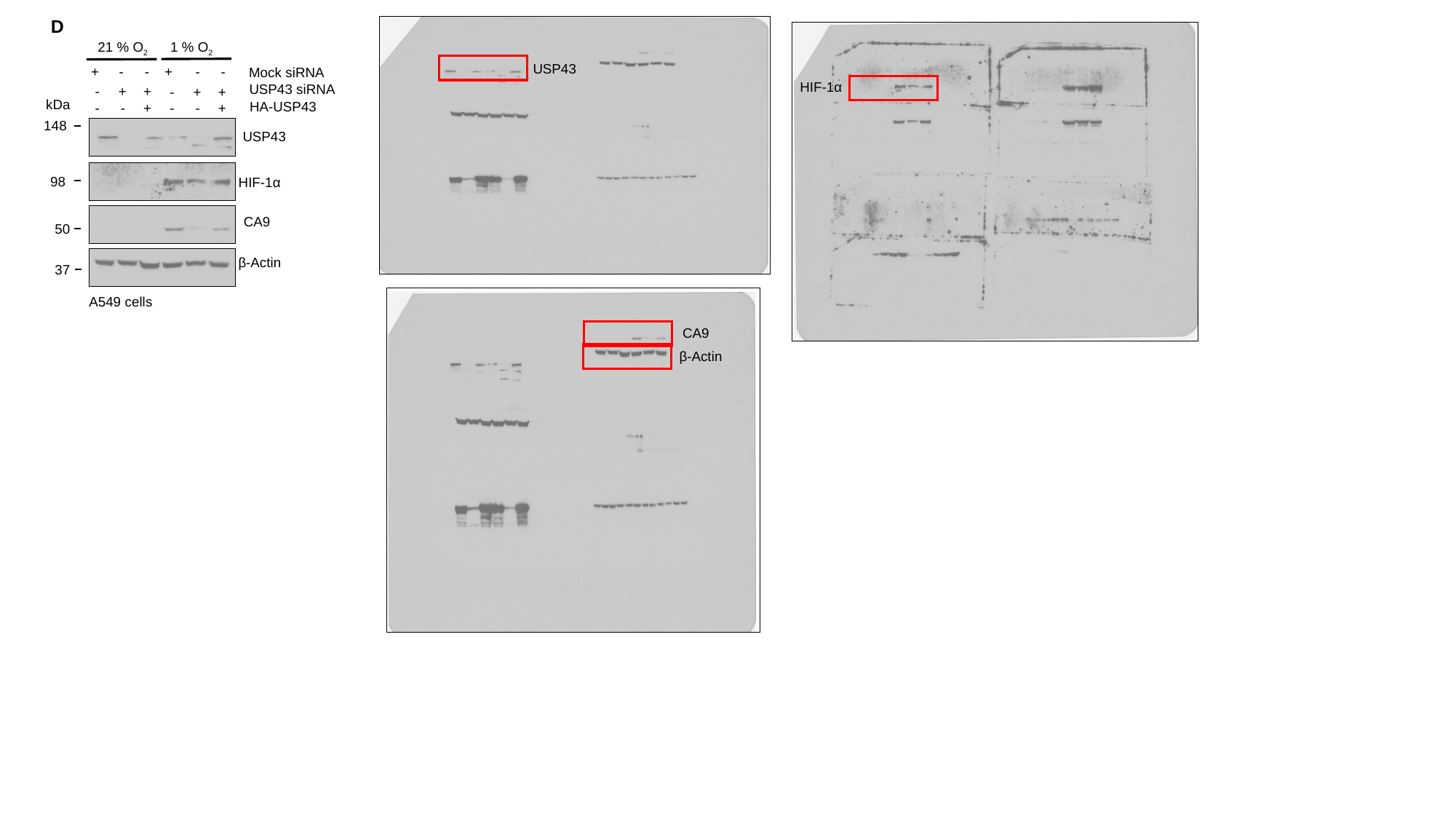

D
1 % O2
21 % O2
USP43
+
-
-
+
-
-
Mock siRNA
HIF-1α
USP43 siRNA
-
+
+
-
+
+
kDa
HA-USP43
-
-
+
-
-
+
148
USP43
98
HIF-1α
CA9
50
β-Actin
37
A549 cells
CA9
β-Actin
