# Supplementary material for: Deubiquitinating enzyme mutagenesis screens identify a USP43-dependent HIF-1 transcriptional response
Source: EMBO J. 2024 Jul 15;43(17):8. doi: 10.1038/s44318-024-00166-6 (PMC11377827; doi:10.1038/s44318-024-00166-6)
Supplement: Supplementary file 12 — Extended View and Appendix Source Data [file 44318_2024_166_MOESM12_ESM.zip › Extended View and Appendix Source Data/Appendix Figure S2/S2 A, C WB.pptx]

## Slide 1
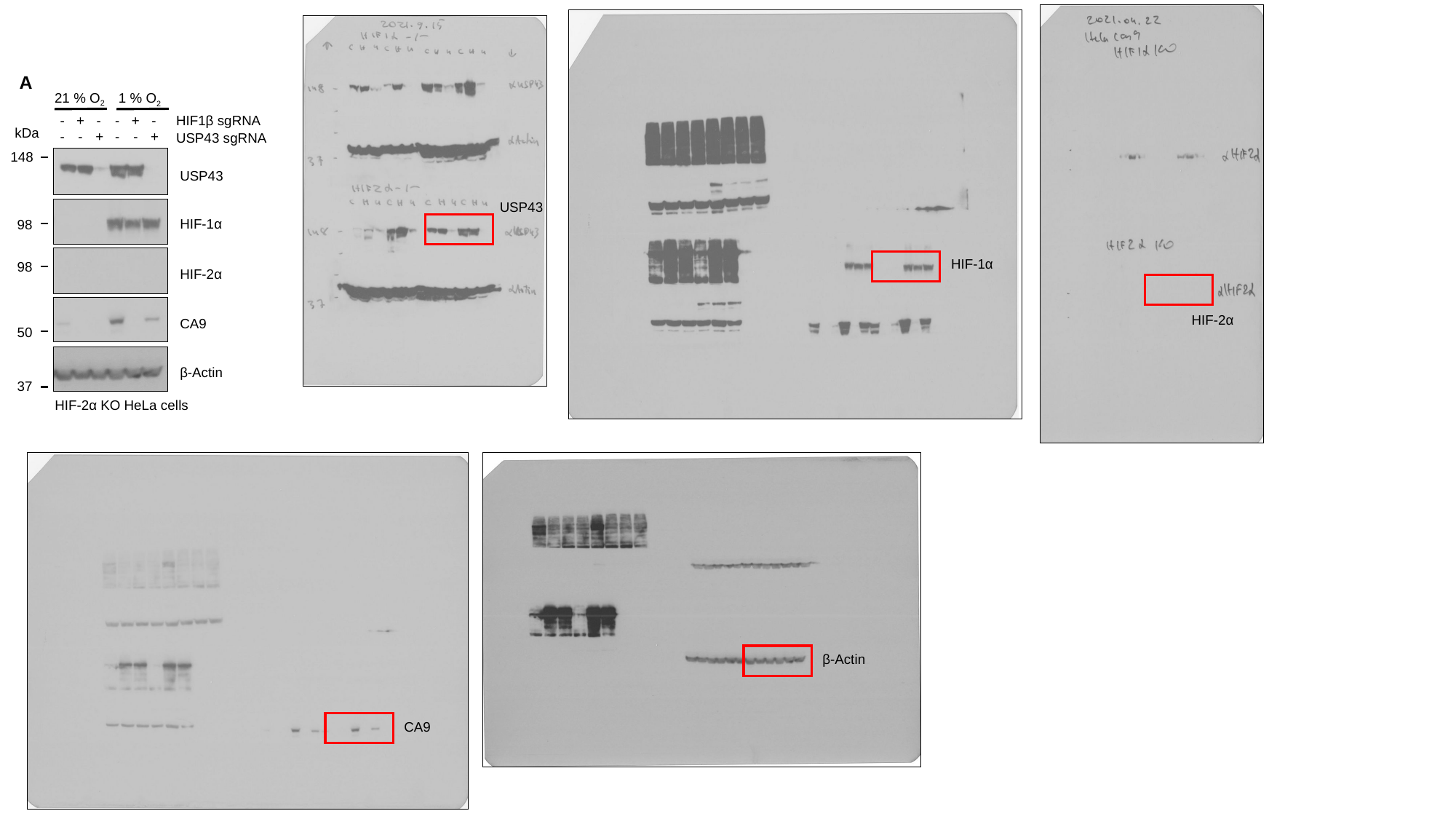

A
21 % O2
1 % O2
-
+
-
-
+
-
-
-
+
-
-
+
HIF1β sgRNA
USP43 sgRNA
kDa
148
USP43
USP43
HIF-1α
98
HIF-1α
98
HIF-2α
HIF-2α
CA9
50
β-Actin
37
HIF-2α KO HeLa cells
β-Actin
CA9

## Slide 2
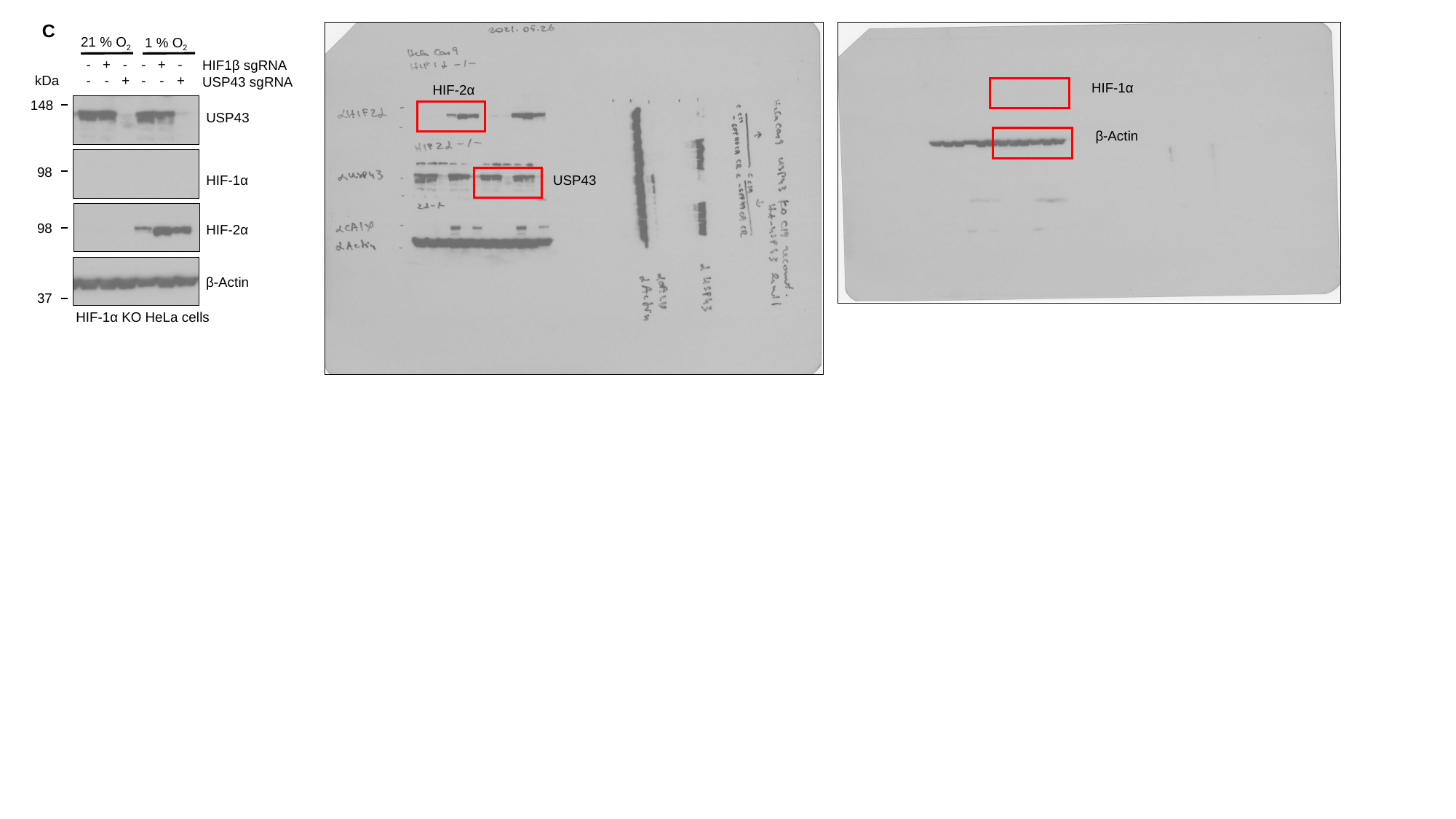

C
21 % O2
1 % O2
-
+
-
-
+
-
-
-
+
-
-
+
HIF1β sgRNA
kDa
USP43 sgRNA
HIF-1α
HIF-2α
148
USP43
β-Actin
98
HIF-1α
USP43
98
HIF-2α
β-Actin
37
HIF-1α KO HeLa cells
