# Supplementary material for: Deubiquitinating enzyme mutagenesis screens identify a USP43-dependent HIF-1 transcriptional response
Source: EMBO J. 2024 Jul 15;43(17):8. doi: 10.1038/s44318-024-00166-6 (PMC11377827; doi:10.1038/s44318-024-00166-6)
Supplement: Supplementary file 13 — Expanded View Figures [file 44318_2024_166_MOESM13_ESM.pdf]

Expanded View Figures

Figure EV1. Validation of DUBs identified as regulators of the HIF response.

(**A**) Mixed KO populations of USP43 or USP52 HeLa HRE-^{ODD}GFP reporter cells were generated by lentiviral transduction with sgRNA. Cells were then incubated in 1% oxygen for 24 h and analysed by flow cytometry. Representative of three biological replicates. (**B**) HeLa HRE-^{ODD}GFP reporter cells transduced with three different sgRNAs against OTUD5 cultured in 21% oxygen. Representative of three biological replicates. (**C–H**) Schematic of the HIF activator validation screen (**C**). The top ten hits by log2 fold change (**H**) were validated in HKC-8 and RPE-1 cells. The cells were transduced with two sgRNAs per DUB. After 8 days, cells were incubated in 1% oxygen for 24 h and analysed by flow cytometry for endogenous cell surface CA9 (**D**, **F**) and intracellular HIF-1α levels (**E**, **G**). DUBs that were validated in either HKC-8, RPE-1 or both cells are highlighted in red (**H**). $n = 4$ biologically independent samples for CA9 levels, and $n = 3$ for HIF-1α levels, mean ± sd. (**I**) Principal component analysis (PCA) plot of RNA-seq of HeLa control, HIF1β KO and USP43 KO cells that were treated with 21 or 1% oxygen (O_2) for 16 h before RNA was extracted and sequenced using Hiseq. $n = 3$ biologically independent samples, mean ± sd. (**J**) Quantitative RT-PCR (qPCR) of *USP43* mRNA in A549, MCF7 or HKC-8 cells. Cells were incubated in 1 or 21% oxygen for 16 h. $n = 3$ biologically independent samples. Mean ± sd. $^{**}P = 0.005$, unpaired *t*-test.

© The Author(s)

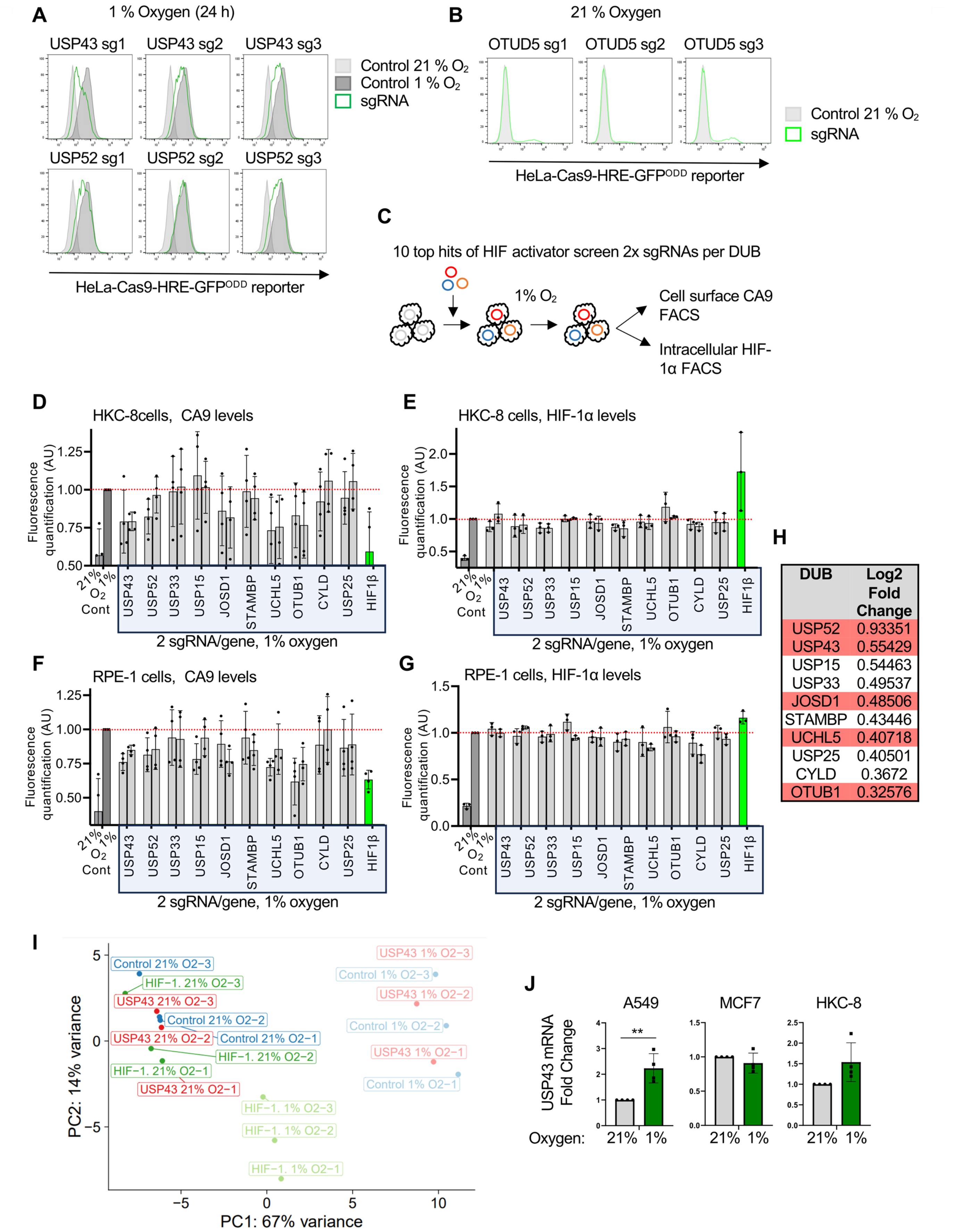

DUB	Log2 Fold Change
USP52	0.93351
USP43	0.55429
USP15	0.54463
USP33	0.49537
JOSD1	0.48506
STAMBP	0.43446
UCHL5	0.40718
USP25	0.40501
CYLD	0.3672
OTUB1	0.32576

 © The Author(s)

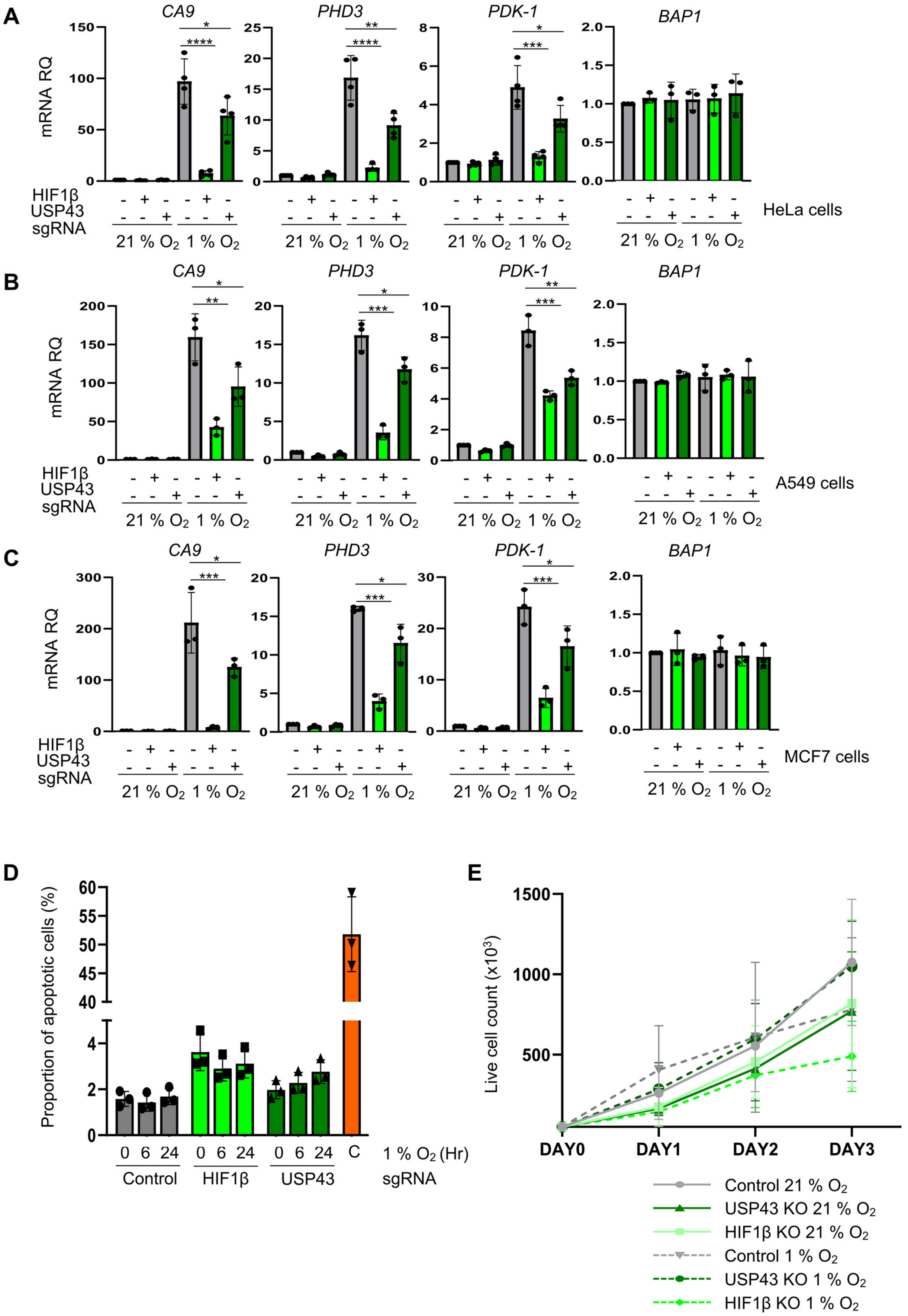
A
CA9
PHD3
PDK-1
BAP1
mRNA RQ
HIF1β
USP43
sgRNA
21 % O2 1 % O2
HeLa cells
B
A549 cells
C
MCF7 cells
D
Proportion of apoptotic cells (%)
0 6 24 0 6 24 0 6 24 C
1 % O2 (Hr)
Control HIF1β USP43
sgRNA
E
Live cell count (x10^3)
DAY0 DAY1 DAY2 DAY3
Control 21 % O2
USP43 KO 21 % O2
HIF1β KO 21 % O2
Control 1 % O2
USP43 KO 1 % O2
HIF1β KO 1 % O2

Figure EV2. USP43 depletion decreases the expression of selected HIF target genes in hypoxia.

(**A–C**) RT-qPCR of *CA9*, *PHD3*, *PDK1*, and *BAP1* in HeLa (**A**), A549 (**B**) and MCF7 (**C**) cells with or without depletion of HIF1β or USP43. Cells were incubated in 21 or 1% oxygen for 16 h prior to lysis. $n = 4$ biologically independent samples, mean ± sd. (**A**) *CA9*: control vs. HIF1β sgRNA 1% O_2 ****$P < 0.0001$, control vs. USP43 sgRNA 1% O_2 *$P = 0.0363$. *PHD3*: control vs. HIF1β sgRNA 1% O_2 ****$P < 0.0001$, control vs. USP43 sgRNA 1% O_2 **$P = 0.0026$. *PDK-1*: control vs. HIF1β sgRNA 1% O_2 ***$P = 0.0002$, control vs. USP43 sgRNA 1% O_2 *$P = 0.0299$. One-way ANOVA. (**B**) $n = 3$ biologically independent samples, mean ± sd. *CA9*: control vs. HIF1β sgRNA 1% O_2 **$P = 0.0018$, control vs. USP43 sgRNA 1% O_2 *$P = 0.0295$. *PHD3*: control vs. HIF1β sgRNA 1% O_2 ***$P = 0.0001$, control vs. USP43 sgRNA 1% O_2 *$P = 0.0233$; PDK-1: control vs. HIF1β sgRNA 1% O_2 ***$P = 0.0004$, control vs. USP43 sgRNA 1% O_2 **$P = 0.0023$. One-way ANOVA. (**C**) $n = 3$ biologically independent samples, mean ± sd. *CA9*: control vs. HIF1β sgRNA 1% O_2 ***$P = 0.0008$, control vs. USP43 sgRNA 1% O_2 *$P = 0.0433$. *PHD3*: control vs. HIF1β sgRNA 1% O_2 ***$P = 0.0001$, control vs. USP43 sgRNA 1% O_2 *$P = 0.0209$. *PDK-1*: control vs. HIF1β sgRNA 1% O_2 ***$P = 0.0009$, control vs. USP43 sgRNA 1% O_2 *$P = 0.0454$. One-way ANOVA. (**D**) Apoptosis assay. HeLa control (grey), HIF1β depleted (light green) or USP43 depleted (dark green) cells were incubated in 1% oxygen for 0, 6 or 24 h, and apoptosis was measured using CellEvent™ Caspase-3/7 Green Flow Cytometry Assay Kit (C10427). Camptothecin (**C**) (100 μM, 24 h) was used as a positive control. $n = 3$ biological replicates, mean ± sd. (**E**) Cell growth assay. HeLa control (grey), HIF1 depleted (light green) or USP43 depleted (dark green) cells were incubated in 21 (solid line) or 1% (dotted line) oxygen for 0, 24, 48 and 72 h and counted at each time point. The cell growth plot represents three biologically independent experiments, mean ± sd.

 © The Author(s)

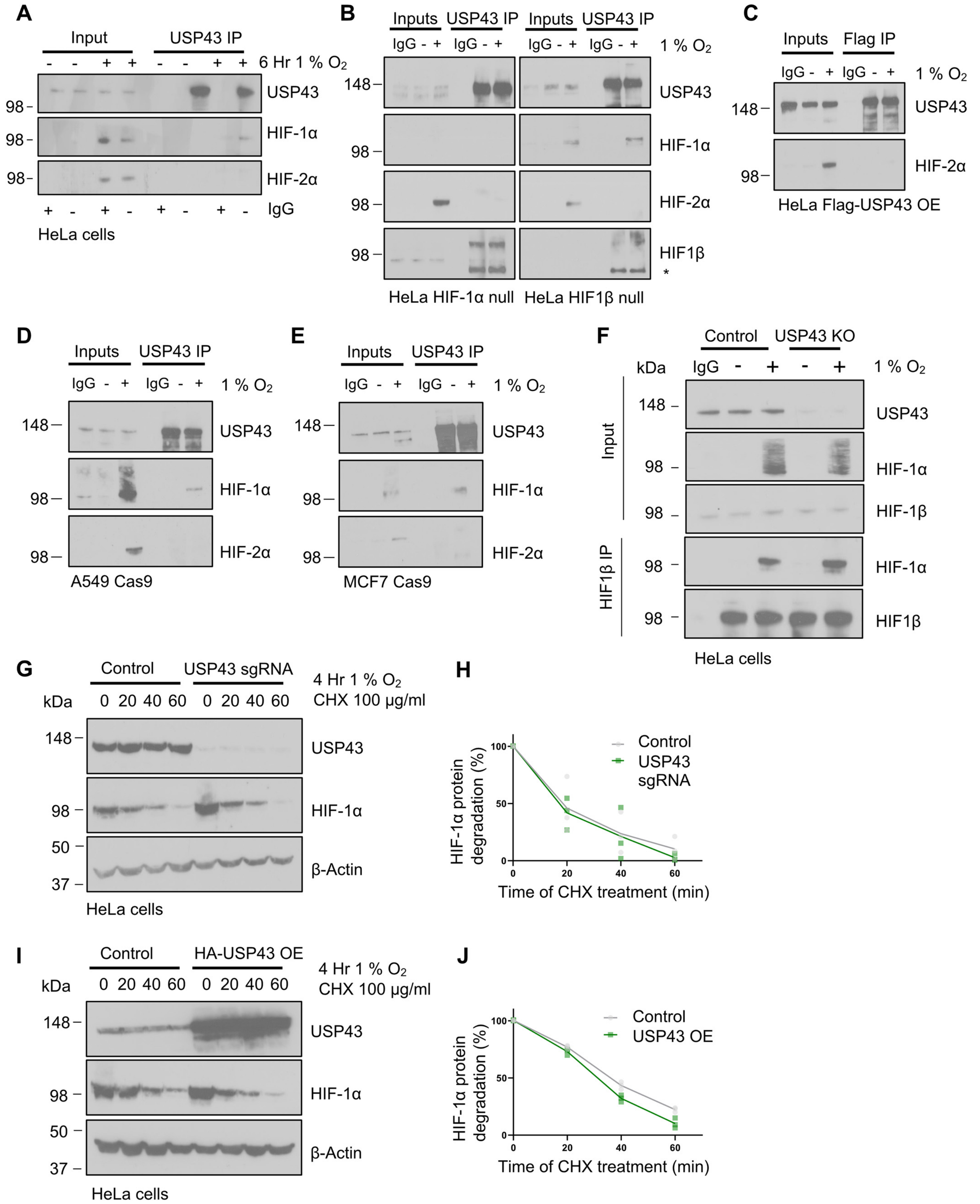
A
Input
USP43 IP
6 Hr 1 % O2
USP43
HIF-1α
HIF-2α
IgG
HeLa cells
B
Inputs USP43 IP Inputs USP43 IP
1 % O2
USP43
HIF-1α
HIF-2α
HIF1β
HeLa HIF-1α null HeLa HIF1β null
C
Inputs Flag IP
1 % O2
USP43
HIF-2α
HeLa Flag-USP43 OE
D
Inputs USP43 IP
1 % O2
USP43
HIF-1α
HIF-2α
A549 Cas9
E
Inputs USP43 IP
1 % O2
USP43
HIF-1α
HIF-2α
MCF7 Cas9
F
Control USP43 KO
kDa
1 % O2
Input
HIF1β IP
USP43
HIF-1α
HIF-1β
HIF1β
HeLa cells
G
Control USP43 sgRNA
4 Hr 1 % O2
CHX 100 μg/ml
kDa
0 20 40 60 0 20 40 60
USP43
HIF-1α
β-Actin
HeLa cells
H
HIF-1α protein degradation (%)
Control
USP43 sgRNA
Time of CHX treatment (min)
I
Control HA-USP43 OE
4 Hr 1 % O2
CHX 100 μg/ml
kDa
0 20 40 60 0 20 40 60
USP43
HIF-1α
β-Actin
HeLa cells
J
HIF-1α protein degradation (%)
Control
USP43 OE
Time of CHX treatment (min)

Figure EV3. USP43 interacts with HIF-1α but not HIF-2α.

(**A**) Endogenous USP43 was immunoprecipitated in HeLa cells grown in 21 or 1% oxygen for 6 h. Samples were immunoblotted for HIF-1α and HIF-2α. Representative of three biological replicates. (**B**) Endogenous USP43 was immunoprecipitated in HIF-1α or HIF1β clonal KO HeLa cells grown in 21 or 1% oxygen for 6 h. Samples were immunoblotted for HIF-1α, HIF-2α and HIF1β. Representative of three biological replicates. (**C**) USP43-Flag was immunoprecipitated in USP43-Flag overexpressing HeLa cells grown in 21 or 1% oxygen for 6 h. Samples were immunoblotted for HIF-2α. Representative of three biological replicates. (**D**, **E**) Endogenous USP43 was immunoprecipitated in A549 (**D**) or MCF7 (**E**) cells grown in 21 or 1% oxygen for 6 h. Samples were immunoblotted for HIF-1α and HIF-2α. Representative of three biological replicates. (**F**) Endogenous HIF1β was immunoprecipitated in HeLa and USP43 depleted cells grown in 21 or 1% oxygen for 6 h. Samples were immunoblotted for HIF-1α. Representative of three biological replicates. (**G**, **H**) Control or mixed population USP43 KO HeLa cells were incubated in 1% oxygen for 4 h and then treated with cycloheximide (100 μg/ml) in hypoxia for 0–60 min. HIF-1α levels were measured by immunoblot (**G**) and quantified by densitometry using ImageJ ($n = 3$) (**H**). (**I**, **J**) Control or USP43 overexpressing (OE) HeLa cells were incubated in 1% oxygen for 4 h and then treated with cycloheximide (100 μg/ml) in hypoxia for 0–60 min. HIF-1α levels were measured by immunoblot (**I**) and quantified by densitometry using ImageJ ($n = 3$) (**J**).

 © The Author(s)

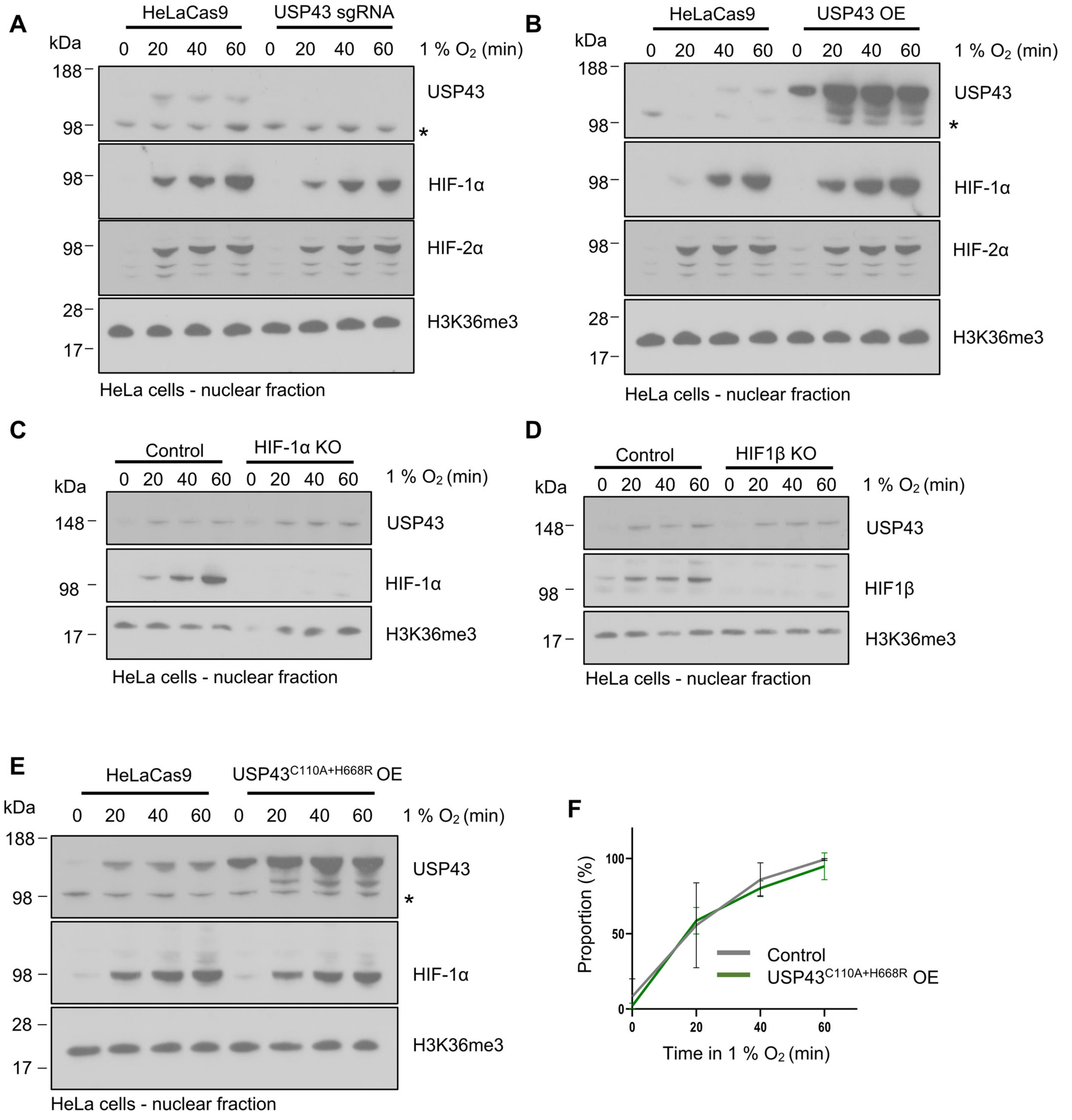

Figure EV4. USP43 regulates HIF-1α nuclear accumulation.

(**A**, **B**) Immunoblot of the nuclear fraction of HeLa-Cas9 or mixed population USP43 KO cells (**A**), USP43 overexpressing (OE) cells (**B**). (**A**) shows an extended immunoblot of Fig. 5C. Representative of three biological replicates. *non-specific band. (**C**) Immunoblot of the nuclear fraction of HeLa-Cas9 or HIF-1α KO cells incubated in 1% oxygen for 0–60 min. Samples were immunoblotted for USP43, HIF-1α and H3K36me3 as a loading control. Representative of three biological replicates. (**D**) As for (**C**) but in HIF1β KO cells. Representative of three biological replicates. (**E**, **F**) Immunoblot of the nuclear fraction of HeLa-Cas9 or $USP43^{C110A+H668R}$ OE cells (**E**) incubated in 1% oxygen for 0–60 min. Representative of three biological replicates. *non-specific band. Quantification of HIF-1α enrichment within the nuclear fraction in HeLa control or $USP43^{C110A+H668R}$ OE cells (**F**). HIF-1α levels relative to a stable histone mark (H3K36me3) were measured by immunoblot. $n = 3$ biologically independent samples, mean ± sd.

© The Author(s)

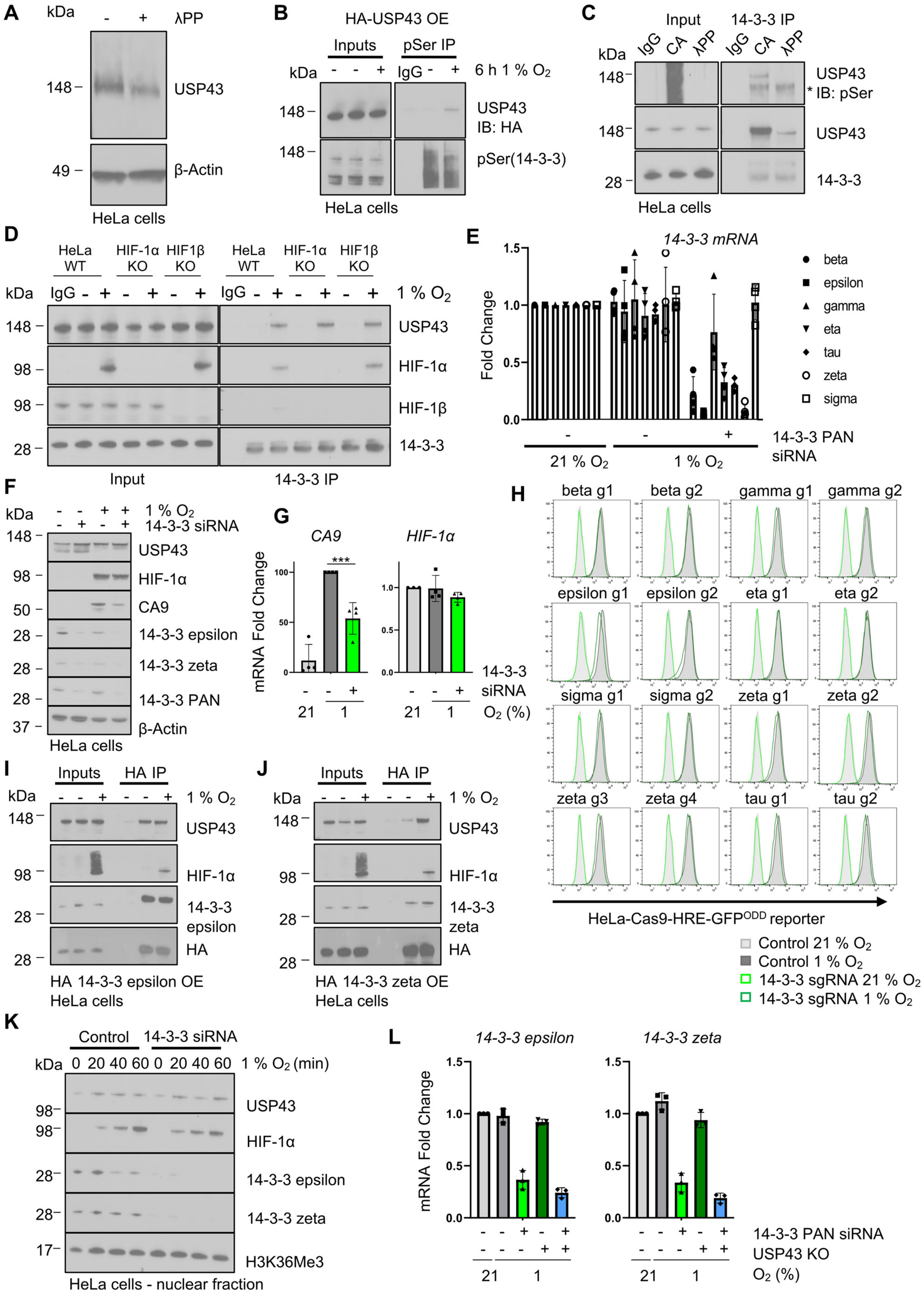
A
kDa
- + λPP
148 –
USP43
49 –
β-Actin
HeLa cells
B
HA-USP43 OE
Inputs
pSer IP
kDa - - + IgG - + 6 h 1 % O2
148 –
USP43
IB: HA
148 –
pSer(14-3-3)
HeLa cells
C
Input
14-3-3 IP
kDa IgG CA λPP IgG CA λPP
148 –
USP43
* IB: pSer
148 –
USP43
28 –
14-3-3
HeLa cells
D
HeLa WT
HIF-1α KO
HIF1β KO
HeLa WT
HIF-1α KO
HIF1β KO
kDa IgG - + - + - + IgG - + - + - + 1 % O2
148 –
USP43
98 –
HIF-1α
98 –
HIF-1β
28 –
14-3-3
Input
14-3-3 IP
E
14-3-3 mRNA
Fold Change
1.5
1.0
0.5
0.0
beta
epsilon
gamma
eta
tau
zeta
sigma
- - +
14-3-3 PAN siRNA
21 % O2
1 % O2
F
kDa
- - + + 1 % O2
- + - + 14-3-3 siRNA
148 –
USP43
98 –
HIF-1α
50 –
CA9
28 –
14-3-3 epsilon
28 –
14-3-3 zeta
28 –
14-3-3 PAN
37 –
β-Actin
HeLa cells
G
CA9
HIF-1α
mRNA Fold Change
***
100
50
0
1.0
0.5
0.0
- - +
- - +
14-3-3 siRNA
21 1
21 1
O2 (%)
H
beta g1
beta g2
gamma g1
gamma g2
epsilon g1
epsilon g2
eta g1
eta g2
sigma g1
sigma g2
zeta g1
zeta g2
zeta g3
zeta g4
tau g1
tau g2
HeLa-Cas9-HRE-GFPODD reporter
Control 21 % O2
Control 1 % O2
14-3-3 sgRNA 21 % O2
14-3-3 sgRNA 1 % O2
I
Inputs
HA IP
kDa - - + - - + 1 % O2
148 –
USP43
98 –
HIF-1α
28 –
14-3-3 epsilon
28 –
HA
HA 14-3-3 epsilon OE
HeLa cells
J
Inputs
HA IP
kDa - - + - - + 1 % O2
148 –
USP43
98 –
HIF-1α
28 –
14-3-3 zeta
28 –
HA
HA 14-3-3 zeta OE
HeLa cells
K
Control
14-3-3 siRNA
kDa 0 20 40 60 0 20 40 60 1 % O2 (min)
98 –
USP43
98 –
HIF-1α
28 –
14-3-3 epsilon
28 –
14-3-3 zeta
17 –
H3K36Me3
HeLa cells - nuclear fraction
L
14-3-3 epsilon
14-3-3 zeta
mRNA Fold Change
1.5
1.0
0.5
0.0
- - + - +
- - - + +
14-3-3 PAN siRNA
USP43 KO
21 1
O2 (%)

 © The Author(s)

Figure EV5. USP43 associates with 14-3-3 proteins to regulate HIF-1 signalling.

(**A**) Immunoblot of the phosphorylation-dependent electrophoretic mobility shift (PDEMS) assay of USP43, with or without lambda protein phosphatase (λPP) treatment. (**B**) Endogenous pSer(14-3-3 motif) was immunoprecipitated in HA-USP43 overexpressing HeLa cells incubated in 21 or 1% oxygen for 6 h. Representative of three biological replicates. (**C**) Endogenous 14-3-3 was immunoprecipitated in HeLa cells with or without Calyculin A (CA) treatment (100 nM, 37 °C for 30 min) or λPP (400 units, 30 °C for 30 min). Samples were immunoblotted for pSer(14-3-3 motif), USP43, and 14-3-3. Representative of three biological replicates. (**D**) Endogenous 14-3-3 was immunoprecipitated in wildtype, HIF-1α or HIF1β clonal KO HeLa cells grown in 21 or 1% oxygen for 6 h. Samples were immunoblotted for USP43, HIF-1α and HIF1β. Representative of three biological replicates. (**E**) qPCR of HeLa cells transfected with a 14-3-3 pan siRNA and incubated in 1 or 21% oxygen for 16 h. Expression of the seven *14-3-3* isoforms were analysed using individual primer pairs. $n = 3$ biologically independent samples, mean ± sd. (**F**) Immunoblot of control or 14-3-3 siRNA-depleted HeLa cells incubated in 21 or 1% oxygen for 16 h. Representative of three biological replicates. (**G**) qPCR of *CA9* and *HIF-1a* mRNA expression control or 14-3-3 siRNA-depleted HeLa cells incubated in 21 or 1% oxygen for 16 h. $n = 3$ biologically independent samples, mean ± sd. ***$P = 0.0002$, one-way ANOVA. (**H**) Mixed KO populations of HeLa HRE-^{ODD}GFP reporter cells to each 14-3-3 isoform were generated by lentiviral transduction of sgRNA (two sgRNAs for each isoforms, four sgRNAs for zeta isoform). Cells were incubated in 21 or 1% oxygen for 16 h and analysed by flow cytometry. (**I**, **J**) HeLa cells were transfected with HA-14-3-3 epsilon (**I**) or HA-14-3-3 zeta (**J**) and incubated in 21 or 1% oxygen for 6 h. The 14-3-3 isoforms were immunoprecipitated using the HA tag and immunoblotted for USP43. Representative of three biological replicates. (**K**) HIF-1α enrichment within the nuclear fraction in HeLa control or following 14-3-3 siRNA-mediated depletion. Cells were incubated in 1% oxygen for 0 to 60 min. (**L**) qPCR of *14-3-3 epsilon* and *zeta* expression in control or USP43 null cells, with or without 14-3-3 siRNA-mediated depletion. Cells were incubated in 21 or 1% oxygen for 6 h prior to lysis. $n = 3$, biologically independent samples, mean ± sd.

© The Author(s)
