# Supplementary material for: Deubiquitinating enzyme mutagenesis screens identify a USP43-dependent HIF-1 transcriptional response
Source: EMBO J. 2024 Jul 15;43(17):8. doi: 10.1038/s44318-024-00166-6 (PMC11377827; doi:10.1038/s44318-024-00166-6)
Supplement: Supplementary file 5 — Source data Fig. 1 [file screen sort report.pdf › Figure 1/F1 C GFP low sort/screen sort report.pdf]

Sort Report

System

Sort Start:	7/24/2019 10:48:27 AM	Server:	Utopex
Application:	BD FACS™ Sortware	Build:	1.2.0.108
Version:	1.2.0.142	Cytometer Model:	BD Influx System (USB)
ValComp:	7.5.1.3.16	Cytometer Serial #:	X0R400001

Details

Data Source:	Cytometer	Sort Mode:	1.0 Drop Pure
Nozzle Diameter (µm):	0.00	Drop Envelope:	1.0 Drop
Sheath Pressure (PSI):	0.00	Sort Objective:	Purify
Sort Device:	2 Tube Holder - 2 Way Sort	Phase Mask:	16/16
Piezo Amplitude:	4.60	Extra Coincidence Bits:	4
Drop Delay:	31.8076	Drop Frequency (kHz):	38.80

Sort Details

Name	Population	Event Limit	Event Count	Sort Count	Sort Rate	Aborts	Abort Rate	Efficiency	Time (sec)
Left	5% low	Unlimited	75,880,917	2,242,282	206	848,023	77	72.6%	10,877
Right	-	-	-	-	-	-	-	-	-
